# Assessing the Potential of Gabiroba Fruit Waste as an Oil Source via Ultrasound-Assisted Extraction: Physicochemical Parameters, Chemical Profile, and Biological Properties

**DOI:** 10.3390/molecules31203705

**Published:** 2026-10-16

**Authors:** Carolina Lilibeth Carvalho de Pinho, Renata Lázara de Araújo, Suelen Ávila, Giselle Maria Maciel, Isadora da Silva Zanzarini, Vivian Rotuno Moure, Glaucio Valdameri, Fabiane Oliveira Farias, Cristiane Vieira Helm, Marcos Rogério Mafra, Luciana Igarashi-Mafra

**Affiliations:** 1 Department of Chemical Engineering, Postgraduate Program in Food Engineering, Federal University of Paraná, Curitiba 81531-980, PR, Brazil; carolinacpinho@gmail.com (C.L.C.d.P.); fabianefarias@ufpr.br (F.O.F.); marcos.mafra@ufpr.br (M.R.M.); luciana.igarashi@gmail.com (L.I.-M.); 2 Institute of Exact and Earth Sciences, School of Food Engineering, Federal University of Mato Grosso, Barra do Garças 78605-091, MT, Brazil; 3 Postgraduate Program in Food and Nutrition, Health Science Sector, Federal University of Paraná, Campus III, Curitiba 80210-170, PR, Brazil; suelenavila@gmail.com; 4 Biotechnology Laboratory, Department of Chemistry and Biology, Federal University of Technology—Paraná (UTFPR), Curitiba 81280-340, PR, Brazil; gisellemariam@gmail.com; 5 Postgraduate Program in Pharmaceutical Sciences, Laboratory of Cancer Drug Resistance, Federal University of Parana, Curitiba 80210-170, PR, Brazil; isadorazanzarini@ufpr.br (I.d.S.Z.); vivian.moure@ufpr.br (V.R.M.); gvaldameri@ufpr.br (G.V.); 6 Brazilian Agricultural Research Corporation (Embrapa Florestas), Colombo 83411-000, PR, Brazil; cristiane.helm@embrapa.br

**Keywords:** gabiroba waste, functional oils, sustainable extraction, phytochemicals, bioactive lipids, food by-product valorization

## Abstract

This study aimed to evaluate the impact of ultrasound-assisted extraction (UAE) on the chemical composition and biological activities of gabiroba waste oil (GWO), compared to conventional Soxhlet extraction (SE). GWO was characterized for carotenoid content and analyzed by GC-MS for fatty acids (FAs) and sesquiterpenes. Total phenolic content (TPC), flavonoid content (TFC), and antioxidant (ABTS, DPPH, FRAP), antimicrobial, and antiglycemic activities were evaluated. Both methods yielded similar carotenoid content (954–1110 μg β-carotene/g). UAE enhanced phytochemical extraction, with 33% higher TPC (281 mg GAE/100 g), 50% more TFC (193 mg QE/100 g), and superior antioxidant activity (ABTS-12 μmol TE/g for hydrophilic extract, 109 μmol TE/g for lipophilic extract). UAE produces a high-quality oil with higher yield (36.6%) and lower acidity index (12.5%) compared to SE. Oleic acid was the predominant FA, followed by palmitic and linoleic acids, with β-cadinene as the major terpene. UAE oil exhibited a 21% increase in unsaturated FA and a 36% reduction in saturated FA compared to SE. UAE oil had greater antimicrobial activity for *S. aureus* (MIC-3.9 mg/mL) and higher α-amylase (IC_50_—316 μg/mL) and α-glucosidase (IC_50_—45 μg/mL) inhibitory potential. UAE proved to be an efficient, sustainable method to obtain phytochemical-rich oil from food residues, valorizing gabiroba waste for pharmaceutical and functional food applications.

## 1. Introduction

*Campomanesia xanthocarpa*, commonly known as gabiroba, is a native Brazilian fruit belonging to the Myrtaceae family, widely distributed across the southern and central-western regions, particularly in the Cerrado and Atlantic Forest biomes [1,2]. The fruits are small, globular berries that turn yellow and sweet upon ripening, traditionally used in the artisanal production of sweets, beverages, and jams [3,4]. Beyond their pleasant flavor, gabiroba fruits present important nutritional and functional properties, being composed of carbohydrates (7.8–17.7%), fibers (6.3–9.9%), proteins (1.1–5.5%), and lipids (0.6–3.7%). Moreover, the fruit matrix represents a rich natural source of ascorbic acid (up to 30.58 mg g^−1^), minerals, phenolic compounds, and carotenoids [3,5,6]. Previous investigations focusing on different anatomical parts of *C. xanthocarpa*, including leaves, pulp, and seeds, have demonstrated significant antioxidant, antimicrobial, anti-inflammatory, antihypercholesterolemic, antitumor, and antidiabetic activities [7,8,9,10,11]. However, despite this well-documented functional potential, the commercial exploitation and industrial processing of gabiroba remain limited [4,12]. Processing or direct consumption of gabiroba generates about 40% of agri-food waste (peels, seeds, and calyx) [4], which is increasingly recognized as a valuable source of biologically active constituents, offering strategic opportunities for sustainable practices and initiatives aligned with the principles of the circular economy.

While significant research has focused on the bioactive profile and biological activities of aqueous and alcoholic extracts from the pulp and leaves of gabiroba [4,7,8,13,14,15], comprehensive data regarding the lipid fraction recovered, particularly from fruit waste, remain underexplored. To date, a single study has investigated the oil extracted specifically from gabiroba seeds [16], reporting a profile rich in bioactive fatty acids, notably oleic, linoleic, and palmitic acids. However, the oil recovered from whole gabiroba fruit processing waste remains unexplored, leaving a clear scientific gap regarding its chemical composition and functional potential.

Researchers have investigated the chemical and physical properties of oils derived from non-conventional plant species to identify alternative lipid sources with distinct nutritional, functional, and technological characteristics, capable of expanding application possibilities and meeting the demands for new products, boosting their industrial application [17,18]. The valorization of gabiroba waste as an alternative source of oil relies heavily on the extraction method used, which directly influences yield and compound integrity. Soxhlet extraction (SE) is conventionally employed due to high yields; however, the prolonged extraction times and elevated temperatures can degrade sensitive phytochemical compounds. Additionally, other limitations of SE include high energy consumption and excessive use of organic solvents. In contrast, ultrasound-assisted extraction (UAE) has emerged as an efficient, eco-friendly alternative, offering faster extraction times, reduced solvent consumption, lower energy requirements, and better preservation of heat-sensitive compounds. This technique utilizes high-frequency sound waves to generate cavitation bubbles, enhancing mass transfer and improving the extraction of phytochemical compounds from plant materials [19,20].

Considering the nutritional potential of gabiroba, the importance of new natural sources of bioactive compounds, and the need for sustainable extraction methods, this study aims to assess the impact of ultrasound-assisted extraction on the chemical composition and biological activities of gabiroba waste oil, compared to conventional Soxhlet extraction.

## 2. Results and Discussion

### 2.1. Proximate Composition of Gabiroba Waste

**Figure 1:**
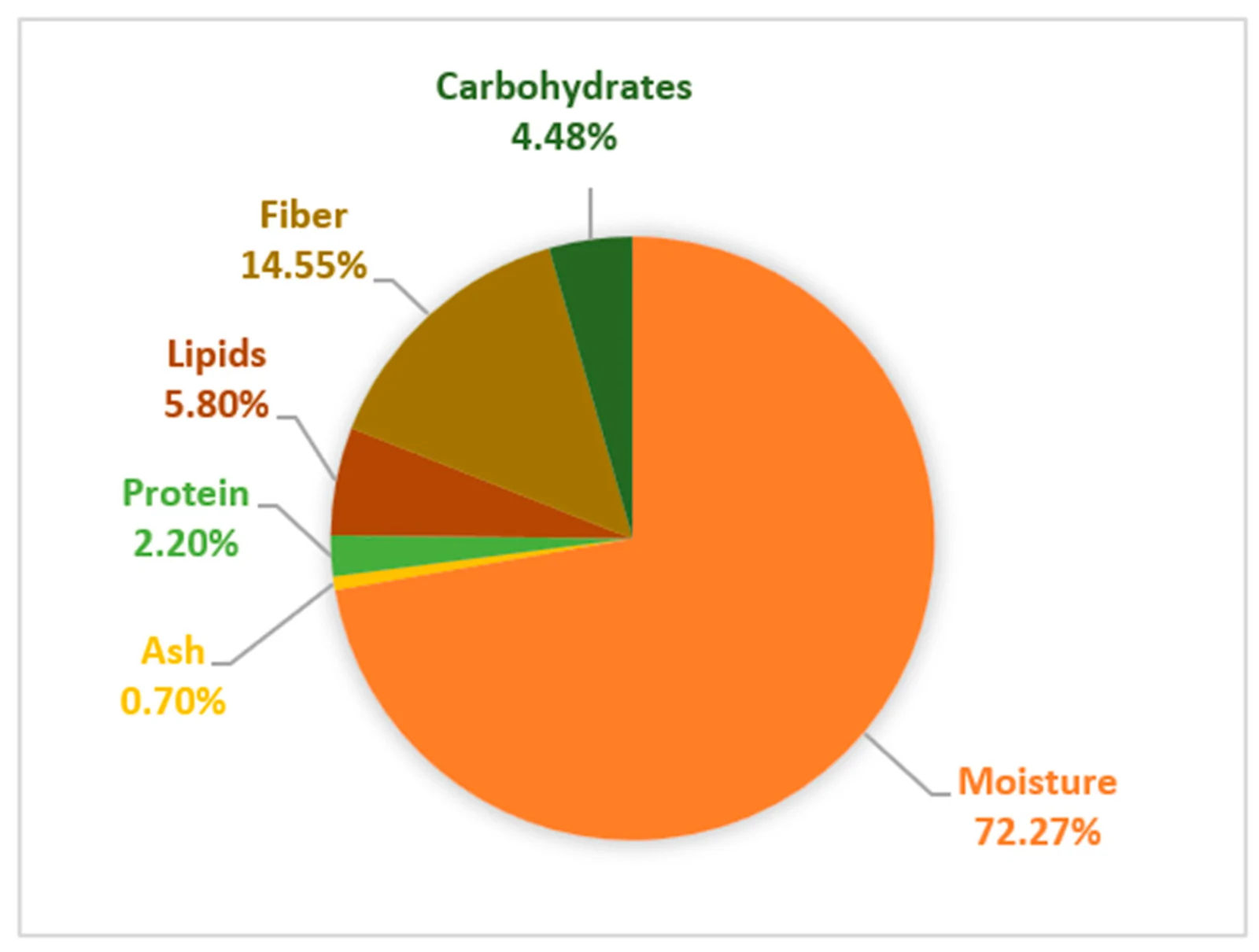
Proximate composition of gabiroba waste on a wet weight basis.

Water was the main constituent of the gabiroba waste (GW), accounting for 72.27% ± 0.1% (Figure 1). Among the pulp macronutrients, dietary fiber (14.55 ± 0.29%) and lipids (5.80 ± 0.59%) were the major components, whereas carbohydrates (4.48%) and proteins (2.20 ± 0.08%) were present in lower amounts. These results are consistent with those reported for the proximate composition of *Campomanesia adamantium* (green gabiroba) residue (moisture: 63.7%; ash: 0.74%; protein: 3.17%; lipids: 5.33%; fiber: 24.05%; and carbohydrates: 3%) [21]. Also, GW exhibits higher lipid, protein, and fiber contents compared to the whole fruit (1.3–1.9% of lipids, 1.1% of proteins, 6.3–9.9% of fibers) [3,6]. According to the Agência Nacional de Vigilância Sanitária (ANVISA), which establishes a daily fiber intake of 25 g in a 2000 kcal diet, GW can be classified as a high-fiber content material, since it provides 20% of the recommended daily value of fiber [22]. Fibers such as galactomannans and pectins, previously identified in the pulp of the gabiroba fruit, are promising polysaccharides due to their gelling and stabilizing properties for the food industry and pharmacological applications, such as immunoregulatory, anti-inflammatory, hypoglycemic, antioxidant and antitumor activities [7,23,24,25].

### 2.2. Fatty Acid and Sesquiterpene Profile

GW was used to obtain oil employing ultrasound-assisted extraction (UAE) and Soxhlet (SE) techniques. Gas chromatography analysis revealed the presence of over 30 compounds (Table 1), with fatty acids accounting for more than 70% of the total identified substances. The identification of the compounds was supported by the raw total ion chromatograms (TICs) and experimental mass spectra obtained by GC-MS, which are provided in the Supplementary Materials (Figures S1–S4). In both GWO extraction methods, the predominant fatty acids were oleic acid (C18:1), followed by palmitic acid (C16:0) and linoleic acid (C18:2). These results are consistent with those reported by Santos et al. [16], who identified oleic, palmitic, and linoleic acids as the primary fatty acids in *C. xanthocarpa* seed oil, comprising 60.8%, 13.9%, and 14.1%, respectively.

Monounsaturated fatty acids (MUFAs) constitute the predominant group of fatty acids in GWO, with the UAE oil showing a 21% increase in unsaturated fatty acid content compared to Soxhlet extraction (SE), accompanied by a 36% reduction in saturated fatty acids. The observed variation in the relative fatty acid profile between UAE and SE can be attributed to differences in extraction selectivity and thermal preservation. The milder extraction conditions and shorter extraction time employed in the UAE minimize thermal and oxidative degradation of unsaturated fatty acids, which are more susceptible to oxidation. Conversely, the prolonged exposure to high temperatures and continuous reflux inherent to SE can induce partial thermal degradation of double bonds in unsaturated species [26]. Furthermore, the energy generated by ultrasonic waves causes cavitation bubbles to form, expand, and collapse as the waves propagate through the solvent medium; this disrupts cell membranes and rapidly releases fluid, membrane-associated lipid fractions that are naturally enriched in unsaturated chains [27,28]. MUFAs and saturated fatty acids are more resistant to high temperatures and rancidity than polyunsaturated fatty acids [20]. In addition to MUFAs, the fatty acid composition of GWO also includes linoleic acid, an omega-6 (ω-6) polyunsaturated fatty acid essential for human health, as it influences the prevalence and severity of cardiovascular diseases, diabetes, cancer, and age-related functional decline [29]. Furthermore, small amounts of myristic, stearic, and eicosanoic acids were detected, and elaidic acid, a trans-isomer of oleic acid, was present at concentrations below 3%.

The biological functions of oils obtained from native Brazilian fruits have been extensively studied. Cumaru seed oil showed antimicrobial and antioxidant properties [30], and buriti oil has immunomodulatory effects against enteropathogenic *Escherichia coli* [31].

The volatile compounds detected in the GWO predominantly belong to the terpene class, with sesquiterpenes being the most prevalent. Among these, β-cadinene was identified as the most abundant terpene in both UAE and SE methods. Terpenes are the main components of essential oils and have been previously found in essential oils from the fruits and seeds of *C. xanthocarpa* [3,32]. Capeletto et al. [15] reported sesquiterpenes such as caryophyllene (17.83%), γ-cadinene (9.58%), and α-cadinol (8.83%) as the major compounds in extracts of *C. xanthocarpa* seeds obtained by supercritical CO_2_ and compressed n-butane. The terpene profile presented by the GWO is similar to that found by Cardoso et al. [33] for the hexane fractions of the fruits of *C. pubescens* and *C. adamantium*. Aromatic compounds, such as sesquiterpenes, are associated with the characteristic flavor and aroma of fruits, which supports their application as flavoring agents in foods and beverages [3].

### 2.3. Physicochemical Characterization of Gabiroba Waste Oil

UAE showed a slight increase (*p* = 0.02) in extraction yield when compared to SE (Table 2). In SE, time, temperature, solvent type, number of cycles, and solvent/solute interactions influence the extraction of biomolecules from plant matrices; that is, at its boiling point, the surface tension and viscosity of the solvent are reduced, facilitating access to the active sites of the matrix and favoring the solubilization of the compounds of interest [34]. On the other hand, the ultrasound technique was applied due to its efficiency and low cost since it does not require high temperatures and reduces extraction time, preserving the compounds present in the plant matrix.

The higher oil yield observed for UAE compared to SE can be attributed to the physical and mechanical phenomena driven by acoustic cavitation, which enhance the contact between the solvent and the solid matrix and facilitate mass transfer [35]. As ultrasonic waves propagate through the sample, they generate rapid alternating compression and expansion cycles (sponge effect) within the plant tissue, producing mechanical stresses that can promote cell disruption and the formation of microchannels and micropores, favoring solvent penetration into the plant matrix and the release of intracellular oil [36]. Similar to Tan et al. [19] for avocado oil, UAE outperformed SE in both yield and extraction efficiency of GWO.

Regarding the acidity index, UAE resulted in a better-quality oil with a lower content of free fatty acids. Conversely, SE resulted in an acidity index of 14.1%, around 13% higher than UAE-based oil. High temperatures contribute to an increase in the hydrolytic rancidity of the oil, with consequent de-esterification of fatty acids and increased concentrations of free fatty acids [31]. Moreover, when the oil is exposed to high temperatures, its density can be affected due to oxidation and polymerization reactions [37]. This effect was observed for GWO, in which the density increased from 922.8 Kg m^−3^ for the UAE oil to 932.4 Kg m^−3^ for the SE oil. The refractive index (IR) for GWO obtained by UAE and SE were 1.4802 and 1.4894, respectively. IR of fats and oils is related to their average degree of unsaturation and it is helpful in observing the progress of reactions such as hydrogenation and isomerization [38]. Higher IR values indicate a greater possibility of deterioration due to oxidation [39].

### 2.4. Carotenoids, Phenolic Compounds and Antioxidant Capacity

**Figure 2:**
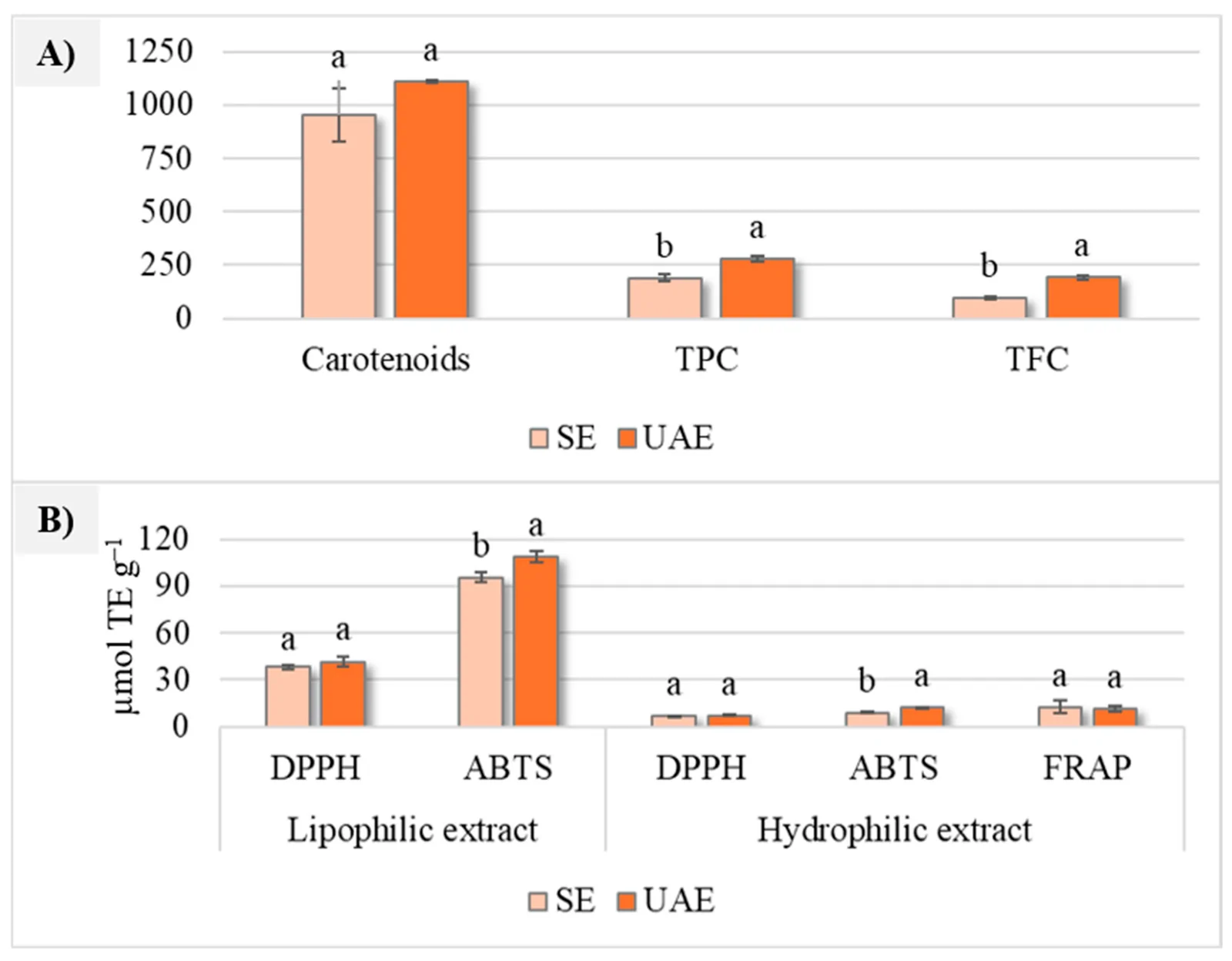
Carotenoid content (μg of β-carotene g^−1^), total phenolic compounds (TPC) (mg GAE 100 g^−1^), total flavonoid content (TFC) (mg QE 100 g^−1^) (**A**), and antioxidant activity by DPPH, ABTS, and FRAP assays (**B**) of GWO. Equal letters do not differ by Tukey’s test (*p* > 0.05).

Figure 2A shows that the gabiroba waste oil (GWO) obtained exhibited high carotenoid levels; however, no significant differences (*p* > 0.05) were observed between UAE and SE, indicating that both extraction methods were similarly effective for carotenoid recovery. The carotenoid levels in the GWO present average values of 1032.39 ± 8.73 μg β-carotene g^−1^, which were comparable to that reported for buriti oil (760 μg β-carotene g^−1^) [31], approximately twice that reported for pequi oil (550 μg β-carotene g^−1^) [40], and 13-fold higher than that reported for gabiroba seed oil (79 μg β-carotene g^−1^) [16]. Carotenoids have important biological functions, being widely used industrially as natural colorants, in addition to providing several health benefits due to their antioxidant properties and their role as a precursor to vitamin A [14].

UAE exhibited 33% greater efficiency in obtaining total phenolic compounds (TPC: 280.79 ± 13.74 mg GAE 100 g^−1^) and 50% higher levels of total flavonoids (TFC: 192.83 ± 8.92 mg QE 100 g^−1^) compared to SE, which yielded TPC of 188.16 ± 16.42 mg GAE 100 g^−1^ and TFC of 97.39 ± 6.86 mg QE 100 g^−1^ (Figure 2A). Phenolic and flavonoid contents were compared with those of lipid fractions recovered from other non-conventional oil sources. The TPC of GWO was lower than that reported for baru seed oil (685–1315 mg GAE 100 g^−1^) [41]. Conversely, the TPC of GWO was higher than those reported for cumaru seed oil (22 mg GAE 100 g^−1^) [30], pequi oil (4.40 mg GAE 100 g^−1^) [40], and gabiroba seed oil (43 mg GAE 100 g^−1^) [16]. Similarly, GWO showed significantly higher values of TFC than those reported for pequi oil (1.10 mg QE 100 g^−1^).

UAE demonstrated the highest antioxidant activity as measured by the ABTS method, with values of 12.08 ± 0.14 μmol TE g^−1^ for the hydrophilic extract and 108.87 ± 3.64 μmol TE g^−1^ for the lipophilic extract (Figure 2B). The higher temperature and longer extraction time used in SE may have led to the degradation of some antioxidant compounds, which could have substantially contributed to the observed differences in antioxidant activity. However, the extraction technique did not significantly affect FRAP or DPPH values.

When comparing the antioxidant activity of lipophilic and hydrophilic extracts, lipophilic extracts showed a fivefold increase in DPPH activity and a tenfold increase in ABTS activity (Figure 2B). Assays such as DPPH, ABTS, and FRAP have different mechanisms of action, and results vary depending on the chemical composition of the extracts and the reactivity of the antioxidants in each method [31]. The observed differences in antioxidant activity across methods and extraction techniques resulted from the varying reactivity of the extract components with the radicals present in each assay.

These phytochemicals play a crucial role in lipid matrices by acting as natural antioxidants. They exert a protective effect on unsaturated fatty acids, scavenging free radicals and mitigating oxidative lipid degradation during storage and processing, and may also contribute to enhancing the biological activities associated with the oils [20].

### 2.5. Antimicrobial Activity

The antimicrobial activity of GWO varied according to the extraction method and target microorganism, as can be observed in Table 3. *S. aureus* was more susceptible to UAE oil compared to SE oil. Conversely, *B. cereus* was more susceptible to SE oil, whereas higher concentrations were needed for UAE oil. Evidence suggests that fatty acids act as detergents in the amphipathic structure of bacterial cell membranes, impairing energy generation processes [42]. Also, terpenes found in GWO are bioactive compounds capable of inhibiting protein and DNA synthesis and promoting cell membrane rupture [43]. Similarly, sesquiterpenes from *C. xanthocarpa* seed extracts have previously shown inhibitory effects against resistant bacteria like Pseudomonas aeruginosa, with MIC and MBC between 5 and 40 mg mL^−1^ [15].

The antifungal activity of GWO against *C. albicans* was similar for both extraction methods. SE and UAE oils exhibited an MIC of 15.63 mg mL^−1^. However, UAE oil showed superior fungicidal activity, with an MBC of 31.25 mg mL^−1^ compared to 62.50 mg mL^−1^ for SE oil. Fatty acids, such as oleic, palmitic, and linoleic acids, found in GWO, along with lauric and stearic acids, are known for their antifungal potential [44].

GWO did not show any antimicrobial effect against *S. enterica*, *E. coli* and *L. monocytogenes*, probably due to structural differences in their cell walls. The outer membrane of Gram-negative bacteria, such as *E. coli* and *S. enterica*, contains a lipopolysaccharide layer that acts as a hydrophilic barrier, limiting the diffusion of hydrophobic compounds [45]. Additionally, *L. monocytogenes* is a Gram-positive bacterium with high resistance to environmental stress, which may also hinder the action of antimicrobial compounds [46].

Despite this selective activity, the antimicrobial potential of GWO is promising. The different antimicrobial performance between extraction methods may reflect variations in the chemical composition of the oils, which could influence their biological activity.

### 2.6. Antiglycemic Activity

**Figure 3:**
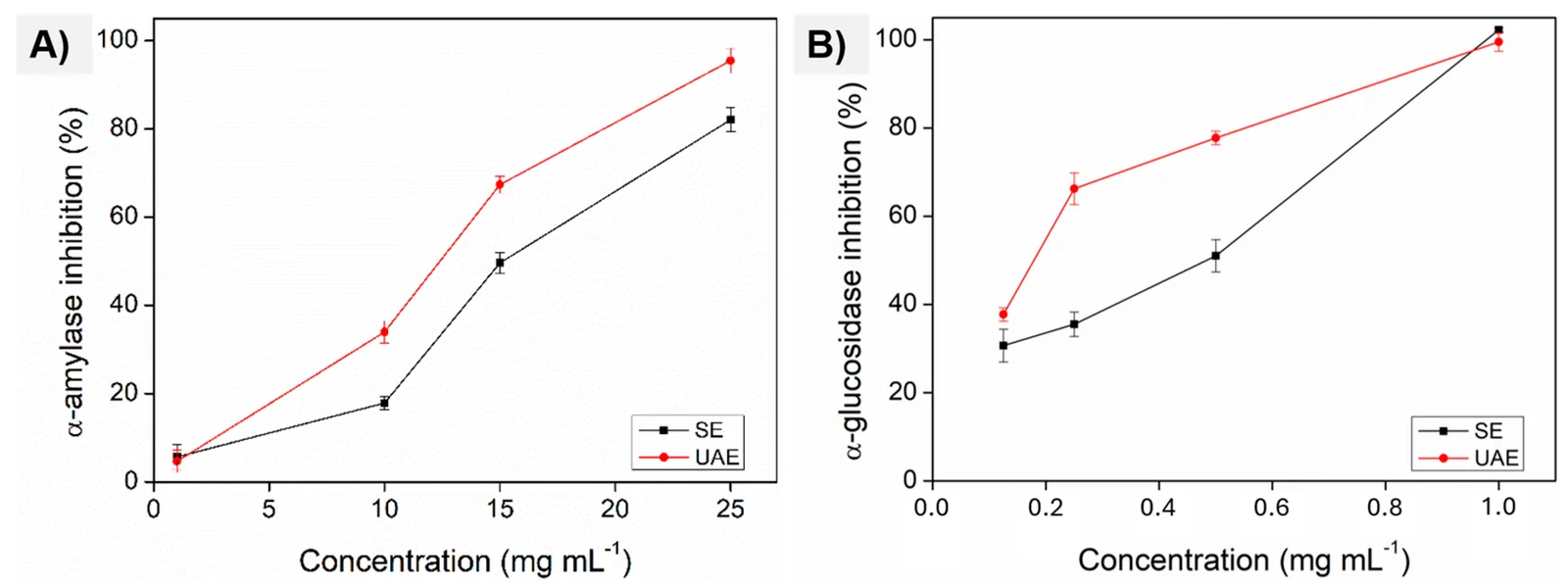
Inhibitory activity of α-amylase (**A**) and α-glucosidase (**B**) enzymes by GWO obtained through SE and UAE.

The antiglycemic activity is focused on the inhibition of α-amylase and α-glucosidase enzymes, responsible for the digestion of soluble carbohydrates. The effectiveness of GWO in inhibiting α-amylase (Figure 3A) and α-glucosidase (Figure 3B) enzymes exhibited a dose-dependent relationship. The compounds obtained by UAE potentiated the α-amylase inhibitory effect, mainly at the highest concentrations, with an inhibition of 95% for UAE and 82% for SE. The IC_50_ values highlighted a significant difference: 316 μg mL^−1^ for the UAE oil, compared to 400 μg mL^−1^ for the SE oil; that is, the oil obtained through ultrasound extraction is more efficient, requiring lower concentrations to achieve 50% enzyme inhibition. Methanolic extracts of gabiroba fruits rich in phenolic compounds showed inhibition of α-amylase activity with an IC_50_ of 26 μg mL^−1^ in the reaction [8], a more significant effect compared to the present work. This difference is associated with both the characteristics of the raw material and the solvent employed, which influence the chemical composition and bioactive properties of the extracts.

On the other hand, both oils reached 100% inhibition of α-glucosidase at the highest concentration. However, considering the IC_50_ values, UAE oil (IC_50_: 45 μg mL^−1^) demonstrated superior inhibition potential compared to SE oil (IC_50_: 122 μg mL^−1^). At a concentration of 0.125 mg mL^−1^, the UAE oil exhibited a more significant inhibitory effect on α-glucosidase than on α-amylase, achieving 37% inhibition. Acarbose is the standard compound often used to slow carbohydrate digestion by inhibiting digestive enzymes and has IC_50_ values of 24 and 58 μg mL^−1^ for α-amylase and α-glucosidase, respectively [47]. This suggests that the UAE oil is more potent in inhibiting α-glucosidase at lower concentrations, emphasizing its potential as a more effective supplement or additive for controlling postprandial blood glucose levels.

The antiglycemic effect is a known property of aqueous and alcoholic extracts of leaves, seeds, and fruits of *C. xanthocarpa* [8,10,13,48]. Extracts obtained with pressurized solvent from *C. xanthocarpa* seeds reduced blood glucose levels in rats, with this effect being attributed to the terpenes found, such as caryophyllene, eudesmol, guaiol, α-selinene and β-cadinene [10], many of which were also identified in GWO.

The observed hypoglycemic effects of GWO, attributed to enzyme inhibition, align with findings in the literature that have linked such activities to the presence of fatty acids, reported as inhibitors of carbohydrate-hydrolyzing enzymes like α-glucosidase and α-amylase, thereby delaying glucose absorption [49]. Moreover, lipid–phenolic interactions have been shown to contribute to antioxidant and α-amylase inhibition activities [50]. Therefore, the enzymatic inhibition observed in GWO may stem from a synergistic interaction of various compounds present in the oils, further supporting its potential as an innovative natural additive for controlling glucose metabolism.

### 2.7. Anticancer Activity

**Figure 4:**
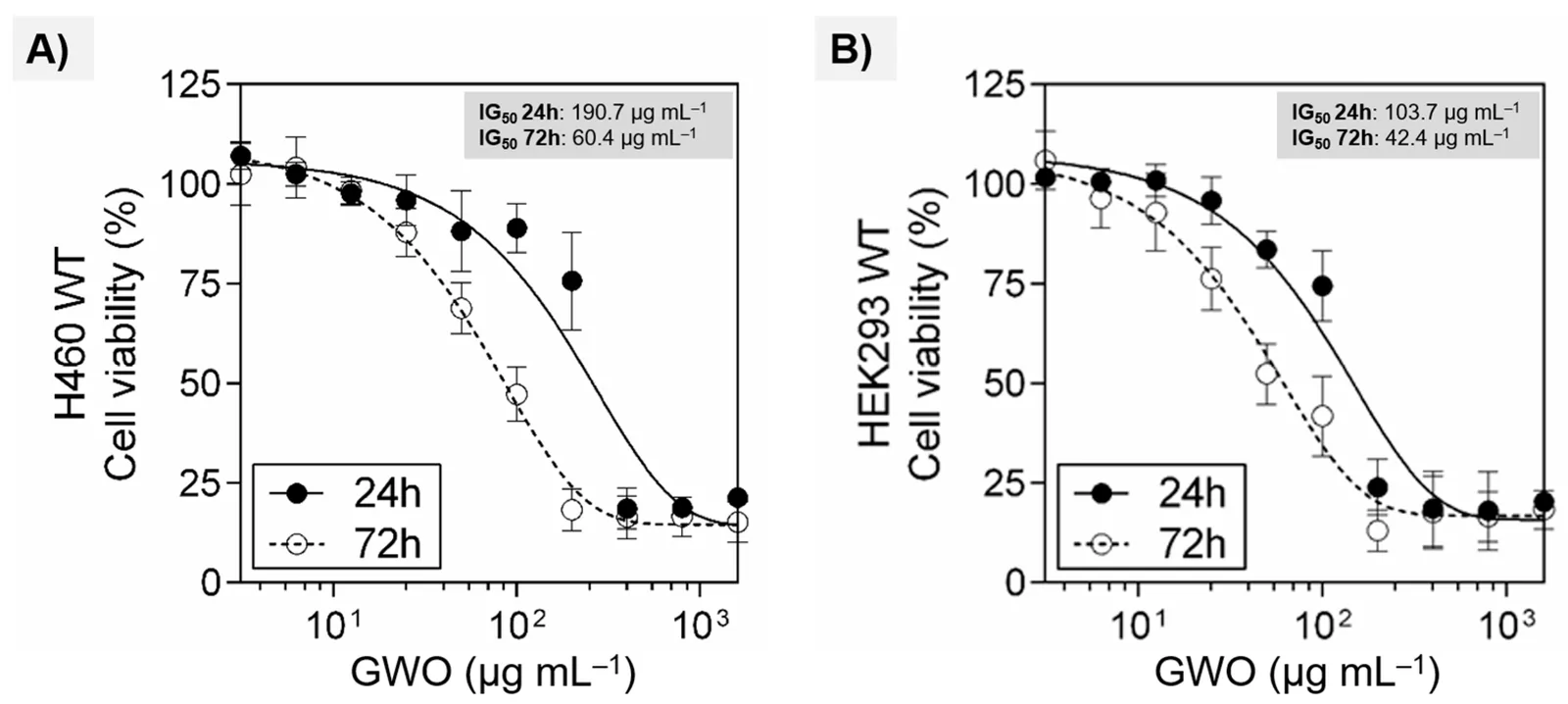
Cytotoxic effect of GWO extracted by UAE. Cell viability assay performed using the MTT method. HEK293 wild-type (**A**) and H460 wild-type cells (**B**) were exposed for 24 and 72 h with increasing concentrations of the compounds (3.125–1600 µg mL^−1^). Data represents mean ± SD of at least three independent experiments. The viability curves were fitted using GraphPad Prism 6 software.

The cytotoxic effect of UAE oil was evaluated in human fibroblast (HEK293 WT) and lung cancer (H460 WT) cell lines using the MTT assay. Increasing the concentration of GWO and exposure time decreased the number of viable cells, indicating a time- and dose-dependent cytotoxic effect. As shown in Figure 4A, H460 cells responded differently depending on the incubation period when lower oil concentrations were tested. The cytotoxic effect was most severe after 72 h of exposure. However, at higher concentrations (400–1600 µg mL^−1^), cell viability was equally reduced to less than 25% after 24 and 72 h of exposure. According to the IG_50_ (concentration required to reduce 50% of cell viability), prolonged exposure to GWO was 3 times more cytotoxic.

Similar behavior was observed for HEK293 cells; cytotoxicity was higher after 72 h of incubation (Figure 4B). At a concentration of 100 μg mL^−1^, cell viability remained above 75% after 24 h of exposure and below 50% after 72 h. At this concentration and regardless of exposure time, GWO reduced cell viability to less than 25%.

The results presented by UAE oil were more significant than those presented by pectins extracted from gabiroba pulp, which reduced the metabolic activity of human glioblastoma cells by 80% at a concentration of 25 μg mL^−1^ [7]. It was demonstrated that fatty acids such as oleic and palmitic acid, present in GWO, have good affinity for inhibiting the myeloid cell leukemia protein 1 (Mcl-1) protein, pointing to the anticancer potential of fatty acids [51]. Additionally, unsaturated fatty acids known for inducing apoptosis in tumor cells may further enhance the generation of free radicals and lipid peroxidation, strengthening GWO anticancer effects [52]. Furthermore, flavones (5-hydroxy-7-methoxy-8-methyl) isolated from extracts of gabiroba fruits inhibited the growth of H460 cells by 27% and renal tumor cells by 46% [53]. Phytochemicals, such as phenolics and carotenoids, can act as pro-oxidants, inducing free radical production that combats tumor proliferation [54].

From a pharmacological perspective, it is important to highlight that the cytotoxicity of a compound at lower concentrations is one of the most valuable characteristics for clinical applications [7]. The IG_50_ value for doxorubicin in H460 cells, the same cell line used in this work, corresponds to 0.055 µM (0.03 µg mL^−1^) [55]. GWO at a concentration of 50 µg mL^−1^ reduced cell viability below 70% after 72 h of exposure. These results are promising, as a compound could be considered cytotoxic when the cell viability is reduced by more than 30% [56]. However, an important characteristic of cytotoxicity is selectivity, so the cytotoxic agent should induce a toxic effect on cancer cells, but it should not affect normal ones [57,58]. The IG_50_ values demonstrate that GWO was approximately 1.8 times more cytotoxic to fibroblasts than to lung cancer cells. These results reinforce the need to evaluate the selectivity of the interaction with cancer cells in the screening of anticancer drug candidates. Therefore, further investigation into the chemical composition of GWO and its cytotoxic profile is important before indicating it as a potential agent in anticancer therapy. 

## 3. Materials and Methods

### 3.1. Preparation and Proximate Composition of Gabiroba Waste

**Figure 5:**
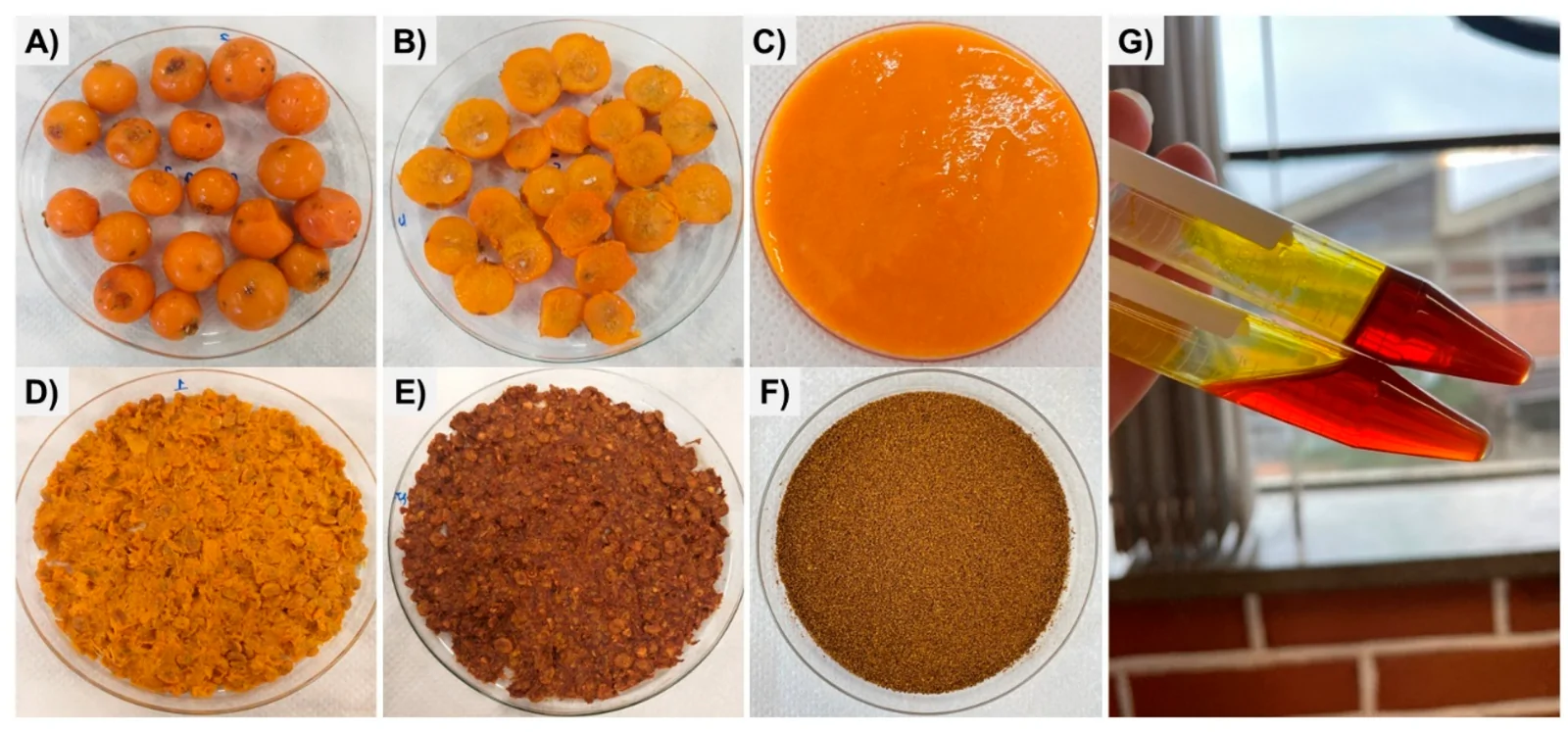
Gabiroba fruit (**A**), interior of the fruit (**B**), pulp (**C**), fresh residue (**D**), dried residue (**E**), residue flour (**F**), lipidic fractions (**G**).

Ripe gabiroba fruits were collected in the city of Irati, Paraná state, Brazil (S25°27′56″; W50°37′51″) (Figure 5A,B). The fruits were washed, sanitized, and then stored at −18 °C. The fruits were thawed and subjected to pulping (Figure 5C) using a pulper (Braesi, model DES-20, Caxias do Sul, RS, Brazil). Gabiroba waste (GW) was collected (Figure 5D), dried at 40 °C in an oven with air circulation for 48 h (Figure 5E), and subsequently ground in a laboratory mill (Cadence, model MDR302, China). The ground material was sieved through a Tyler series 20 mesh sieve, collecting particles with a diameter smaller than 0.5 mm. The raw material (Figure 5F) was stored under vacuum at 25 ± 5 °C, protected from light, until further use.

Proximate composition analyses of GW were performed in triplicate using protocols established by AOAC [59]. Moisture content was obtained by oven drying at 105 °C to constant mass (AOAC 926.12); total proteins were determined by the Kjeldahl method, with a nitrogen-to-protein conversion factor of 6.25; fixed mineral residues were obtained by incineration in a muffle furnace at 550 °C (AOAC 945.46); and dietary fiber was obtained using the enzymatic-gravimetric method (AOAC 991.43). Lipid content was assessed by cold extraction, based on the method of Bligh and Dyer [60]. Total carbohydrate content was calculated by the difference in mass.

### 3.2. Extraction Procedures

The ultrasonic-assisted extraction (UAE) of gabiroba waste oil (GWO) was performed according to the methodology described by Goh et al. [61], with modifications. An ultrasound system equipped with a high-intensity probe (130 W, 20 kHz—Sonics & Materials, Inc., model Sonics Vibra-Cell VCX 130, Newtown, CT, USA) was used. The amplitude of ultrasonic vibrations was set at 100% of the nominal power. The extraction was carried out in an Erlenmeyer flask containing 20 g of sample and 50 mL of hexane for 30 min. The ultrasonic probe was directly immersed in the mixture, with the tip fully submerged in the solvent. Ultrasonication was applied in pulses of 30 s on and 30 s off to minimize overheating, and an ice bath was employed to maintain the temperature below 30 °C. After sonication, the mixture was filtered, and the raw material was subjected to exhaustive extraction with 50 mL of fresh solvent. This procedure was repeated three times, and all filtrates were pooled in a single vial. For comparison, Soxhlet extraction (SE) was performed using 20 g of sample wrapped in filter paper and placed inside a cartridge. The extraction was carried out with 150 mL of hexane recirculated for 9 h. The extraction yield (%) was calculated by determining the ratio of the mass of the extract to the mass of the raw material, multiplied by 100.

After extraction by both methods, the solvent was removed using a rotary vacuum evaporator at 35 °C under reduced pressure (5 psi). The extracted oil (Figure 5G) was stored in amber glass vials under refrigeration until further analysis. Lipophilic and hydrophilic extracts were obtained from GWO extracted by UAE and SE for the determination of carotenoids, phenolic compounds, flavonoids and antioxidant activity. Lipophilic extracts were obtained by adding 20 mL of ice-cold acetone to 1 g of oil, followed by continuous agitation in an ultrasonic bath for 10 min. After extraction, the mixture was filtered and subjected to liquid–liquid partitioning with petroleum ether and water. The ether phase was collected and used for subsequent analyses [40]. To obtain the hydrophilic extracts, 1 g of oil was extracted with 5 mL of hexane. Subsequently, 5 mL of methanol/water (60:40, *v*/*v*) was added, and the mixture was vigorously agitated for 2 min. The hydromethanolic fraction was collected and used for the analyses [62].

### 3.3. Physical–Chemical Parameters

Acidity index (method 325/IV) was evaluated following the methods proposed by Instituto Adolf Lutz [63]. The refractive index and density were measured at 20 °C, using an Abbe refractometer (Biobrix, Tzaneen, South Africa) and an Anton Paar vibrating-tube density meter (Graz, Austria, DMA 500 M), respectively. The acidity index was expressed as a percentage of free fatty acids, and the density as kg m^−3^.

### 3.4. Fatty Acids and Sesquiterpenes Composition by GC/MS

The identification of fatty acids and sesquiterpene compounds was performed using a gas chromatograph coupled to a triple quadrupole mass spectrometer detector (GC/MS-TQ8040, Shimadzu, Kyoto, Japan). The injection volume was 1 µL of the previously esterified samples [64]. During operation, the detector was maintained at 280 °C and the injector at 250 °C. The analysis was carried out in split mode (1:50) and separation was achieved using an SH-Rtx-5MS column (Shimadzu) (30 m × 0.25 mm × 0.25 μm). The oven was set to 50 °C for 1 min, after which it was heated at 25 °C min^−1^ to 175 °C and then at 4 °C min^−1^ to 230 °C. The final temperature was maintained for 20 min. Compounds were identified by comparing the obtained mass spectra with the NIST-11 library database. Quantification was performed using the area normalization method, with results expressed as the relative percentage of each identified compound.

### 3.5. Analysis of Carotenoids, Phenolic Compounds and Antioxidant Activity

Total carotenoids were determined by measuring the absorbance of the lipophilic extract at 450 nm using a spectrophotometer. The concentration was calculated using the molar extinction coefficient of β-carotene (2592 M/cm) and expressed as μg of β-carotene per gram of sample [65]. Total phenolic compounds (TPCs) and total flavonoid content (TFC) were quantified using the hydrophilic extract. TPC was determined by the Folin–Ciocalteu colorimetric method [66], and TFC was analyzed according to the methodology proposed by Prommuak et al. [67]. Quantification was performed using standard calibration curves of gallic acid for TPC and quercetin for TFC, with results expressed as mg of equivalent per 100 g of dry sample.

The antioxidant activity of lipophilic and hydrophilic extracts was evaluated using three complementary assays. The free radical scavenging capacity of DPPH was analyzed according to Brand-Williams et al. [68]. The antioxidant activity against 2,2′-azino-bis(3-ethylbenzothiazoline-6-sulfonic acid) (ABTS) radical cation was determined following the method described by Re et al. [69]. The ferric reducing antioxidant power (FRAP) test was performed according to Benzie and Strain [70]. Quantification was carried out using a standard curve of Trolox, with results expressed as µmol of Trolox equivalent (TE) per g of sample.

### 3.6. Antimicrobial Activity

The antimicrobial activity of GWO was tested against the pathogens *Staphylococcus aureus* (ATCC 25923), *Bacillus cereus* (ATCC 11778), *Listeria monocytogenes* (ATCC 19117), *Escherichia coli* (ATCC 25922), *Salmonella enterica* subsp. *enterica serovar* Typhimurium (ATCC 1402) and *Candida albicans* (ATCC 10231), using the microdilution method [71]. To determine the minimum inhibitory concentration (MIC), 50 μL of Mueller–Hinton (MH) broth was added to a 96-well plate. A volume of 50 μL of the diluted oils (500 mg mL^−1^) was added in the first column and serial (1:1) dilutions were made. Then, 50 μL of the bacterial suspension in MH broth (about 10^5^ CFU mL^−1^) was added to the well contents. For *C. albicans*, the procedure consisted of adding 100 µL of the diluted oils to 100 µL of Sabouraud broth. After serial dilution, 100 µL of the yeast suspension (about 10^3^ CFU mL^−1^) was added to the wells. Chloramphenicol (32 mg L^−1^) and nystatin (64 mg L^−1^) were used as positive controls. The microplates were incubated at 37 °C for 18–20 h. The MIC was defined as the lowest GWO concentration that inhibited microbial growth, indicated by the addition of resazurin (0.01%). A color change from blue to pink signaled microbial growth [72]. The minimum bactericidal concentration (MBC) was determined as the lowest GWO concentration that resulted in no bacterial growth, confirmed by inoculating an aliquot from the microplate wells onto agar plates.

### 3.7. Antiglycemic Activity

#### 3.7.1. α-Amylase (From Human Saliva) Inhibition Assay

The inhibitory activity of GWO on α-amylase was evaluated as an indicator of its potential antiglycemic properties according to Silva et al. [73]. The α-amylase (EC 3.2.1.1, tipo XIII-A, Sigma-Aldrich, Saint Louis, MO, USA) enzyme solution (74 U mL^−1^) was incubated with GWO at concentrations of 25, 15, 10, and 1 mg mL^−1^, prepared in dimethyl sulfoxide (DMSO). For the inhibition test (IT), 240 µL of phosphate buffer (20 mM containing 6.7 mM NaCl, pH 6.9) was added to test tubes, followed by 25 µL of GWO and 10 µL of the α-amylase solution. The IT solutions were incubated in a water bath at 37 °C for 15 min, and after that time, 250 µL of potato starch solution (1%) was added, followed by another incubation as described above. The reaction was stopped by adding 500 μL of a solution of 3,5-dinitrosalicylic acid (DNS). The tubes were placed in a boiling bath at 100 °C for 5 min and then cooled to room temperature with the addition of 5 mL of distilled water. The reaction was monitored by measuring the absorbance at 550 nm using a UV-VIS spectrophotometer (Shimadzu, UV-1800).

#### 3.7.2. α-Glucosidase (From *Saccharomyces cerevisiae*) Inhibition Assay

The test was performed with an α-glucosidase (EC 3.2.1.20, Sigma-Aldrich, Saint Louis, MO, USA) enzyme solution at 1 U mL^−1^, testing GWO at concentrations of 0.125, 0.250, 0.500, and 1 mg mL^−1^. The experiment was performed according to Souza et al. [74]. Aliquots of 10 μL of GWO, previously diluted in DMSO to the test concentrations, were added to a 96-well microplate, followed by 40 μL of potassium phosphate buffer (0.1 mol L^−1^, pH 6.9) and 100 μL of the α-glucosidase enzyme solution. The plate was incubated at 25 °C for 10 min and then 50 μL of the p-nitrophenyl-α-D-glucopyranoside (p-NPG, Sigma Chemical Co., Saint Louis, MI, USA) substrate solution (1.5 mg mL^−1^ in phosphate buffer) was added. The 1st absorbance reading (0 min) was performed in a microplate reader (TECAN—Infinite M 200, Männedorf, Switzerland) at 405 nm. Then, after 5 min incubation at 25° C, the 2nd reading (5 min) was performed.

### 3.8. Anticancer Activity

GWO was evaluated against the human non-small lung cancer H460 cell line and HEK293 wild-type (WT) cells provided by Dr. Attilio Di Pietro (IBCP, Lyon, France). Cell viability was evaluated by the 3-(4,5-dimethylthiazol-2-yl)-2,5-diphenyltetrazolium bromide (MTT) colorimetric assay [75]. H460 cells were seeded into 96-well culture plates at a density of 3 × 10^4^ and 1.6 × 10^4^ cells/well for 24 h and 72 h treatments, respectively. After overnight incubation, the cells were treated with increasing concentrations of the UAE oil (3.125 to 1600 µg mL^−1^, 2-fold serial dilution) in a volume of 200 μL for 24 and 72 h at 37 °C under 5% CO_2_. After the treatment, 20 μL of a 5 mg mL^−1^ MTT solution was added, and cells were incubated for 4 h at 37 °C under 5% CO2. After incubation, the culture medium was removed, and the formazan crystals were dissolved with 100 µL of DMSO/ethanol (1:1) solution. After obtaining a homogeneous solution, the absorbance was measured at 595 nm using an iMark™ microplate absorbance reader (Bio-Rad, Hercules, CA, USA). The results were expressed as a percentage of viable cells compared to the control condition (0.3% DMSO (*v*/*v*) taken as 100% of viable cells).

### 3.9. Statistical Analysis

All experiments were performed in triplicate and results were expressed as mean followed by standard deviation. Statistical analysis was performed in the software Statistica^®^, version 10.0 (StatSoft, Tulsa, OK, USA), using one-way analysis of variance (ANOVA). The Tukey test was applied to assess differences between means at a significance level of *p* ≤ 0.05.

## 4. Conclusions

Ultrasound-assisted extraction (UAE) proved to be an efficient method for obtaining phytochemical compounds from gabiroba waste with significant biological activities, being a faster and more effective extraction method. The gabiroba waste oil (GWO) presents a variety of compounds, with emphasis on unsaturated fatty acids, sesquiterpenes and carotenoid content, and demonstrates promising antioxidant, antimicrobial, and antihyperglycemic potential for industrial application. Further studies investigating additional cell lines, safety and the toxicological profile of the individual oil components are required to better assess the anticancer potential of GWO. The chemical profile of GWO highlights its potential for natural product formulations aimed at combating oxidative stress, microbial infections, and supporting glycemic control. The present study provides data consistent with sustainable practices, contributing to the reduction in the ecological impact of industrial waste by employing them in extraction processes, significantly reducing extraction time and energy consumption by employing the UAE technique.

## Figures and Tables

**Table 1:** Chemical composition of gabiroba waste oil obtained by Soxhlet (SE) and ultrasound-assisted extraction (UAE).

Compound	SE	UAE
**Fatty Acids**		
Myristic acid (C14:0)	0.59	0.28
Palmitoleic acid (C16:1)	3.78	3.24
Palmitic acid (C16:0)	28.28	18.05
Linoleic acid (C18:2)	5.61	5.25
Oleic acid (C18:1)	35.01	46.36
Elaidic acid (C18:1)	2.97	2.42
Stearic acid (C18:0)	2.25	1.68
Eicosanoic acid (C20:0)	0.19	-
**Sesquiterpenes**		
β-Cadinene	4.36	2.53
β-Patchoulene	0.18	0.16
γ-Gurjunene	0.38	-
Isoledene	1.45	2.55
α-Copaene	0.12	1.58
β-Maliene	0.46	1.75
Valencene	0.42	-
γ-Selinene	1.84	1.23
α-Neoclovene	-	0.3
α-Murolene	1.85	0.28
γ-Maliene	0.18	-
α-Gurjunene	0.11	-
Cis-Calamenene	0.51	0.36
γ-Murolene	0.43	-
Selina-3,7(11)-diene	0.12	0.37
Guaia-3,9-diene	-	0.25
δ-Cadinene	1.12	1.31
β-Gurjunene	0.40	-
Ambrox	0.48	0.49
Clovene	2.32	-
Aristolene	0.27	0.27
α-Cadinene	0.62	-
Eudesmol	0.20	-
γ-Eudesmol	-	0.53
Guaiol	-	0.16
Aristolochene	-	0.18
Pinocembrin	-	-
Fatty acids (%)	78.68	77.28
Saturated	31.31	20.01
Unsaturated	47.37	57.27
Sesquiterpenes (%)	18.26	14.3
Total of identified compounds (%)	96.94	91.58

SE, Soxhlet extraction; UAE, ultrasound-assisted extraction. Compounds were quantified by the area normalization procedure and the results were expressed as a percentage of each compound identified in the sample.

**Table 2:** Physicochemical parameters of gabiroba waste oil.

Parameters	UAE	SE
Extraction yield (%)	36.6 ± 0.8 ^a^	34.7 ± 0.5 ^b^
Acidity index (%)	12.5 ± 0.2 ^b^	14.1 ± 0.5 ^a^
Density (Kg m^−3^)	922.8 ± 0.0	932.4 ± 0.0
Refraction index	1.4802 ^b^ ± 0.0006	1.4894 ^a^ ± 0.0001

UAE, ultrasound-assisted extraction; SE, Soxhlet extraction. Means followed by equal letters in the same line do not differ by Tukey’s test (*p* > 0.05).

**Table 3:** Antimicrobial activity of gabiroba waste oil using microdilution method.

Microorganisms Tested	Positive Control	SE		UAE	
	MBC (mg L^−1^)	MIC	MBC	MIC	MBC
*S. aureus*	0.250	62.50	250.00	3.90	31.25
*B. cereus*	0.125	0.12	0.12	0.24	0.49
*L. monocytogenes*	0.500	ND	ND	ND	ND
*E. coli*	<0.032	ND	ND	ND	ND
*S. enterica*	0.032	250.00	ND	250	ND
*C. albicans*	1.000	15.63	62.5	15.63	31.25

Note: results for GWO samples are expressed in mg mL^−1^, while results for the positive control are expressed in mg L^−1^. ND, not detected; MBC, minimum bactericidal concentration; MIC, minimum inhibitory concentration; SE, Soxhlet extraction; UAE, ultrasound-assisted extraction.

## Data Availability

The original contributions presented in the study are included in the article; further inquiries can be directed to the corresponding authors.

## Supplementary Materials

The following supporting information can be downloaded at: https://www.mdpi.com/article/10.3390/molecules31203705/s1.
