# Supplementary material for: Assessing the Potential of Gabiroba Fruit Waste as an Oil Source via Ultrasound-Assisted Extraction: Physicochemical Parameters, Chemical Profile, and Biological Properties
Source: Molecules. 2026 Oct 16;31(20):3705. doi: 10.3390/molecules31203705 (PMC13707681; doi:10.3390/molecules31203705)

Supplementary Material

Assessing the Potential of Gabiroba Fruit Waste as an Oil Source via Ultrasound-Assisted Extraction: Physicochemical Parameters, Chemical Profile, and Biological Properties

Carolina Lilibeth Carvalho de Pinho [1], Renata Lázara de Araújo [2,*], Suelen Ávila [3], Giselle Maria Maciel [4], Isadora da Silva Zanzarini [5], Vivian Rotuno Moure [5], Glaucio Valdameri [5], Fabiane Oliveira Farias [1], Cristiane Vieira Helm [6], Marcos Rogério Mafra [1] and Luciana Igarashi-Mafra [1]

[1] Department of Chemical Engineering, Postgraduate Program in Food Engineering, Federal University of Paraná, Curitiba 81531-980, PR, Brazil; carolinacpinho@gmail.com (C.L.C.d.P.); fabianefarias@ufpr.br (F.O.F.); marcos.mafra@ufpr.br (M.R.M.); luciana.igarashi@gmail.com (L.I.-M.)
[2] Institute of Exact and Earth Sciences, School of Food Engineering, Federal University of Mato Grosso, Barra do Garças 78605-091, MT, Brazil
[3] Postgraduate Program in Food and Nutrition, Health Science Sector, Federal University of Paraná, Campus III, Curitiba 80210-170, PR, Brazil; suelenavila@gmail.com
[4] Biotechnology Laboratory, Department of Chemistry and Biology, Federal University of Technology—Paraná (UTFPR), Curitiba 81280-340, PR, Brazil; gisellemariam@gmail.com
[5] Postgraduate Program in Pharmaceutical Sciences, Laboratory of Cancer Drug Resistance, Federal University of Parana, Curitiba 80210-170, PR, Brazil; isadorazanzarini@ufpr.br (I.d.S.Z.); vivian.moure@ufpr.br (V.R.M.); gvaldameri@ufpr.br (G.V.)
[6] Brazilian Agricultural Research Corporation (Embrapa Florestas), Colombo 83411-000, PR, Brazil; cristiane.helm@embrapa.br
* Correspondence: renata.araujo4@ufmt.br

Figure S1. Total-ion-current (TIC) chromatograms of gabiroba waste oil obtained by ultrasound-assisted extraction (UAE).

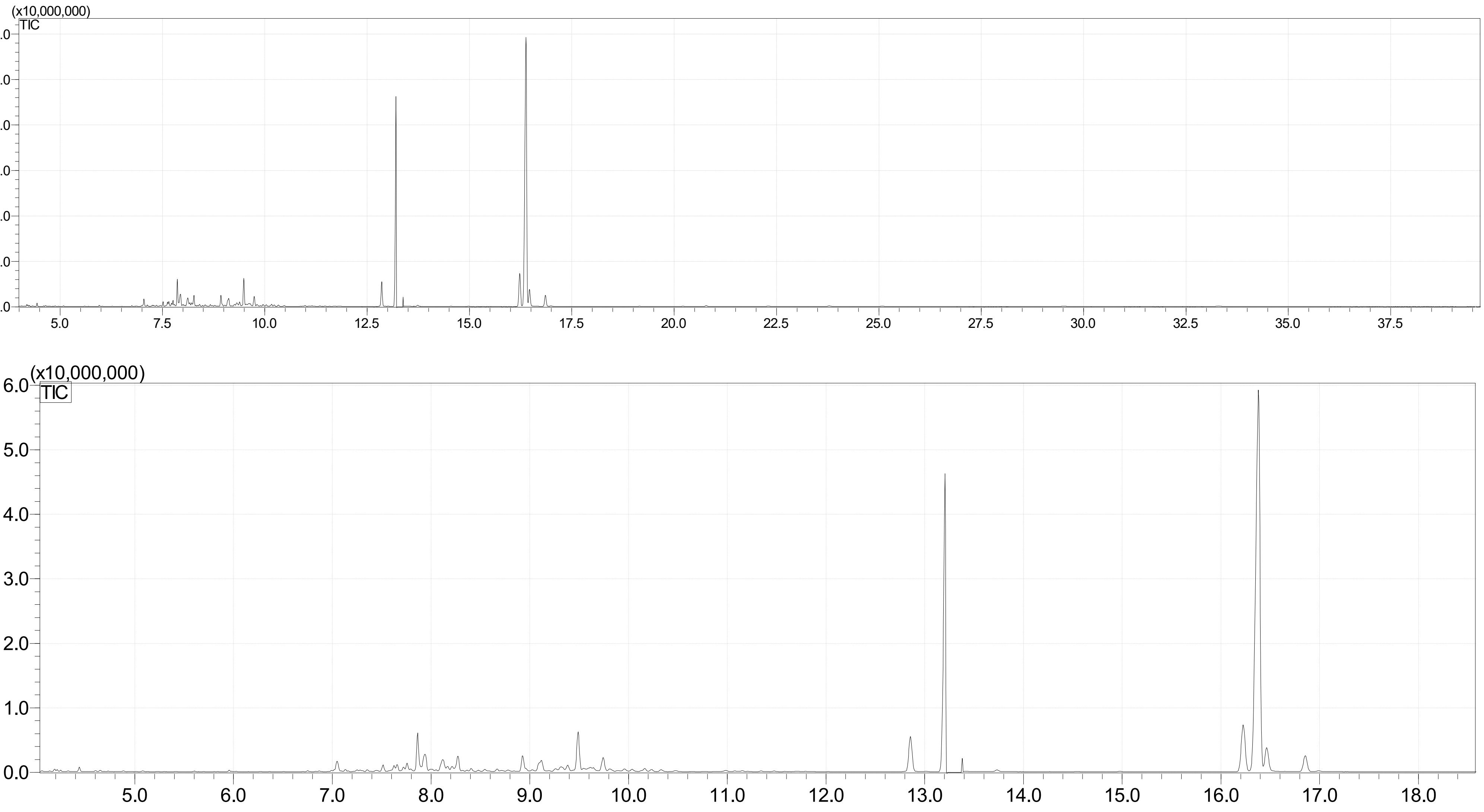

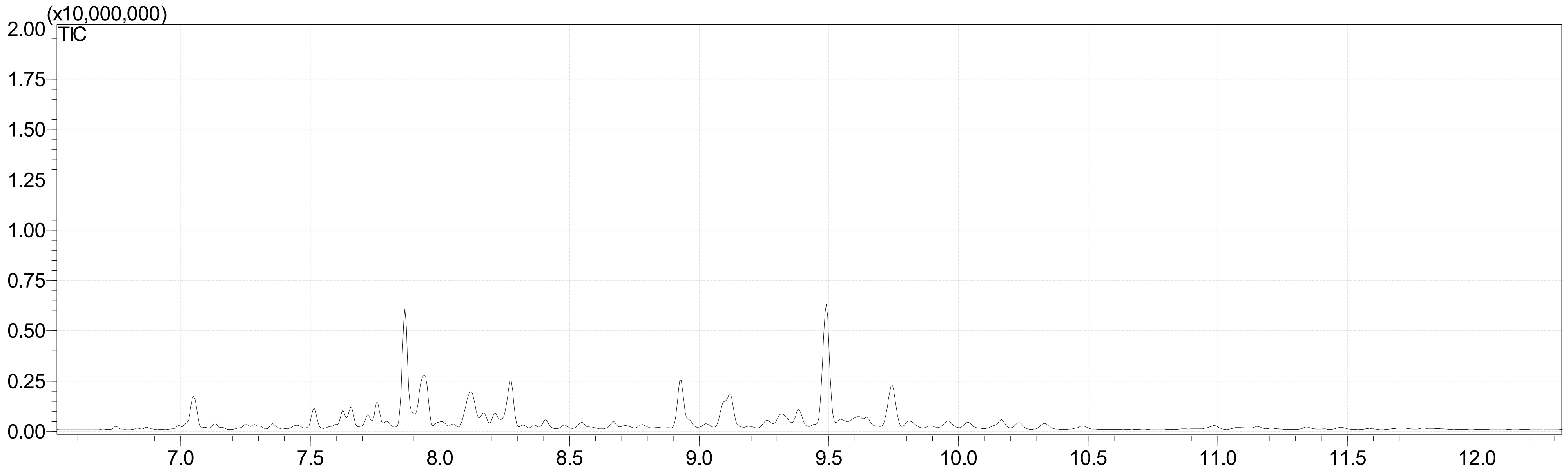
(x10,000,000)
TIC
2.00
1.75
1.50
1.25
1.00
0.75
0.50
0.25
0.00
7.0
7.5
8.0
8.5
9.0
9.5
10.0
10.5
11.0
11.5
12.0

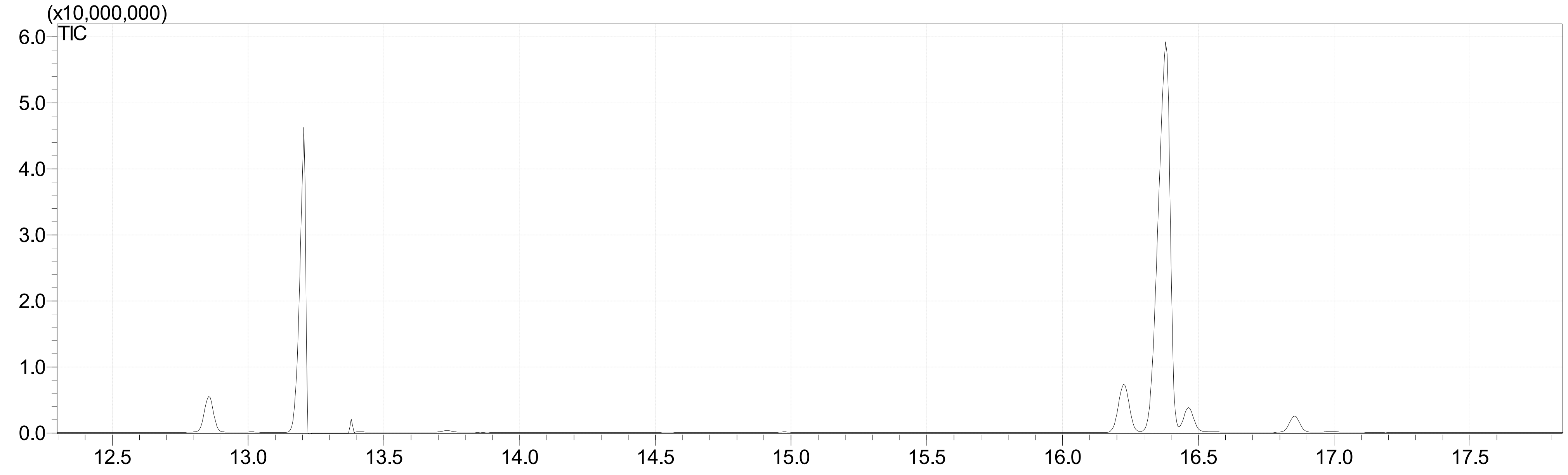
(x10,000,000)
TIC
6.0
5.0
4.0
3.0
2.0
1.0
0.0
12.5
13.0
13.5
14.0
14.5
15.0
15.5
16.0
16.5
17.0
17.5

Figure S2. Experimental mass spectra of the major compounds identified in gabiroba waste oil obtained by ultrasound-assisted extraction (UAE).

γ-Selinene

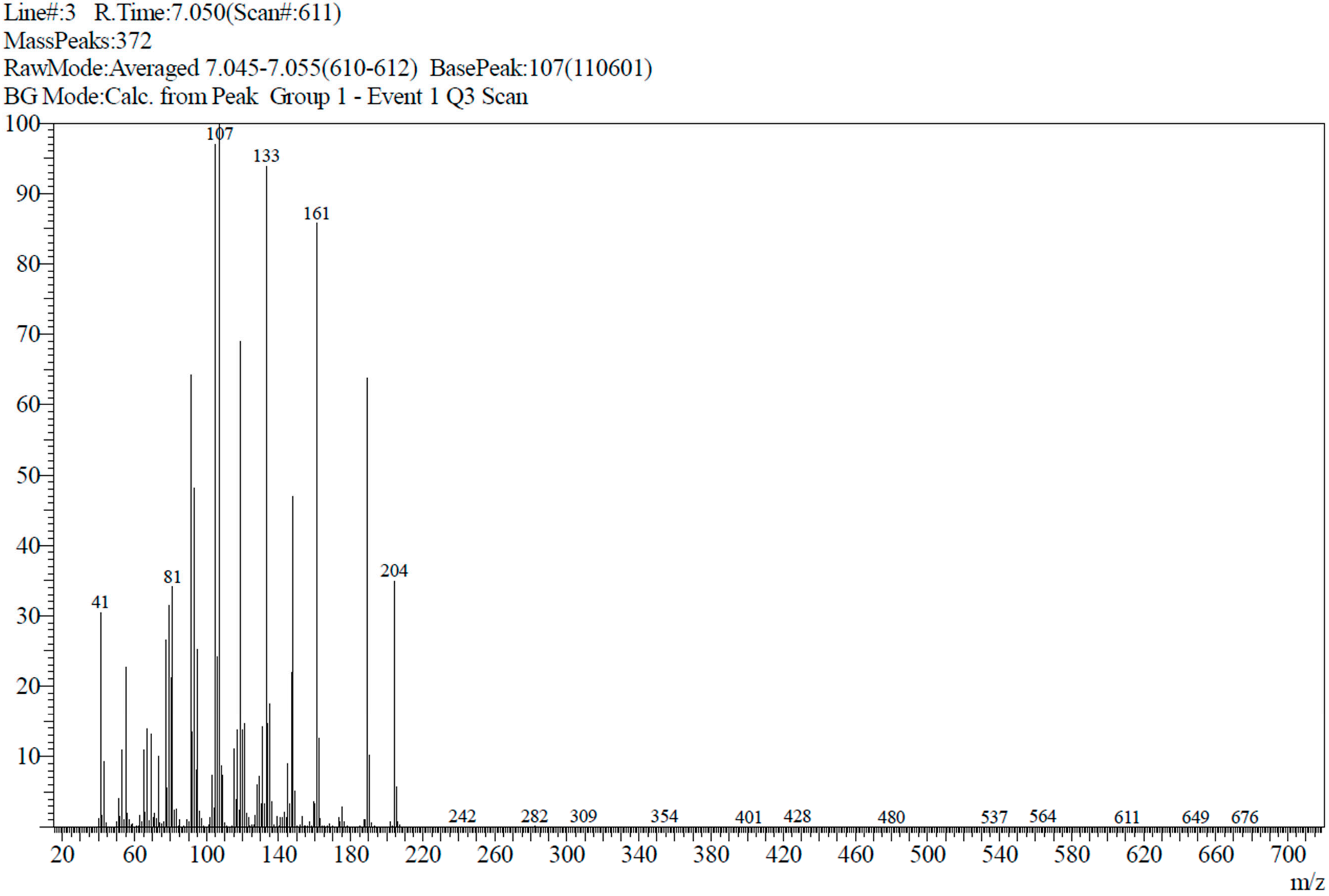

β-Patchoulene

Line#:4   R.Time:7.130(Scan#:627)
MassPeaks:342
RawMode:Averaged 7.125-7.135(626-628) BasePeak:161(26813)
BG Mode:Calc. from Peak Group 1 - Event 1 Q3 Scan

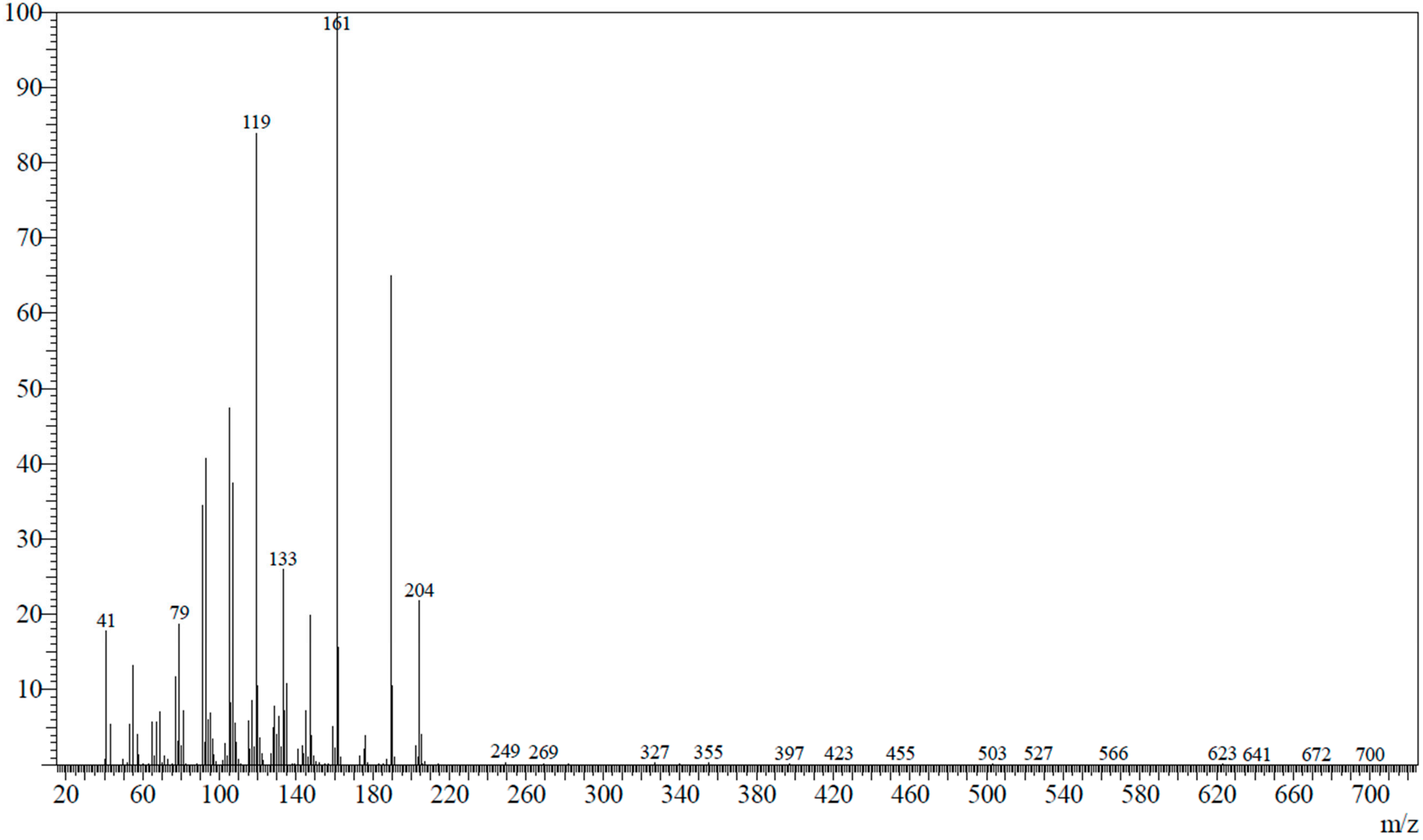

β-Maliene

Line#:8 R.Time:7.625(Scan#:726)
MassPeaks:379
RawMode:Averaged 7.620-7.630(725-727) BasePeak:105(43053)
BG Mode:Calc. from Peak Group 1 - Event 1 Q3 Scan

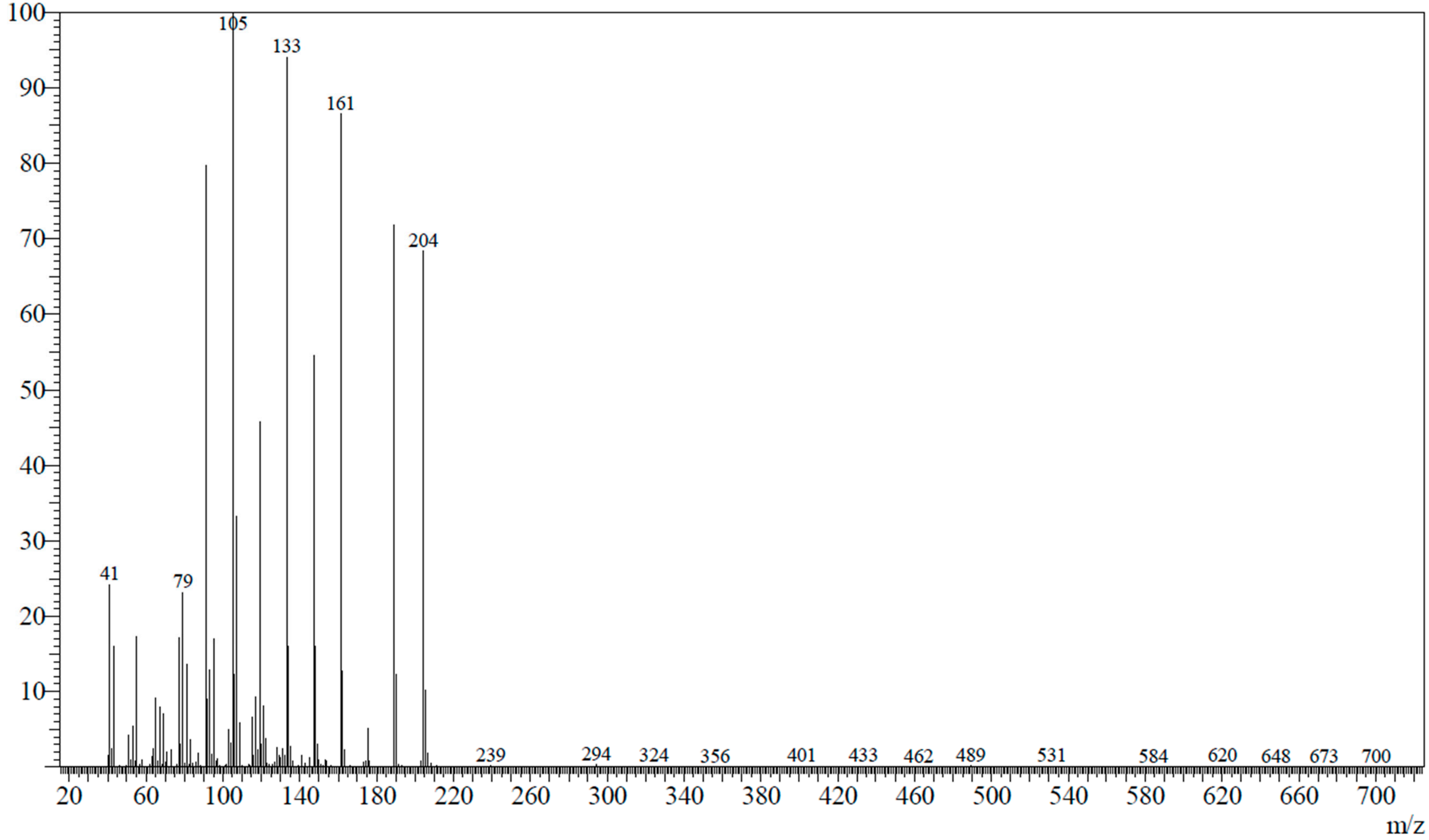

α-Neoclovene

Line#:10 R.Time:7.720(Scan#:745)

MassPeaks:383

RawMode:Averaged 7.715-7.725(744-746) BasePeak:161(31895)

BG Mode:Calc. from Peak Group 1 - Event 1 Q3 Scan

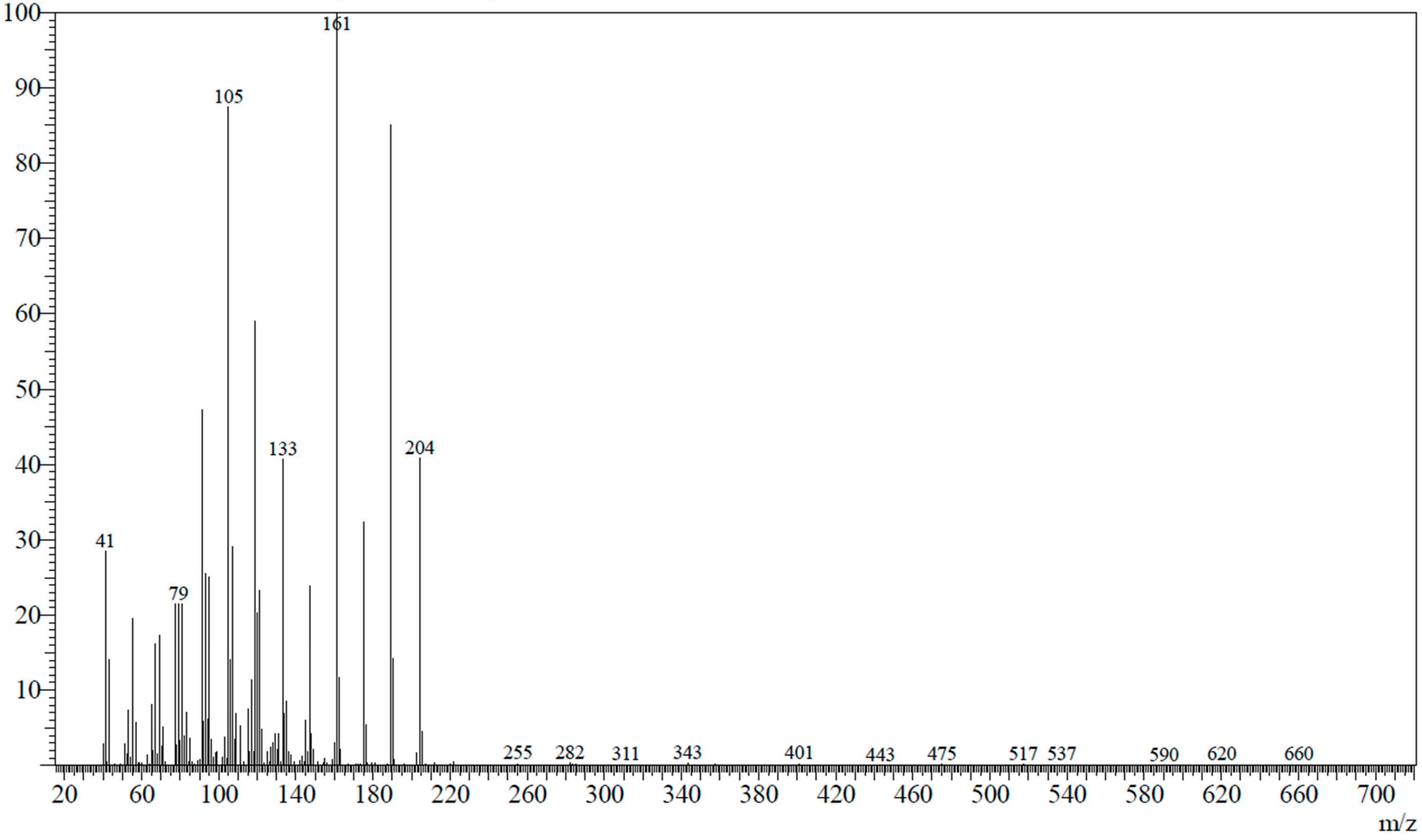

α-Murolene

Line#:12 R.Time:7.795(Scan#:760)
MassPeaks:374
RawMode:Averaged 7.790-7.800(759-761) BasePeak:105(29333)
BG Mode:Calc. from Peak Group 1 - Event 1 Q3 Scan

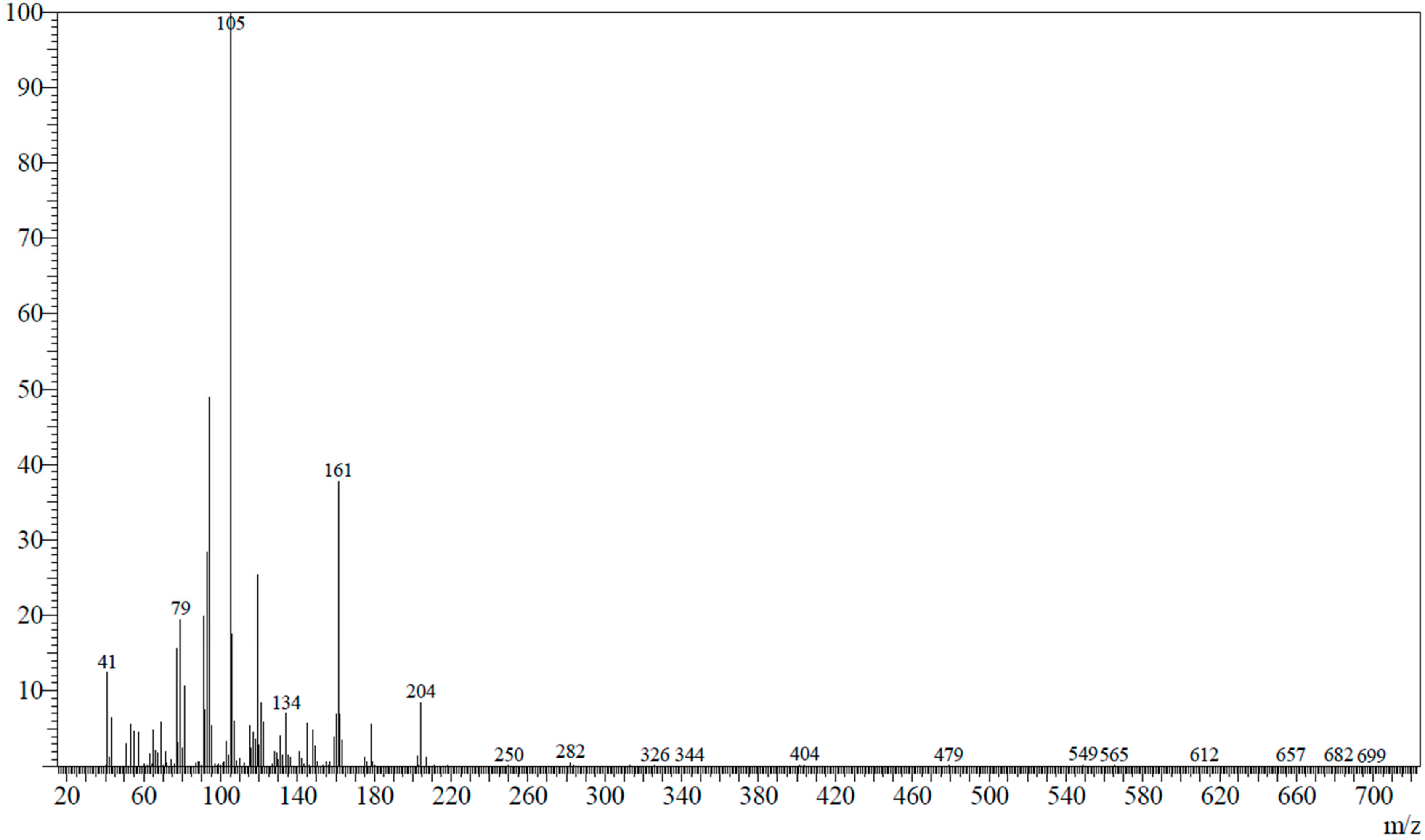

β-Cadinene

Line#:13 R.Time:7.865(Scan#:774)
MassPeaks:404
RawMode:Averaged 7.860-7.870(773-775) BasePeak:161(587457)
BG Mode:Calc. from Peak Group 1 - Event 1 Q3 Scan

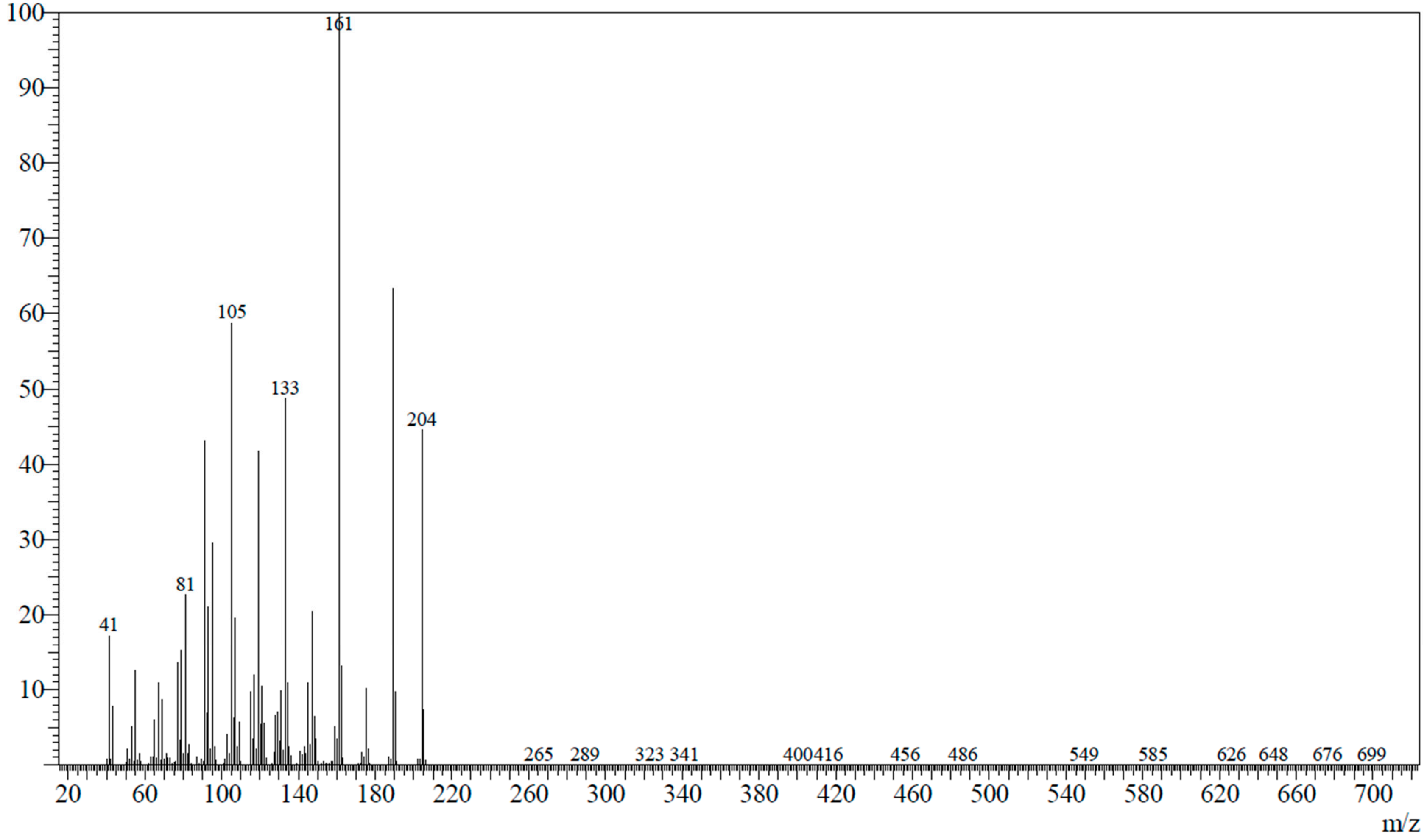

Isoledene

Line#:14 R.Time:7.940(Scan#:789)
MassPeaks:372
RawMode:Averaged 7.935-7.945(788-790) BasePeak:105(260182)
BG Mode:Calc. from Peak Group 1 - Event 1 Q3 Scan

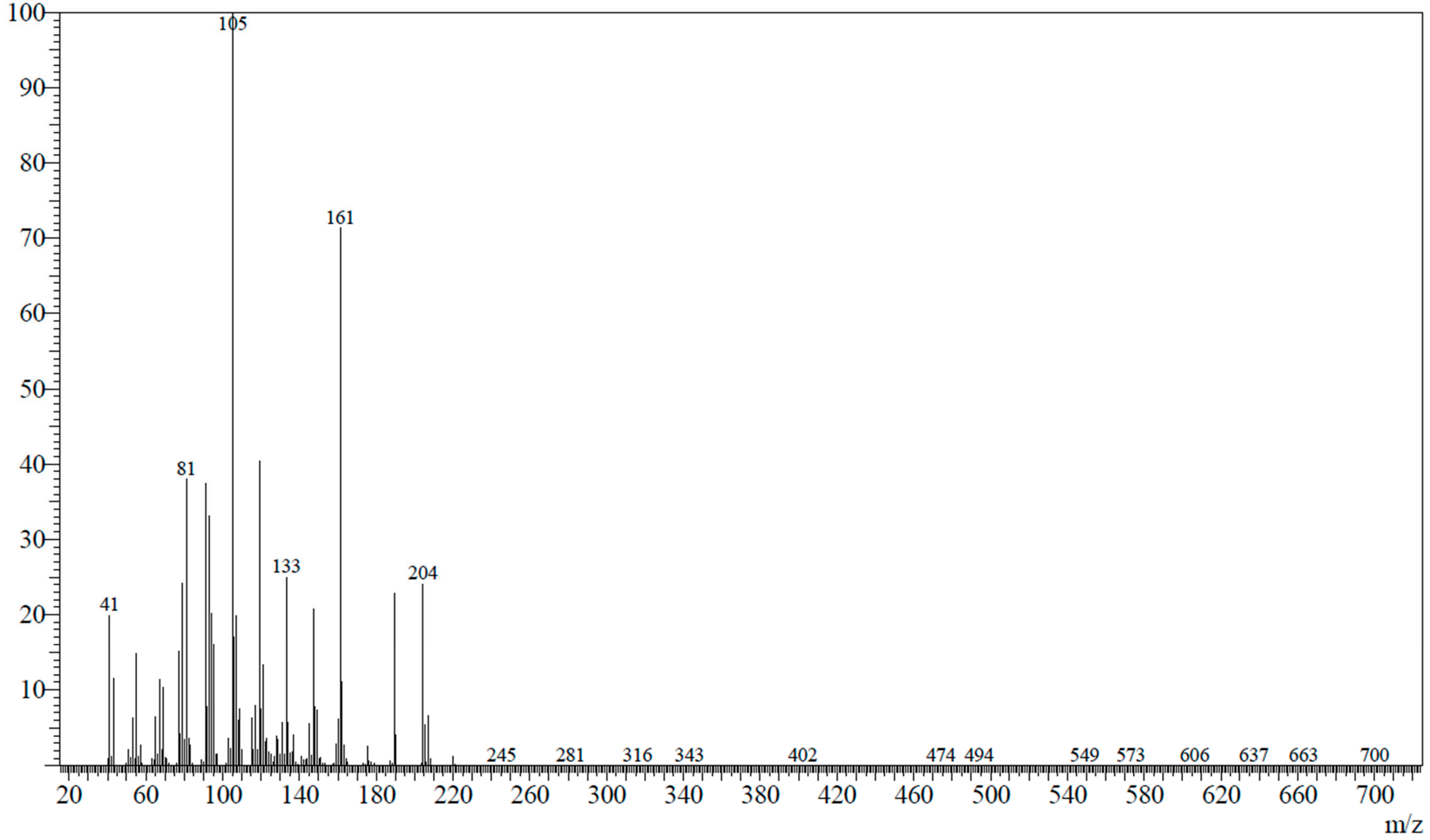

Guaia-3,9-diene

Line#:15 R.Time:8.010(Scan#:803)
MassPeaks:327
RawMode:Averaged 8.005-8.015(802-804) BasePeak:161(14413)
BG Mode:Calc. from Peak Group 1 - Event 1 Q3 Scan

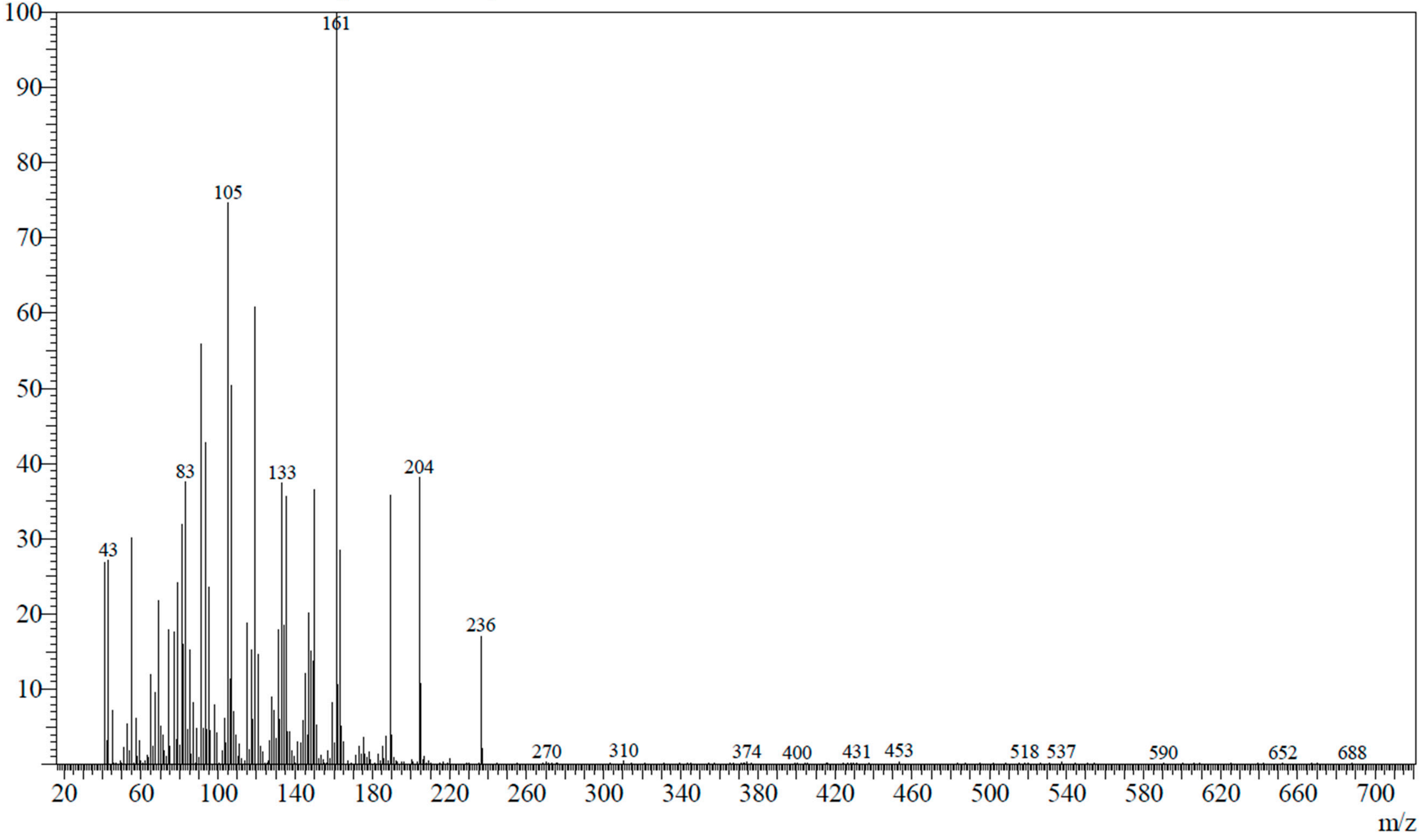

α-Copaene

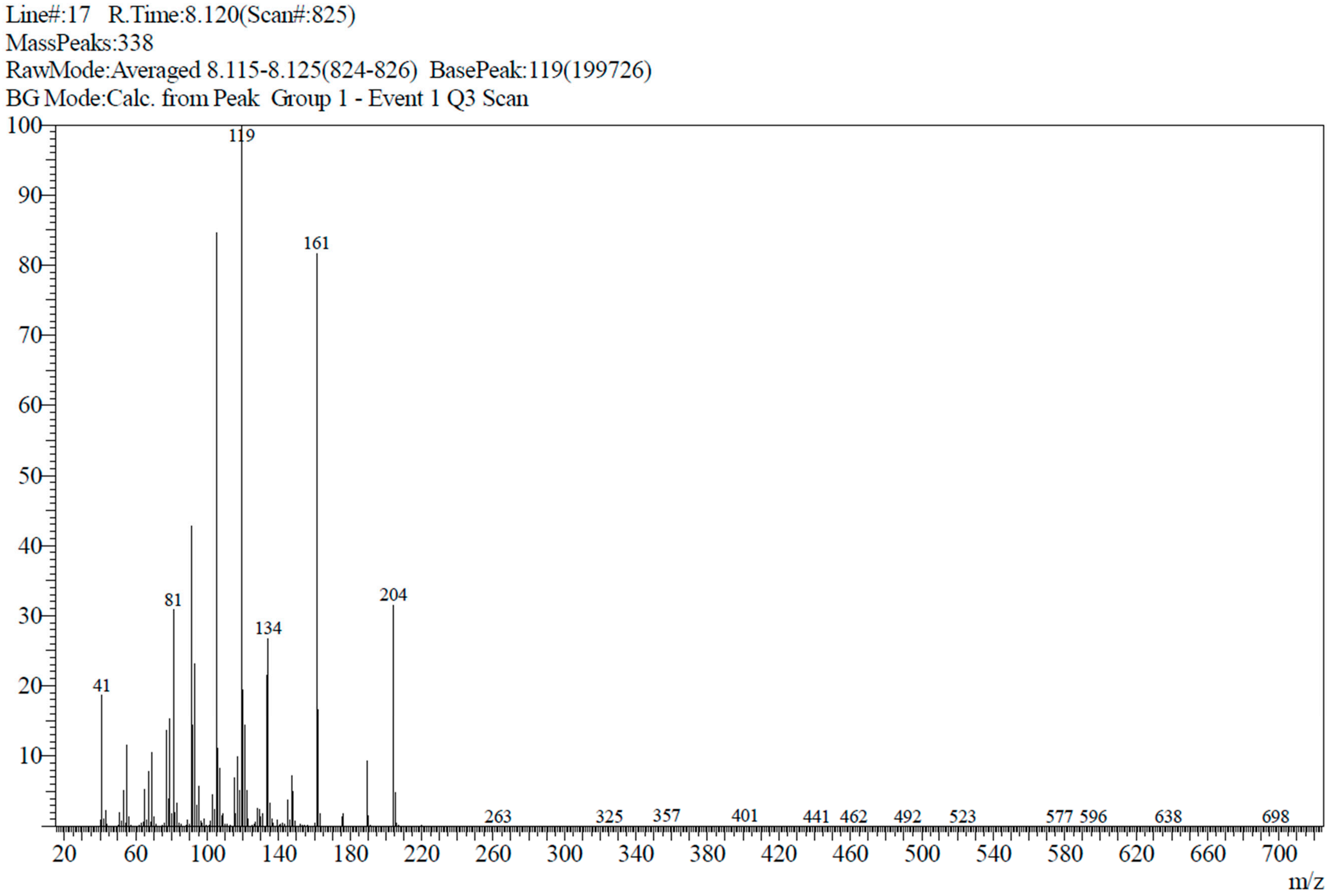

Line#:17 R.Time:8.120(Scan#:825)
MassPeaks:338
RawMode:Averaged 8.115-8.125(824-826) BasePeak:119(199726)
BG Mode:Calc. from Peak Group 1 - Event 1 Q3 Scan
100
90
80
70
60
50
40
30
20
10
119
161
204
81
134
41
263
325
357
401
441
462
492
523
577
596
638
698
20
60
100
140
180
220
260
300
340
380
420
460
500
540
580
620
660
700
m/z

Cis-Calamenene

Line#:18 R.Time:8.170(Scan#:835)
MassPeaks:274
RawMode:Averaged 8.165-8.175(834-836) BasePeak:159(179202)
BG Mode:Calc. from Peak Group 1 - Event 1 Q3 Scan

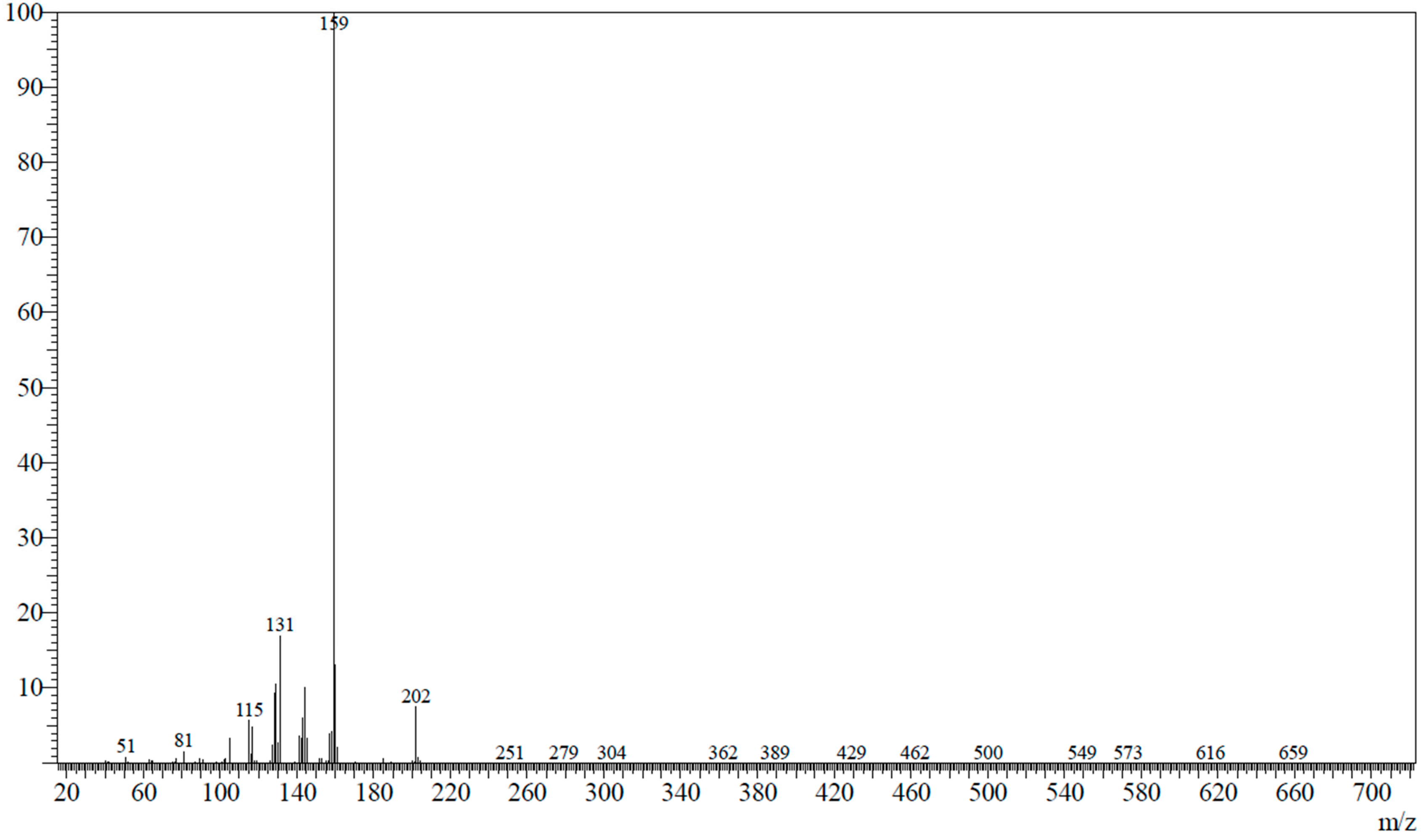

Guaiol

Line#:22 R.Time:8.545(Scan#:910)
MassPeaks:345
RawMode:Averaged 8.540-8.550(909-911) BasePeak:73(34971)
BG Mode:Calc. from Peak Group 1 - Event 1 Q3 Scan

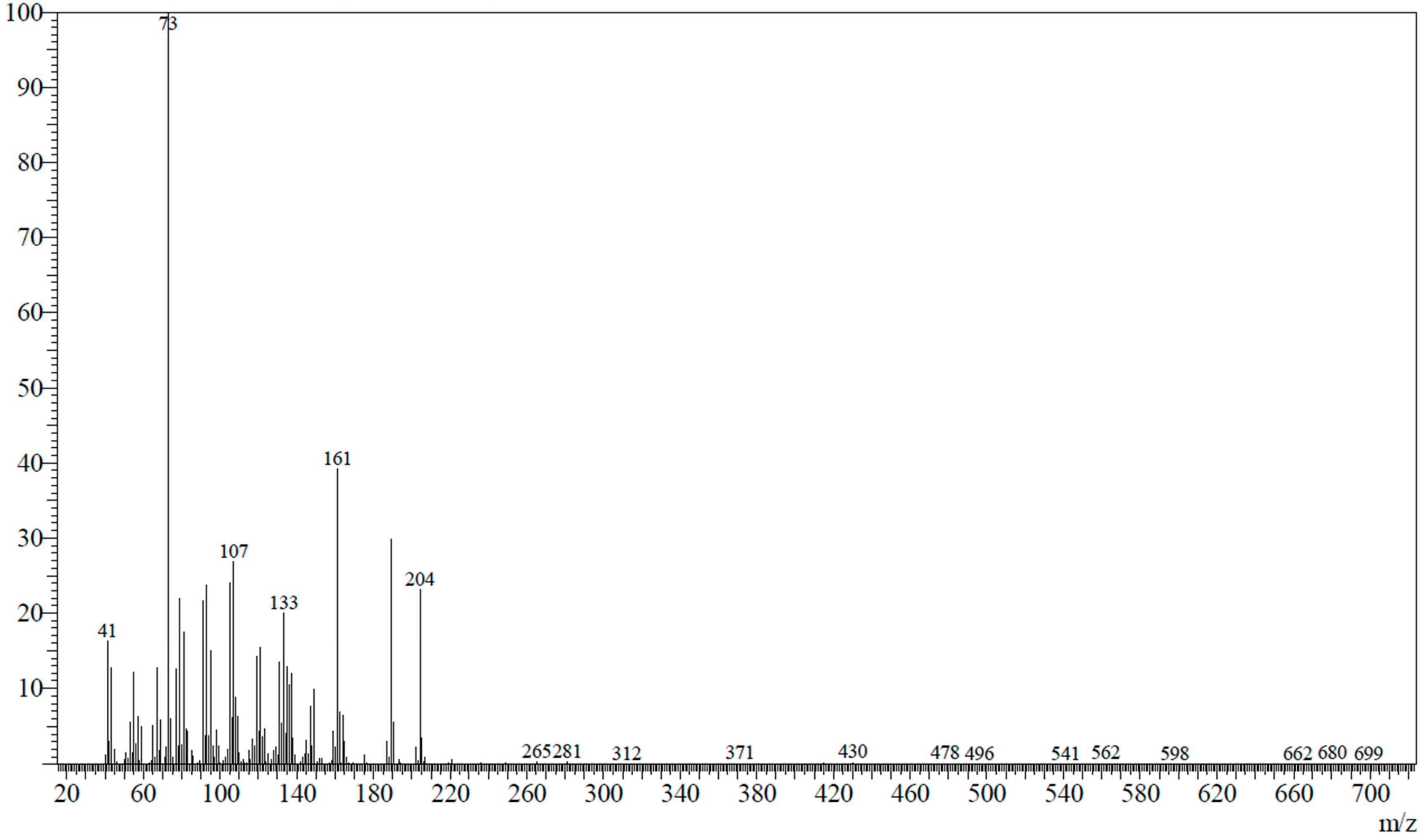

δ-Cadinene

Line#:27 R.Time:9.120(Scan#:1025)
MassPeaks:401
RawMode:Averaged 9.115-9.125(1024-1026) BasePeak:161(225500)
BG Mode:Calc. from Peak Group 1 - Event 1 Q3 Scan

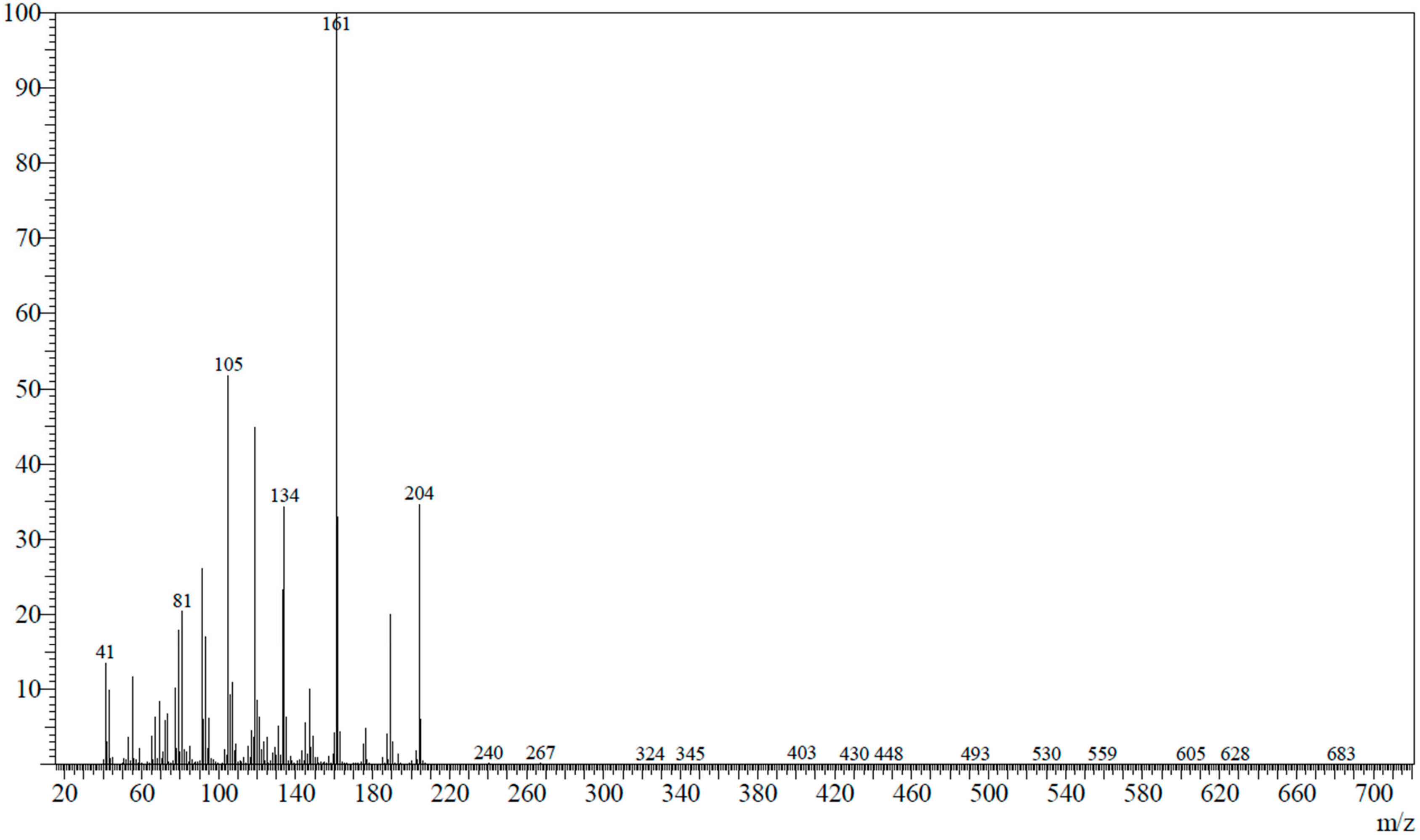

γ-Eudesmol

Line#:29   R.Time:9.315(Scan#:1064)
MassPeaks:407
RawMode:Averaged 9.310-9.320(1063-1065) BasePeak:161(54328)
BG Mode:Calc. from Peak Group 1 - Event 1 Q3 Scan

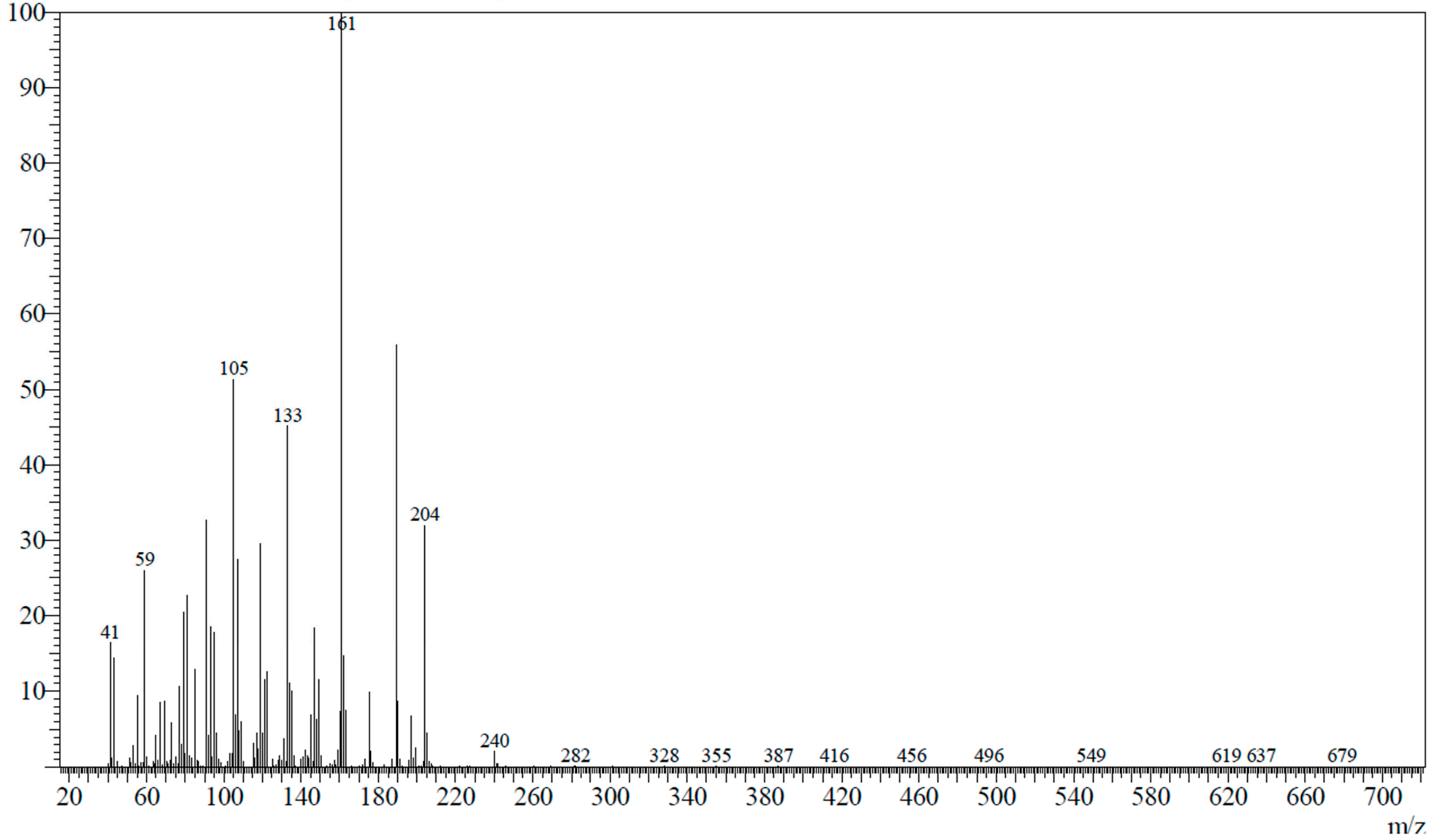

Ambrox

Line#:30   R.Time:9.385(Scan#:1078)

MassPeaks:385

RawMode:Averaged 9.380-9.390(1077-1079) BasePeak:221(164180)

BG Mode:Calc. from Peak Group 1 - Event 1 Q3 Scan

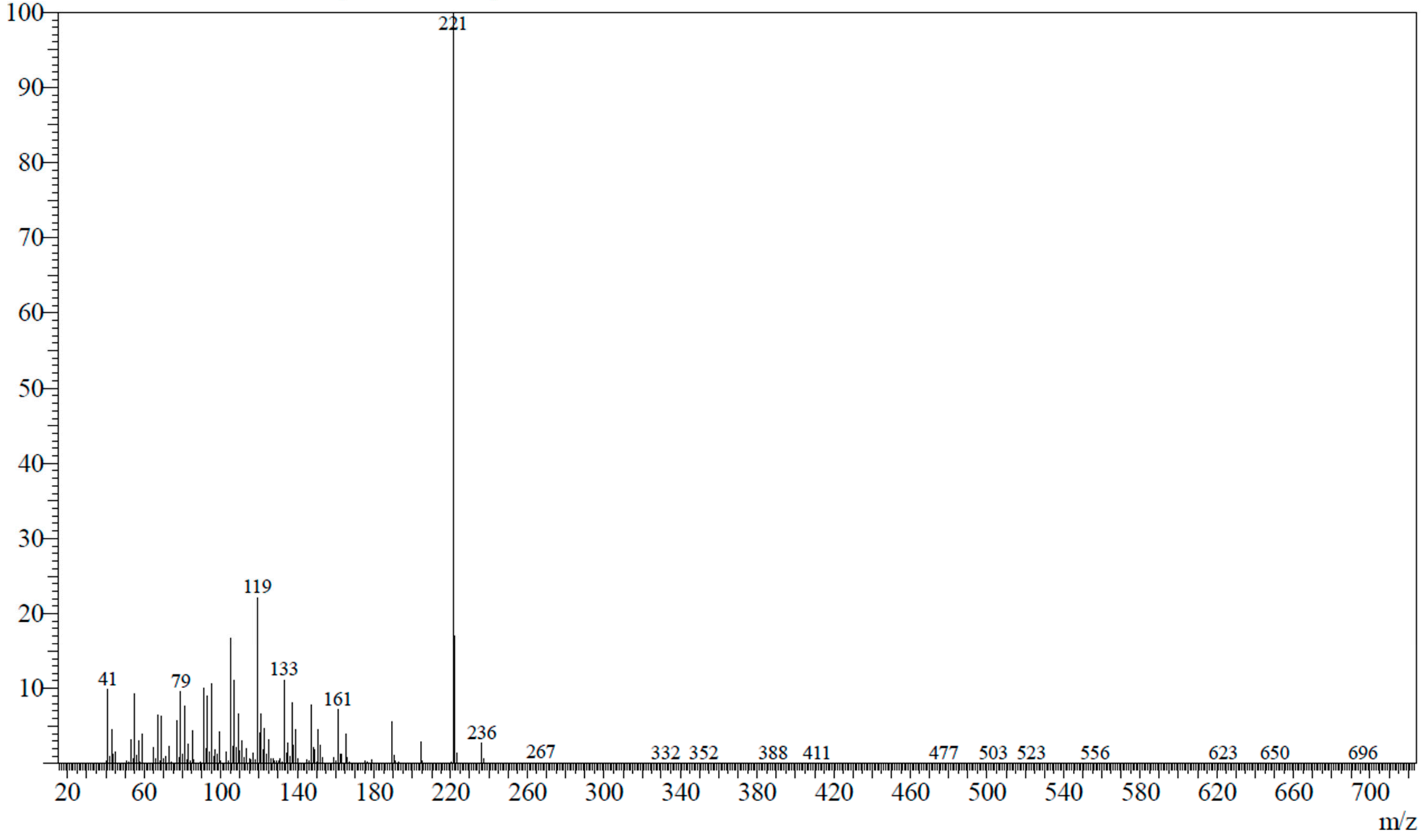

Aristolene

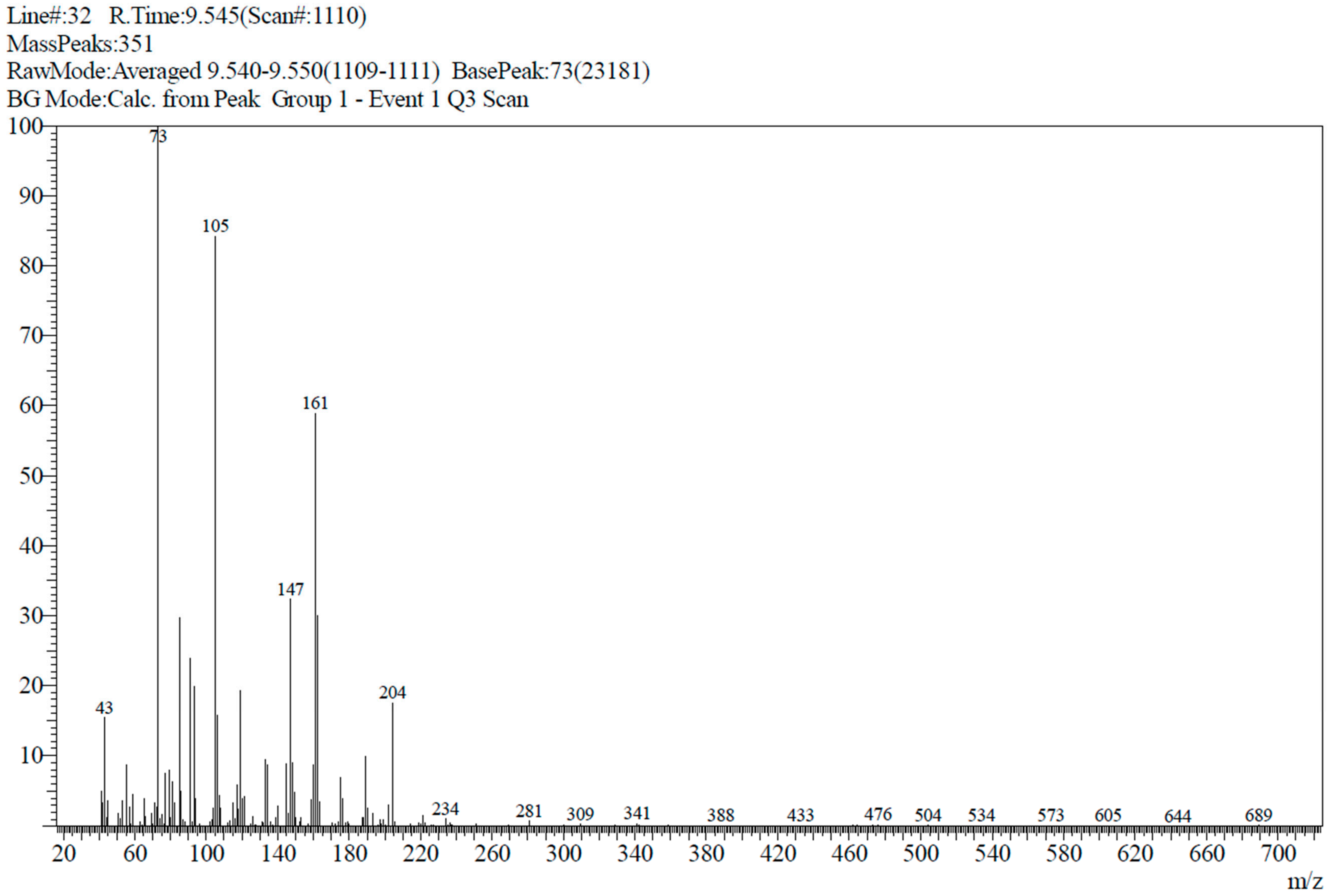

Line#:32 R.Time:9.545(Scan#:1110)
MassPeaks:351
RawMode:Averaged 9.540-9.550(1109-1111) BasePeak:73(23181)
BG Mode:Calc. from Peak Group 1 - Event 1 Q3 Scan
100
90
80
70
60
50
40
30
20
10
73
105
161
147
204
43
234
281
309
341
388
433
476
504
534
573
605
644
689
20
60
100
140
180
220
260
300
340
380
420
460
500
540
580
620
660
700
m/z

Aristolochene

Line#:38   R.Time:10.035(Scan#:1208)

MassPeaks:375

RawMode:Averaged 10.030-10.040(1207-1209) BasePeak:105(29754)

BG Mode:Calc. from Peak Group 1 - Event 1 Q3 Scan

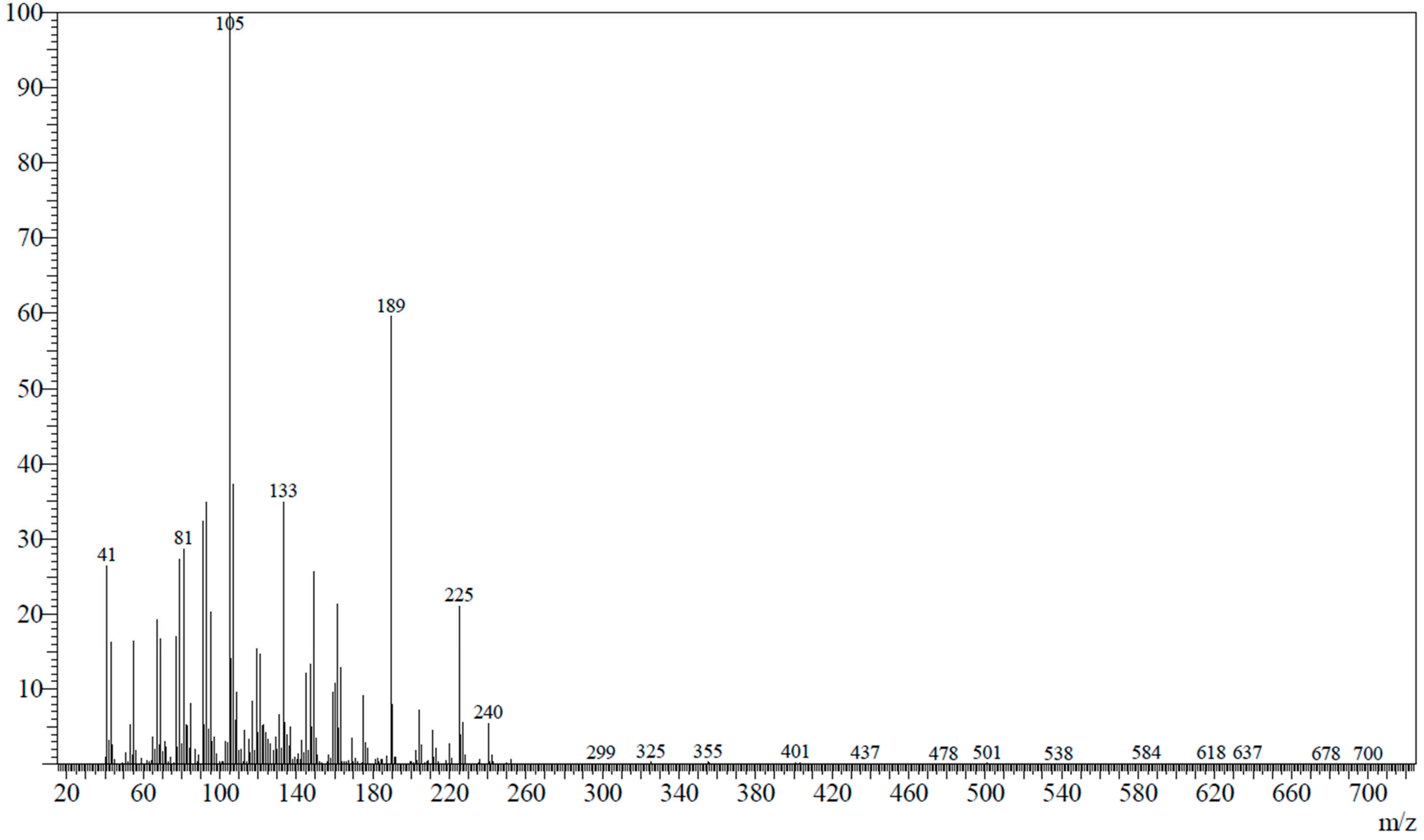

Myristic acid

Line#:39 R.Time:10.165(Scan#:1234)
MassPeaks:390
RawMode:Averaged 10.160-10.170(1233-1235) BasePeak:74(94790)
BG Mode:Calc. from Peak Group 1 - Event 1 Q3 Scan

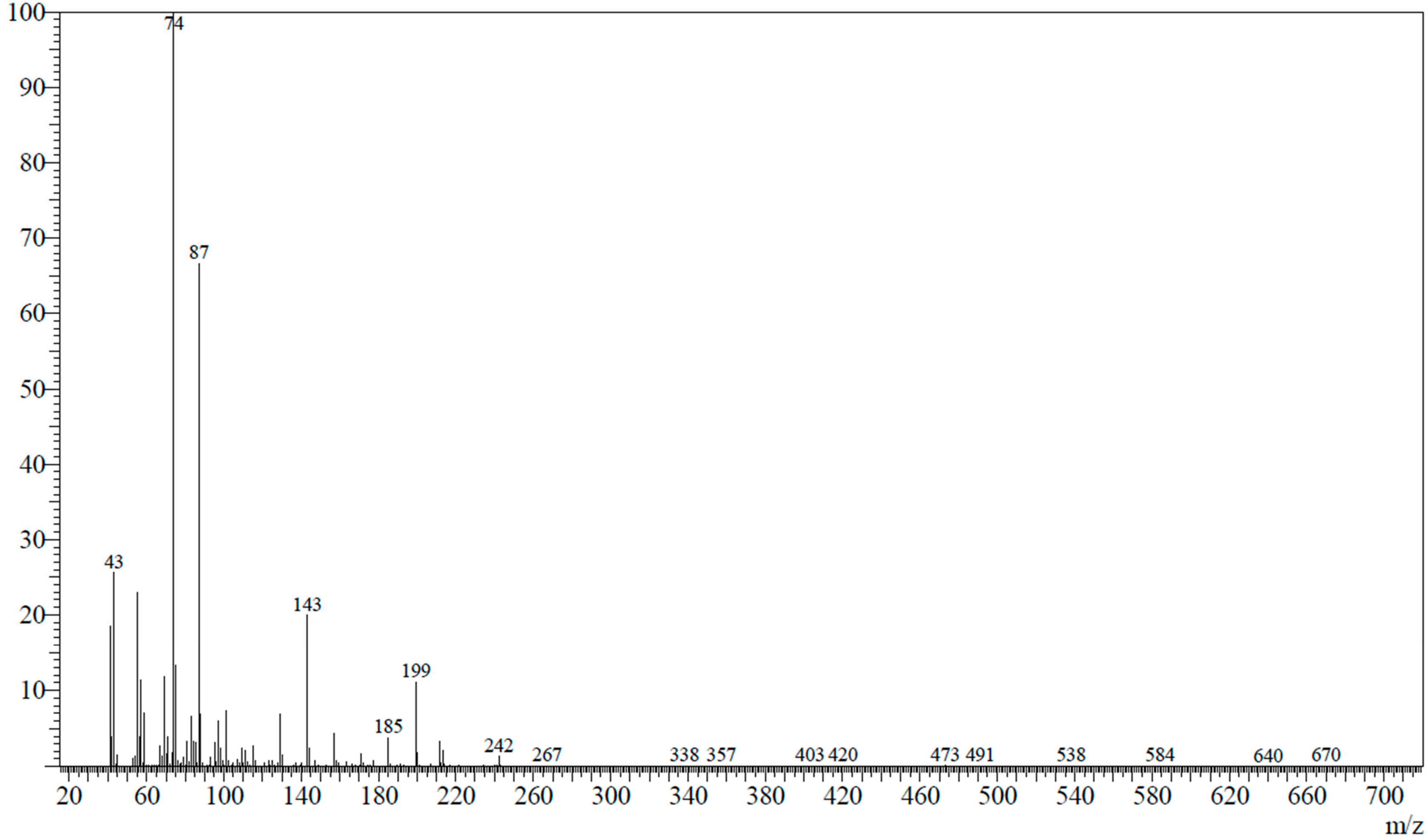

Palmitoleic acid

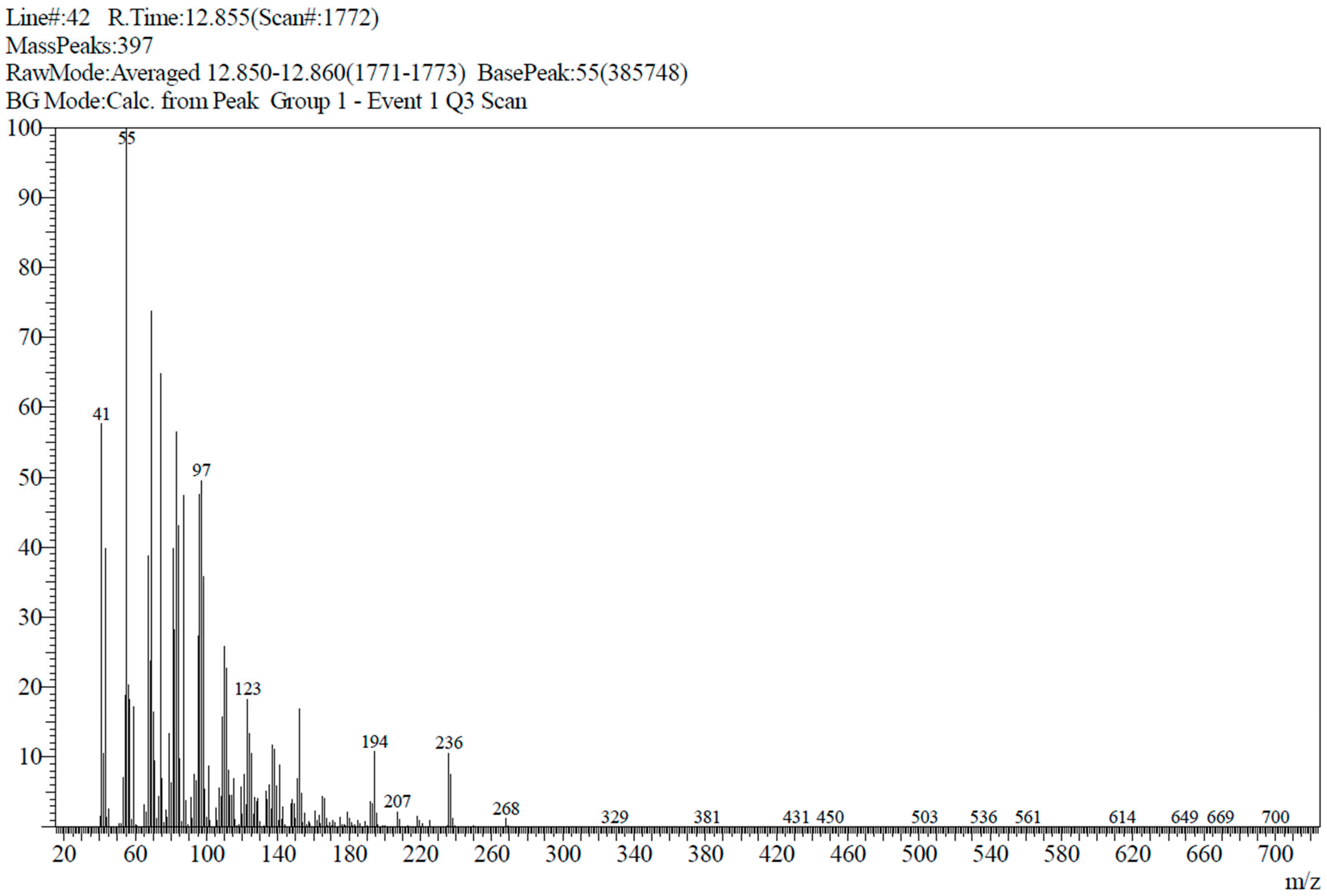

Palmitic acid

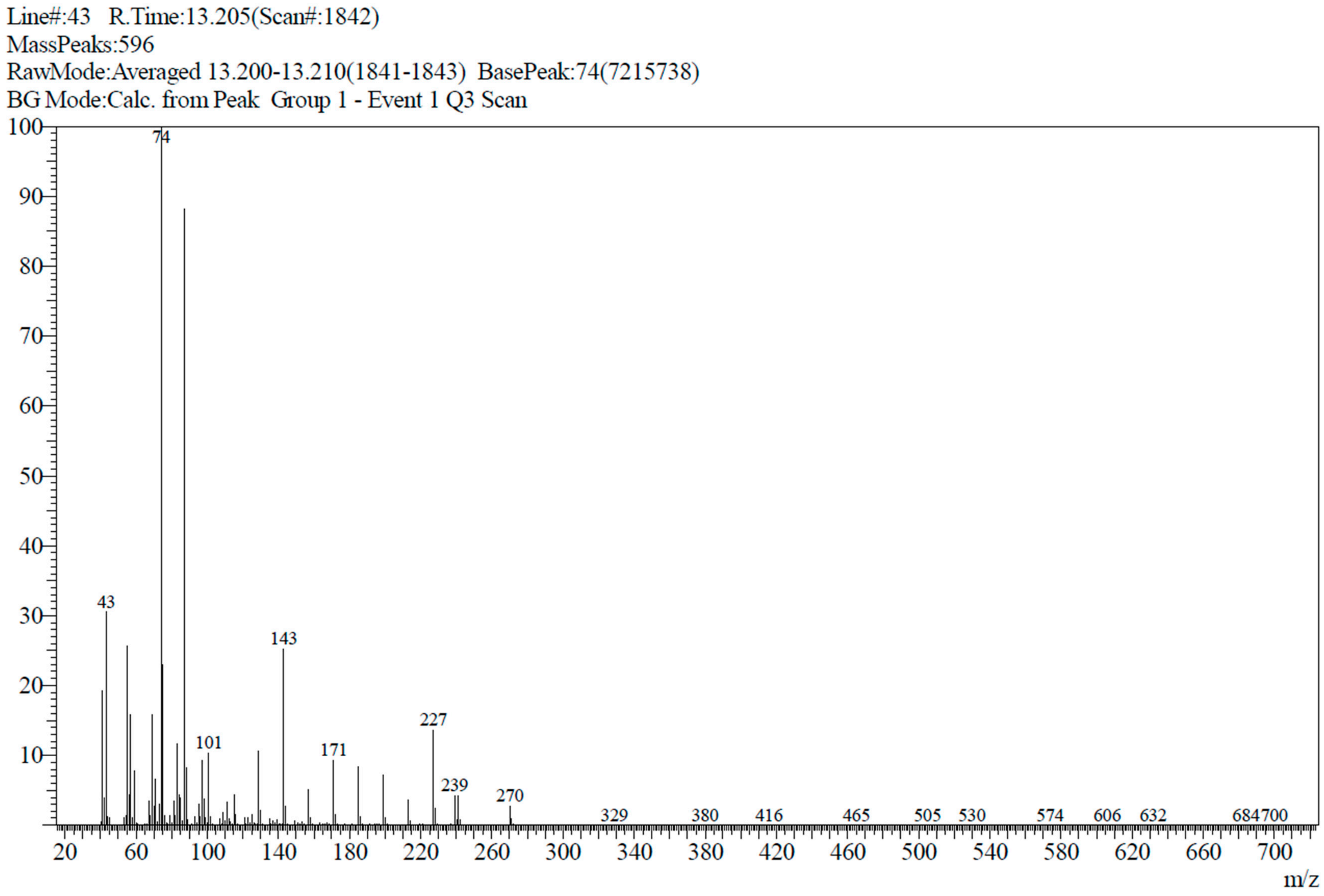

Linoleic acid

Line#:47 R.Time:16.225(Scan#:2446)
MassPeaks:462
RawMode:Averaged 16.220-16.230(2445-2447) BasePeak:67(633974)
BG Mode:Calc. from Peak Group 1 - Event 1 Q3 Scan

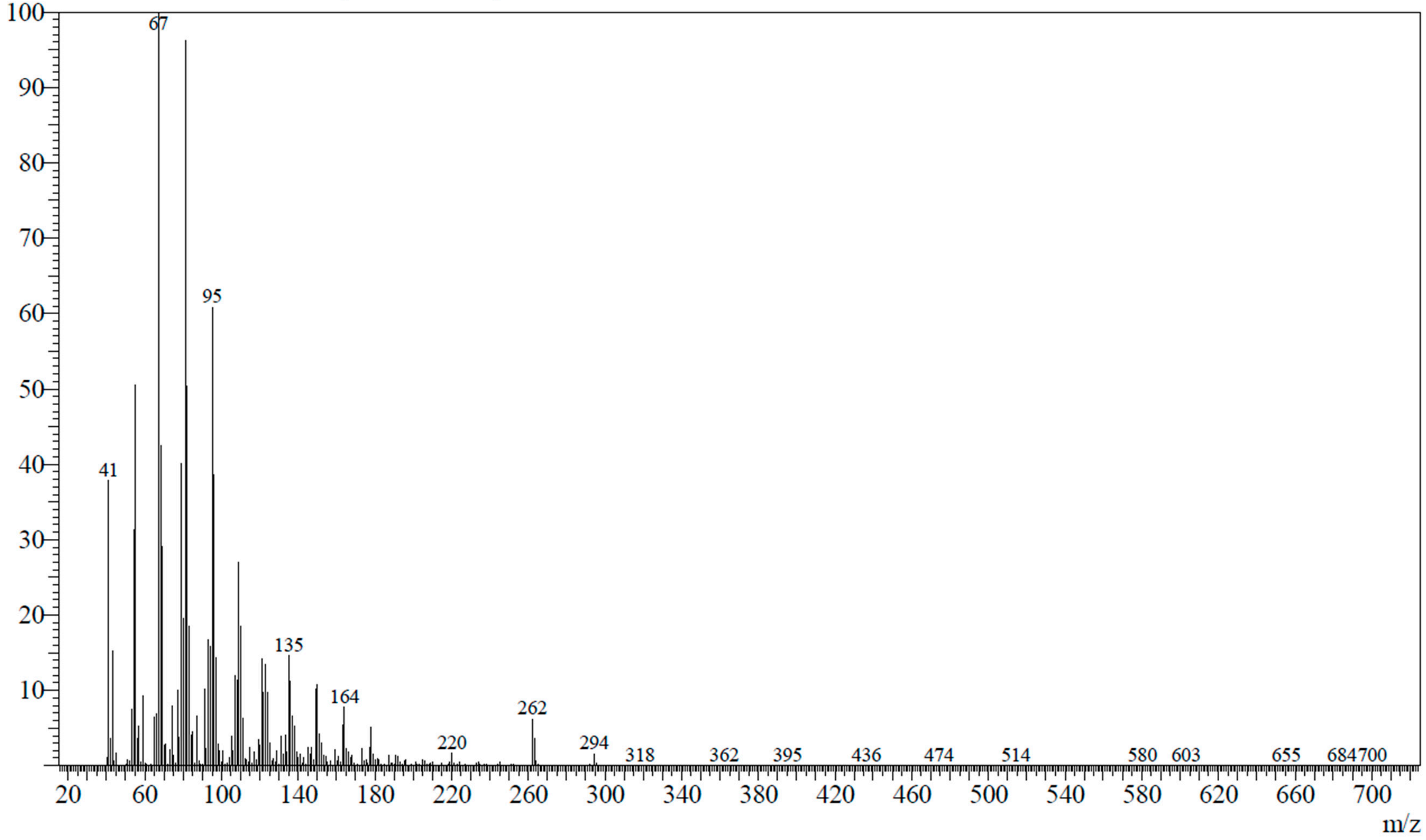

Oleic acid

Line#:48 R.Time:16.380(Scan#:2477)
MassPeaks:419
RawMode:Averaged 16.375-16.385(2476-2478) BasePeak:55(3566710)
BG Mode:Calc. from Peak Group 1 - Event 1 Q3 Scan

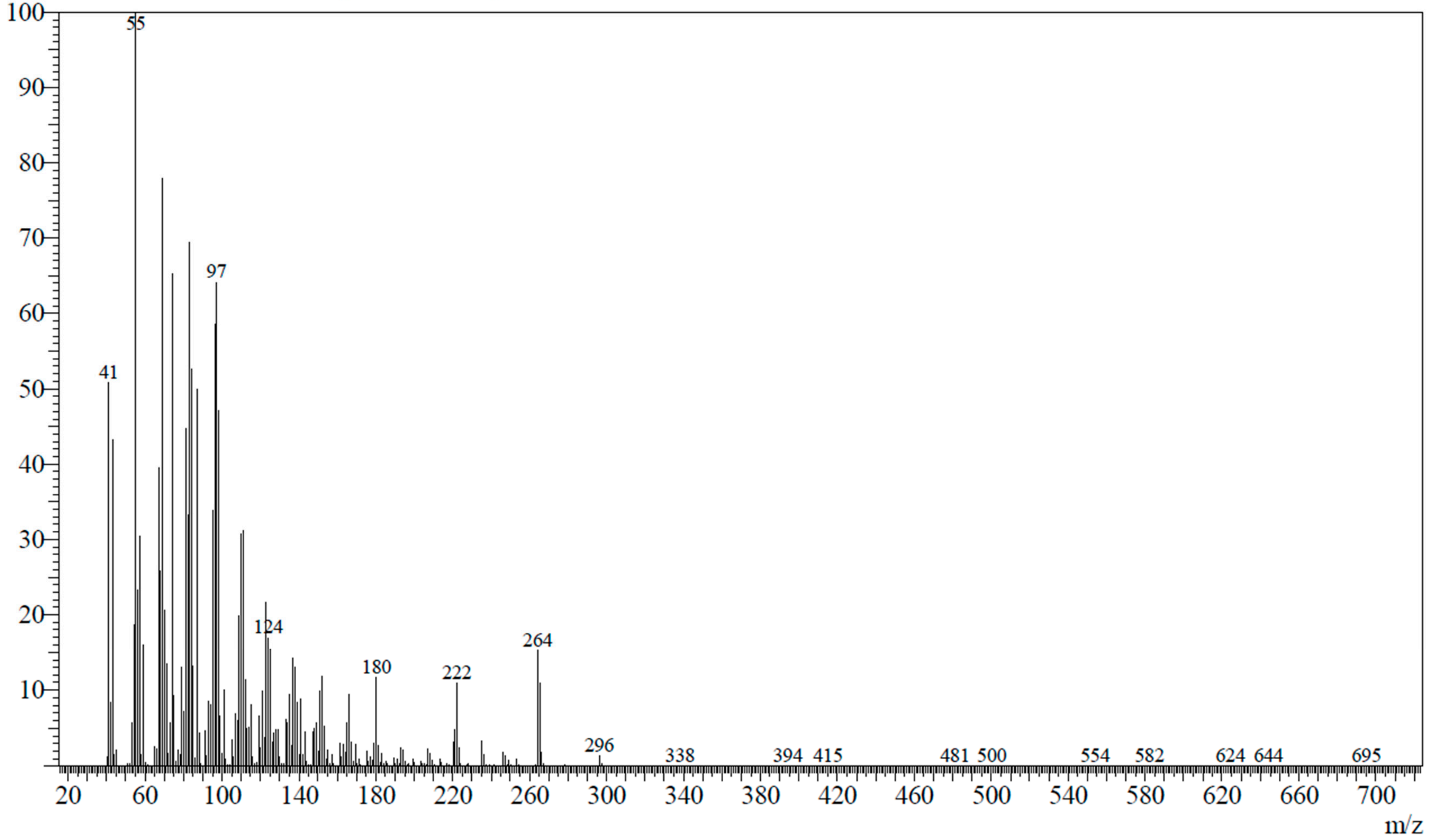

Elaidic acid

Line#:49 R.Time:16.465(Scan#:2494)
MassPeaks:423
RawMode:Averaged 16.460-16.470(2493-2495) BasePeak:55(225884)
BG Mode:Calc. from Peak Group 1 - Event 1 Q3 Scan

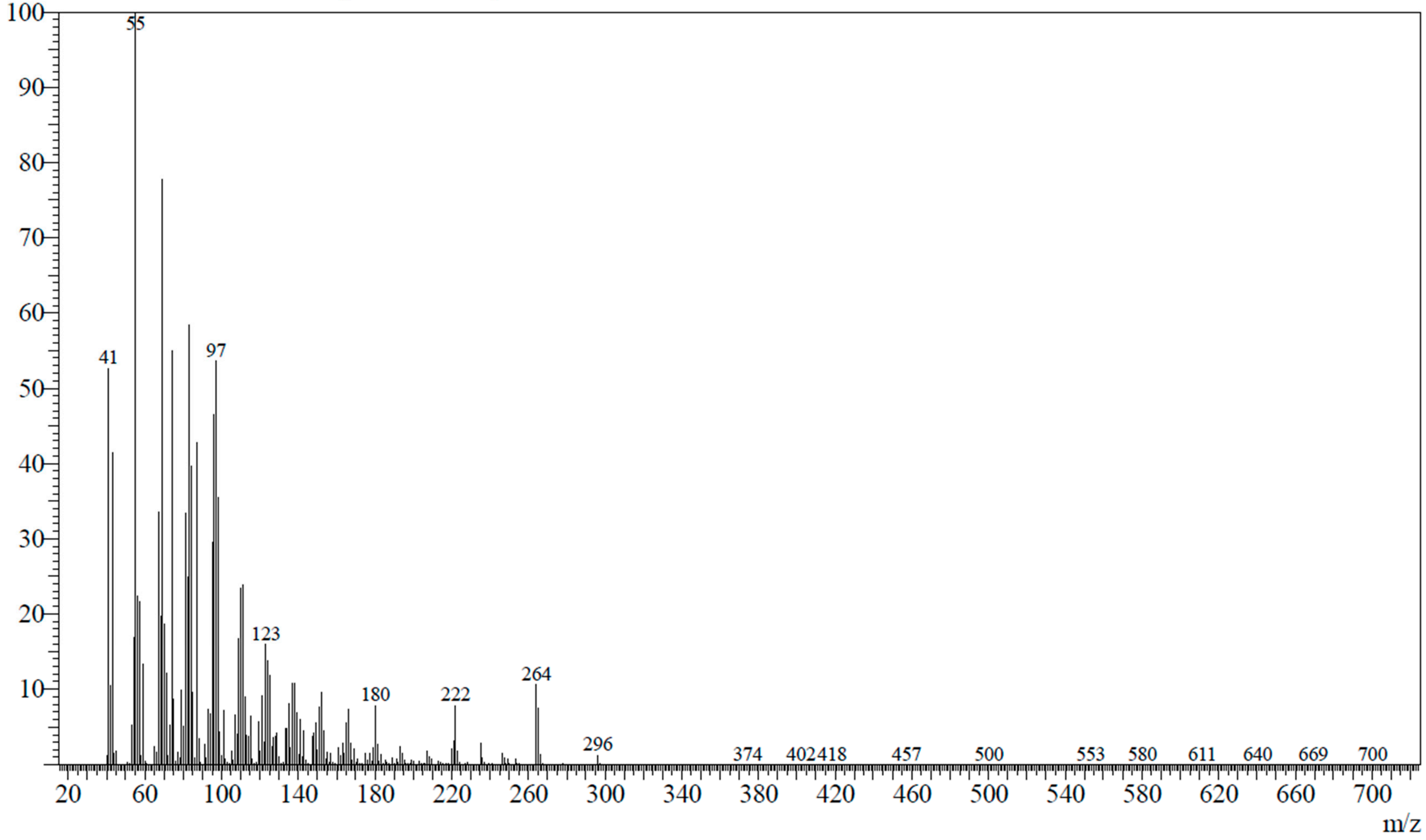

Stearic acid

Line#:50 R.Time:16.855(Scan#:2572)
MassPeaks:416
RawMode:Averaged 16.850-16.860(2571-2573) BasePeak:74(458457)
BG Mode:Calc. from Peak Group 1 - Event 1 Q3 Scan

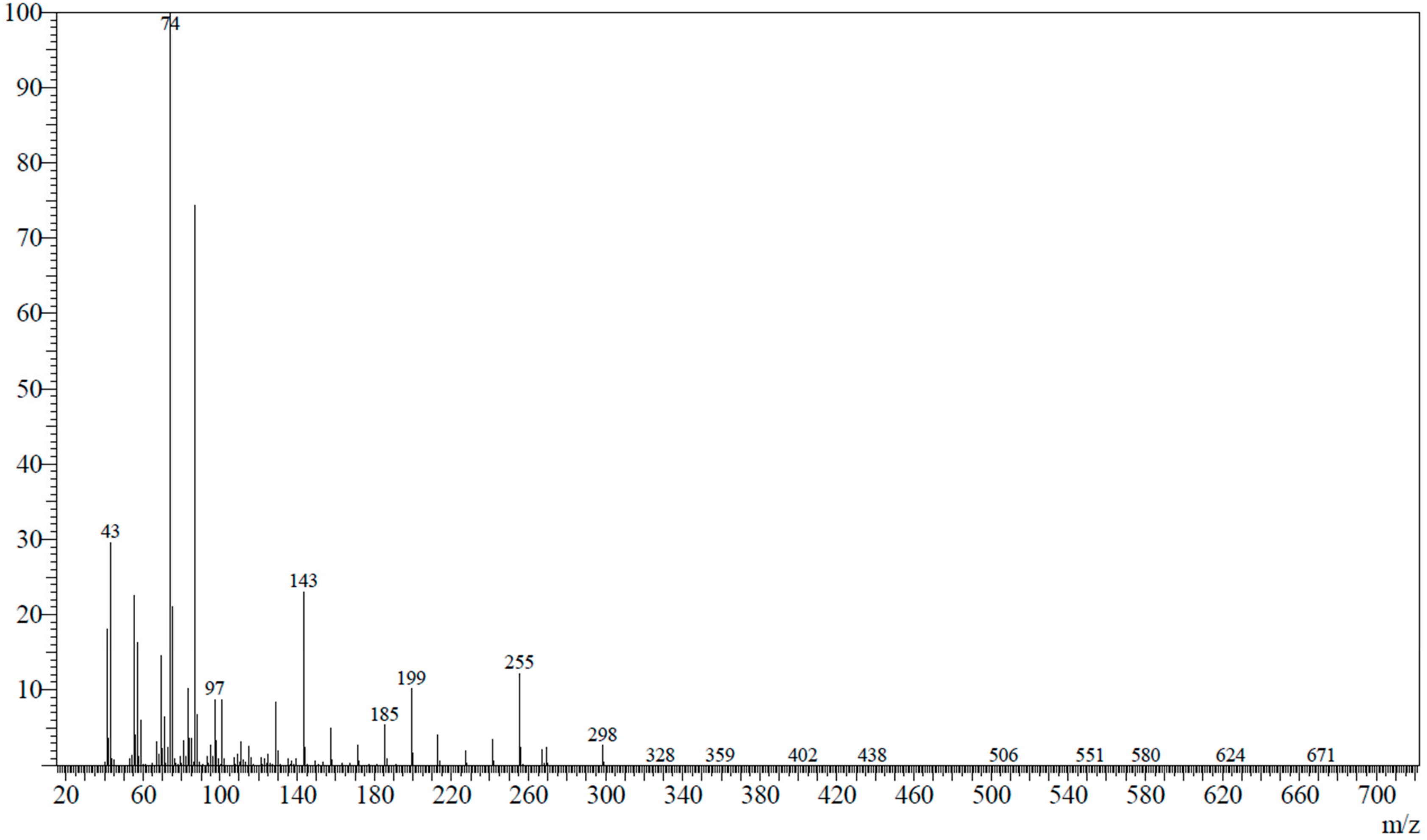

Figure S3. Total-ion-current (TIC) chromatograms of gabiroba waste oil obtained by Soxhlet extraction (SE).

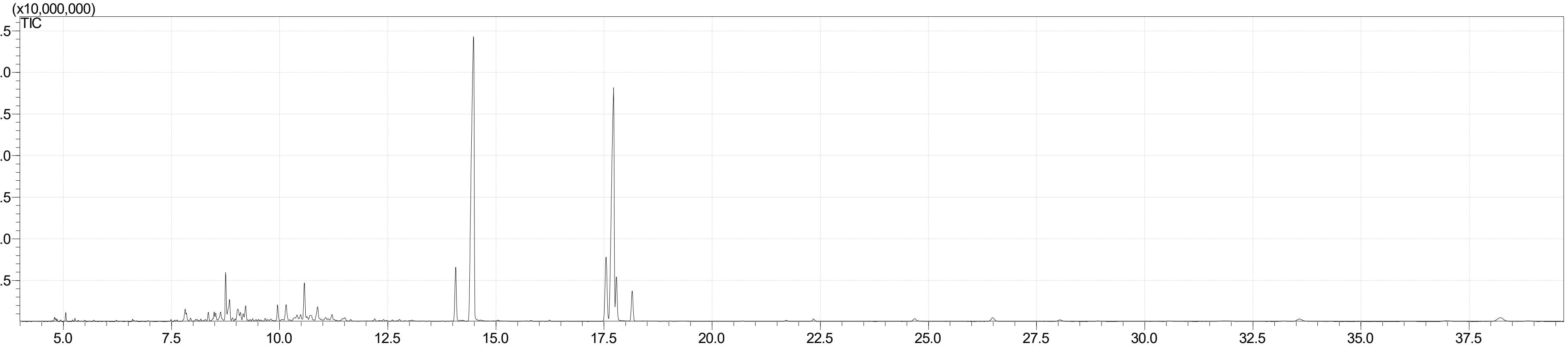

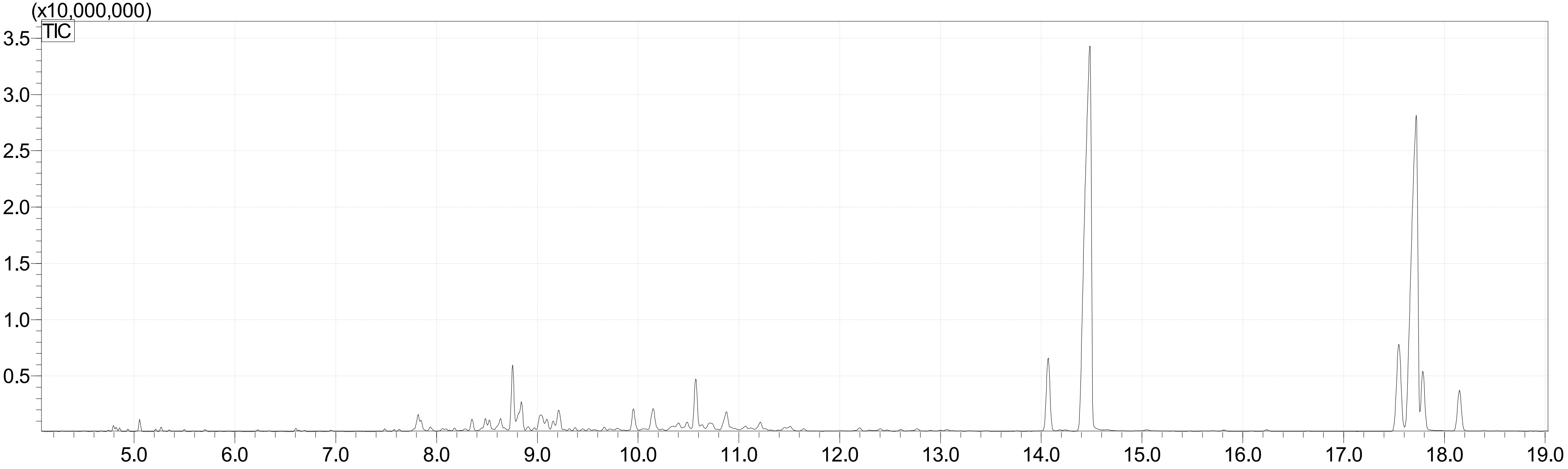

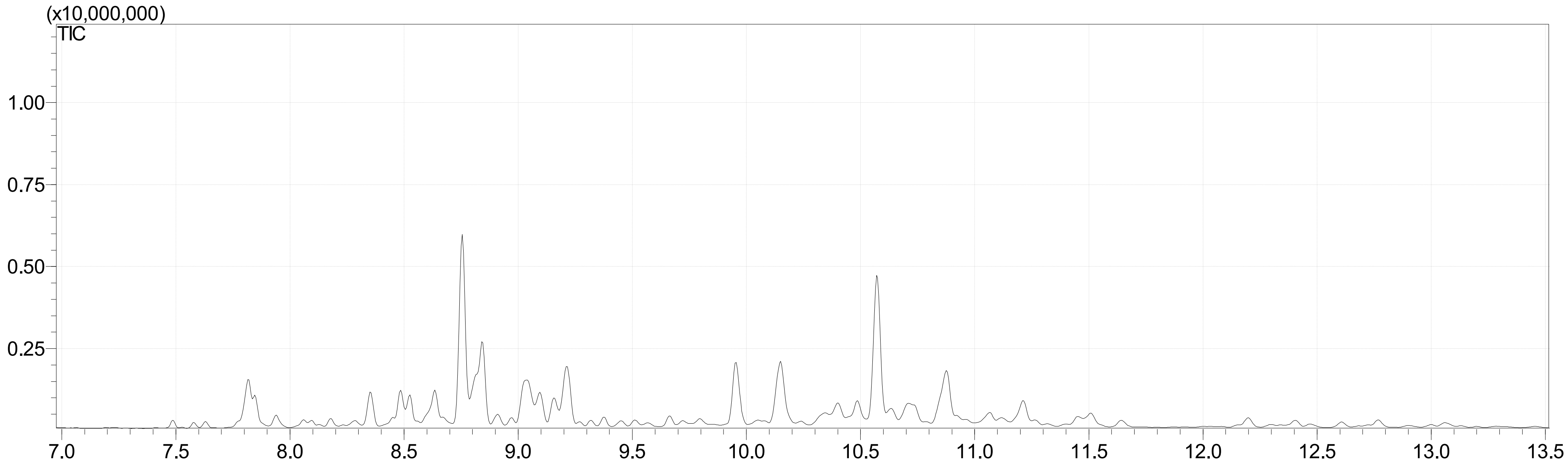
(x10,000,000)
TIC
1.00
0.75
0.50
0.25
7.0 7.5 8.0 8.5 9.0 9.5 10.0 10.5 11.0 11.5 12.0 12.5 13.0 13.5

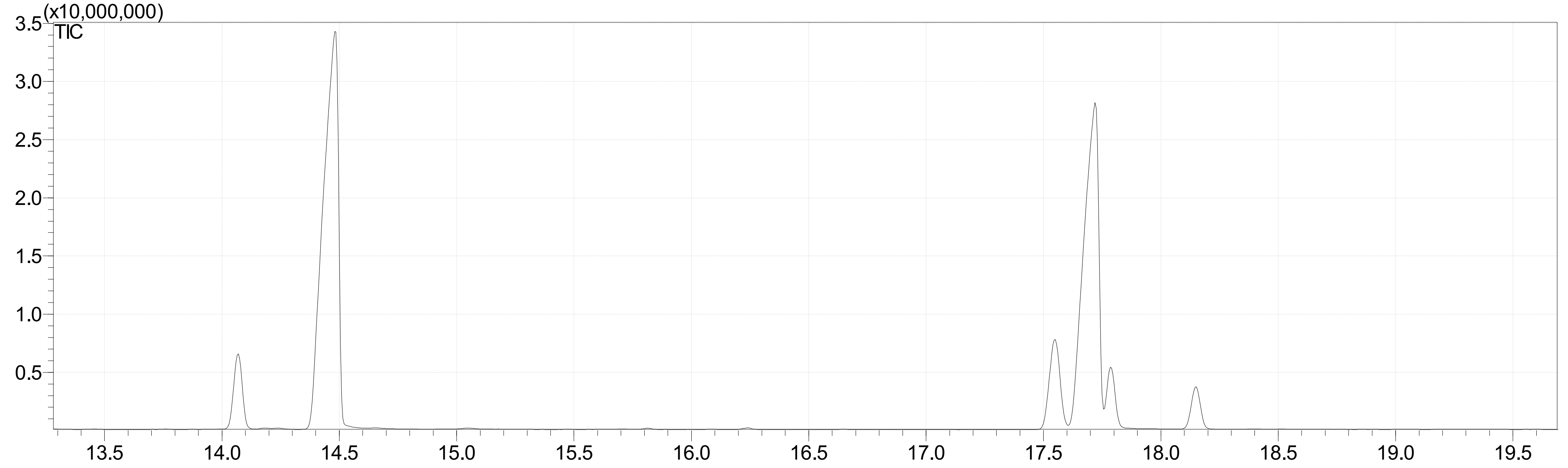
(x10,000,000)
TIC
3.5
3.0
2.5
2.0
1.5
1.0
0.5
13.5 14.0 14.5 15.0 15.5 16.0 16.5 17.0 17.5 18.0 18.5 19.0 19.5

Figure S4. Experimental mass spectra of the major compounds identified in gabiroba waste oil obtained by Soxhlet extraction (SE).

β-Patchoulene

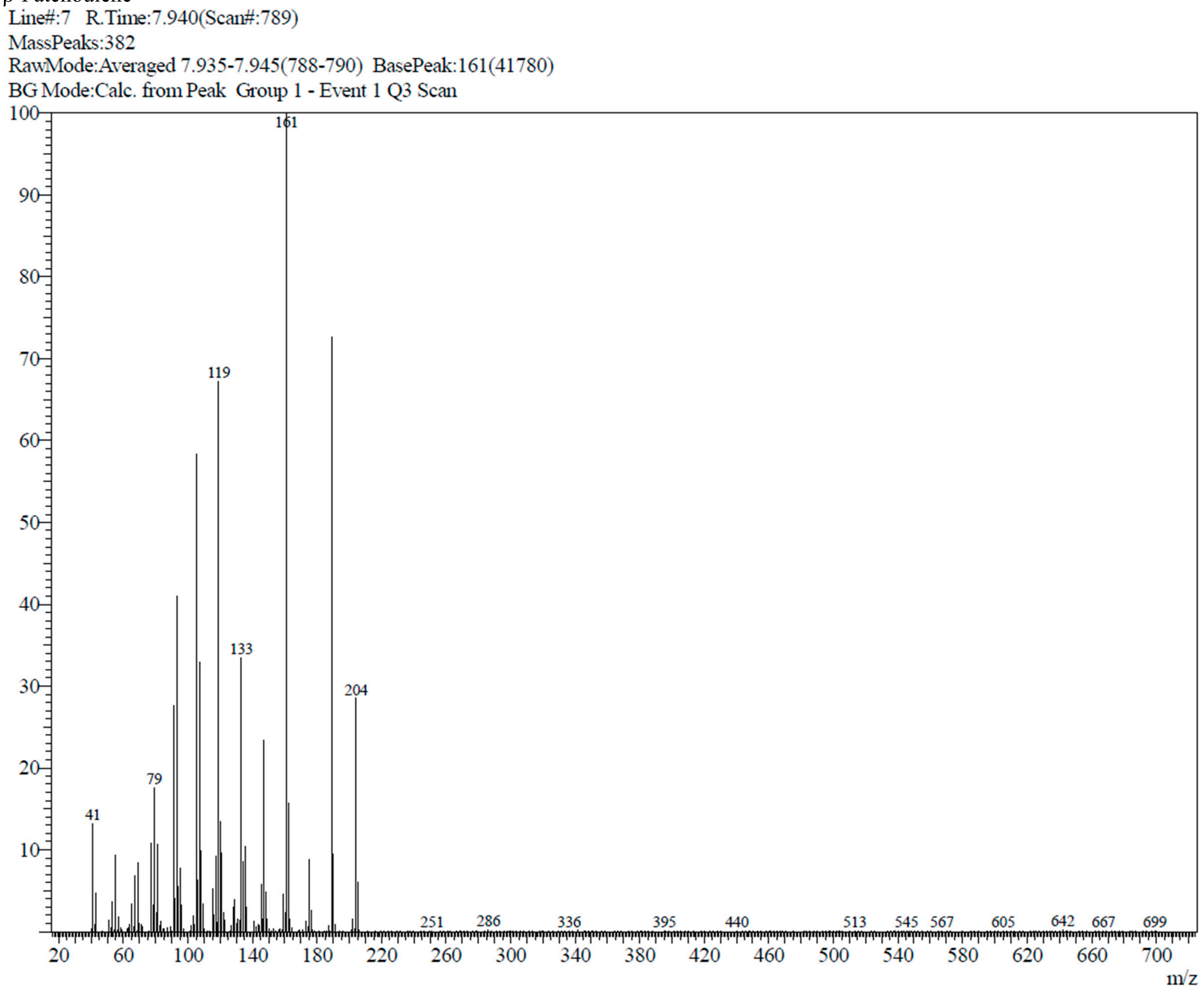

α-Copaene

Line#:11 R.Time:8.445(Scan#:890)
MassPeaks:359
RawMode:Averaged 8.440-8.450(889-891) BasePeak:119(5022)
BG Mode:Calc. from Peak Group 1 - Event 1 Q3 Scan

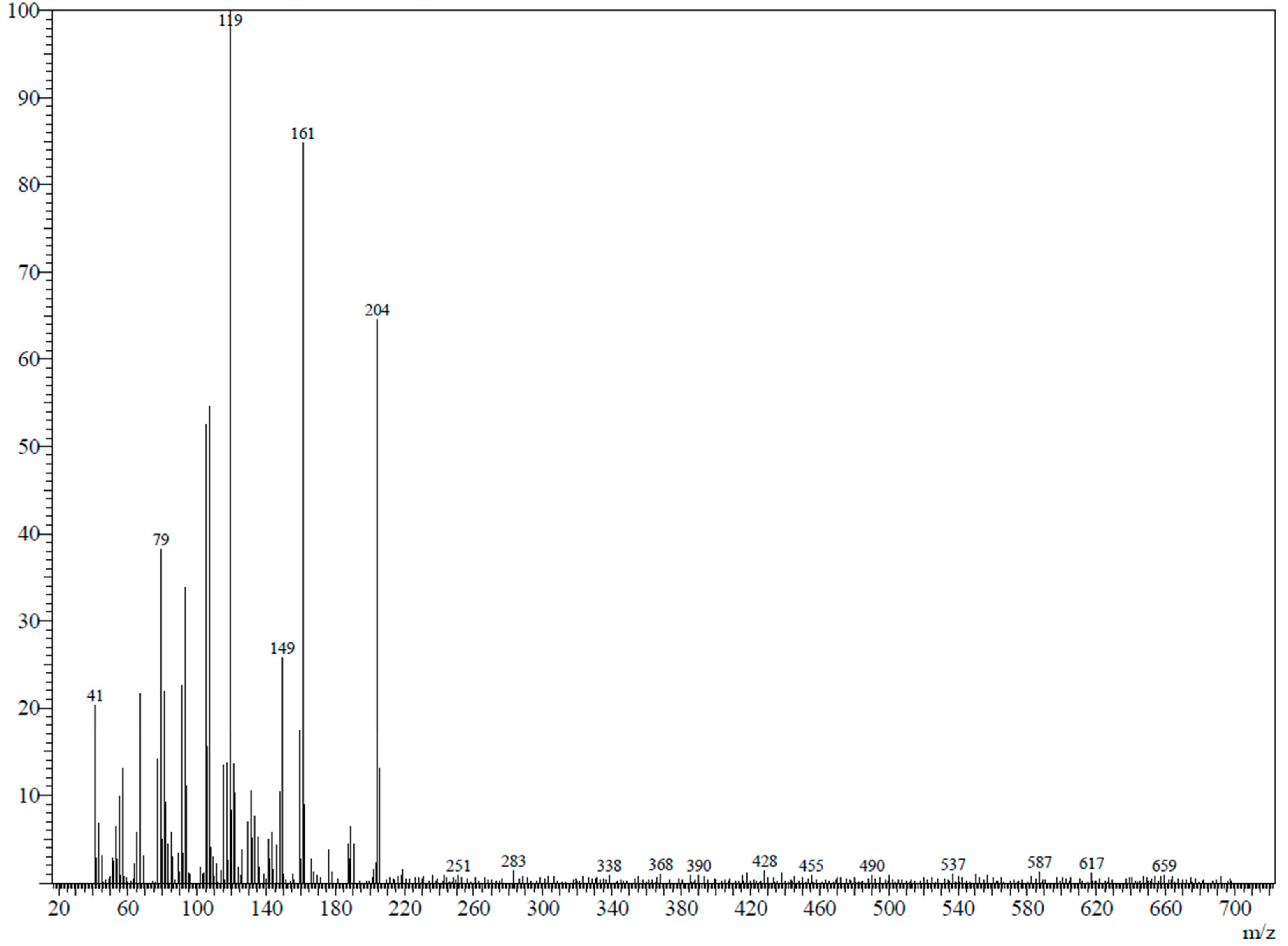

β-Maliene

Line#:12 R.Time:8.485(Scan#:898)
MassPeaks:407
RawMode:Averaged 8.480-8.490(897-899) BasePeak:105(60210)
BG Mode:Calc. from Peak Group 1 - Event 1 Q3 Scan

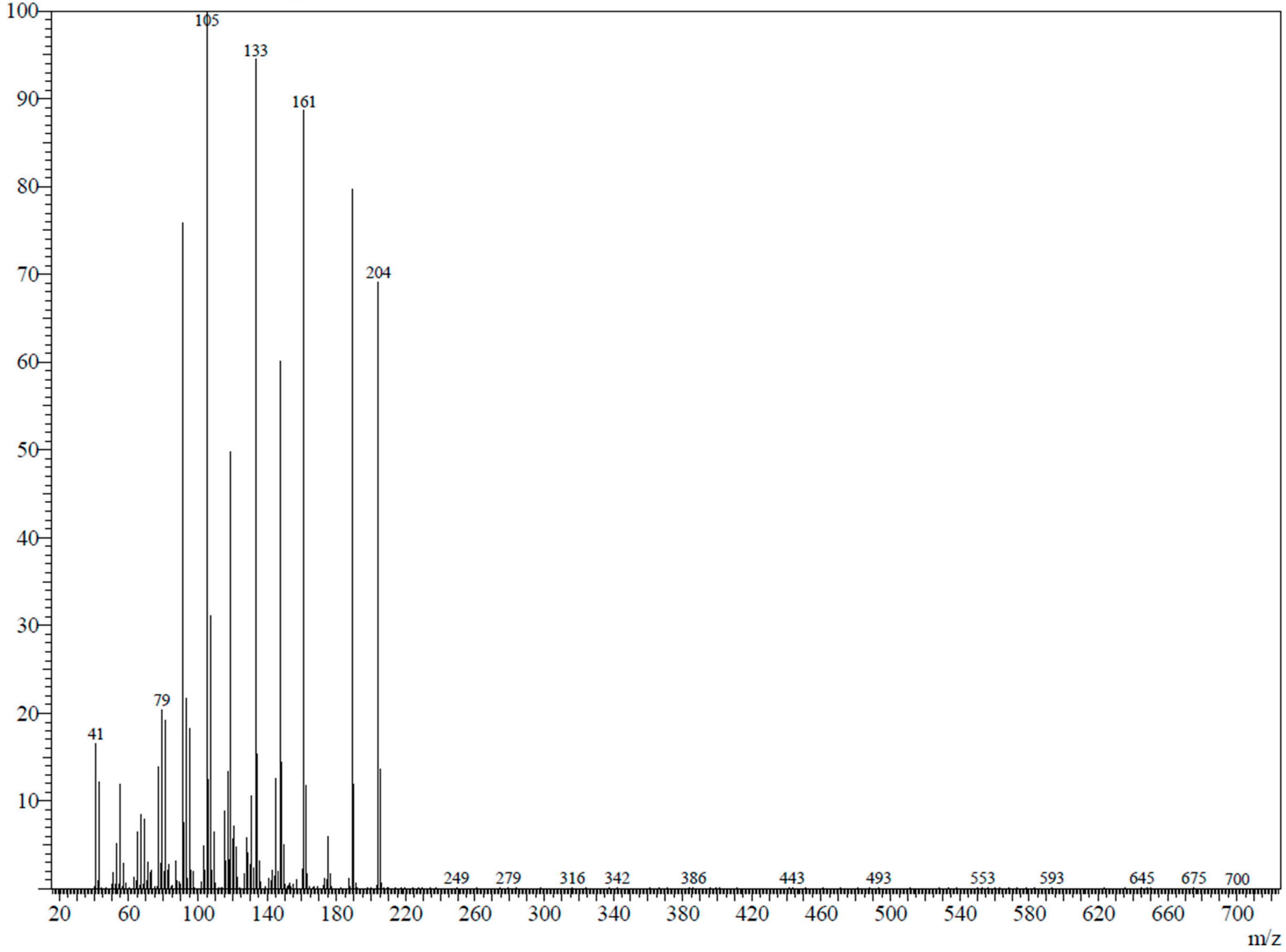

β-Cadinene

Line#:16 R.Time:8.755(Scan#:952)
MassPeaks:383
RawMode:Averaged 8.750-8.760(951-953) BasePeak:161(667396)
BG Mode:Calc. from Peak Group 1 - Event 1 Q3 Scan

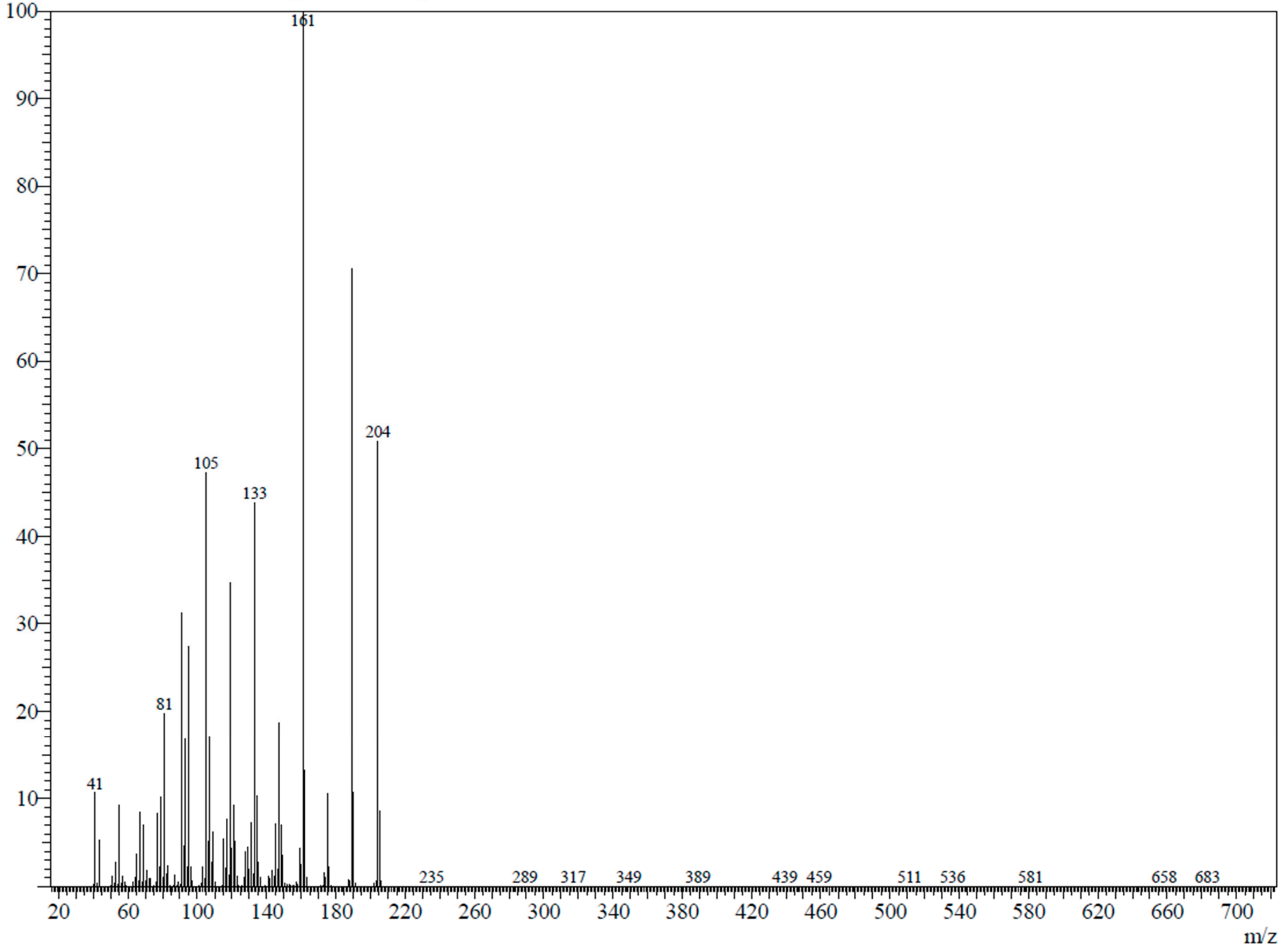

Valencene
Line#:13 R.Time:8.525(Scan#:906)
MassPeaks:375
RawMode:Averaged 8.520-8.530(905-907) BasePeak:161(55896)
BG Mode:Calc. from Peak Group 1 - Event 1 Q3 Scan

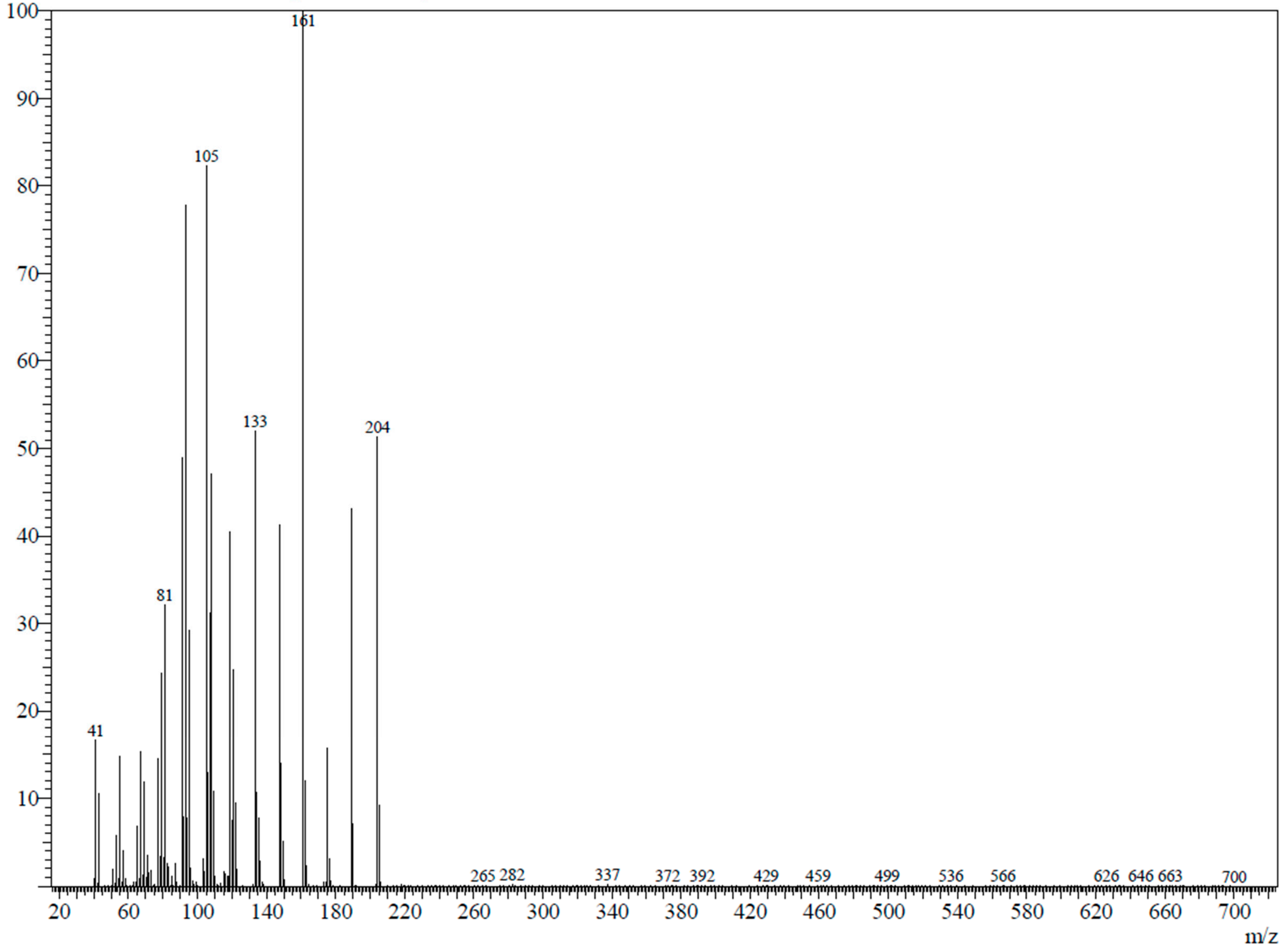

α-Murolene

Line#:17 R.Time:8.840(Scan#:969)
MassPeaks:403
RawMode:Averaged 8.835-8.845(968-970) BasePeak:105(259601)
BG Mode:Calc. from Peak Group 1 - Event 1 Q3 Scan

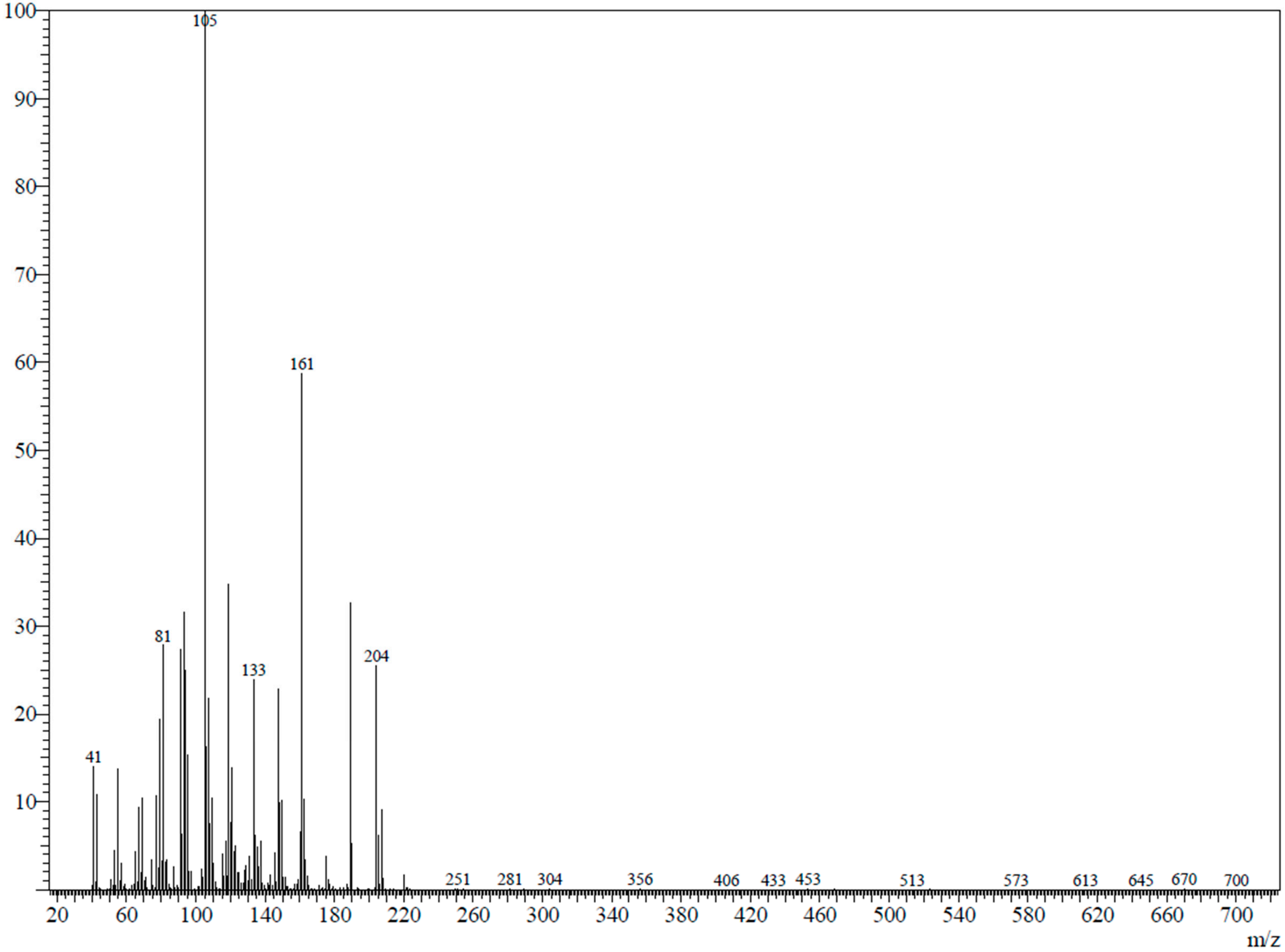

Cis-Calamenene

Line#:21 R.Time:9.095(Scan#:1020)
MassPeaks:304
RawMode:Averaged 9.090-9.100(1019-1021) BasePeak:159(324791)
BG Mode:Calc. from Peak Group 1 - Event 1 Q3 Scan

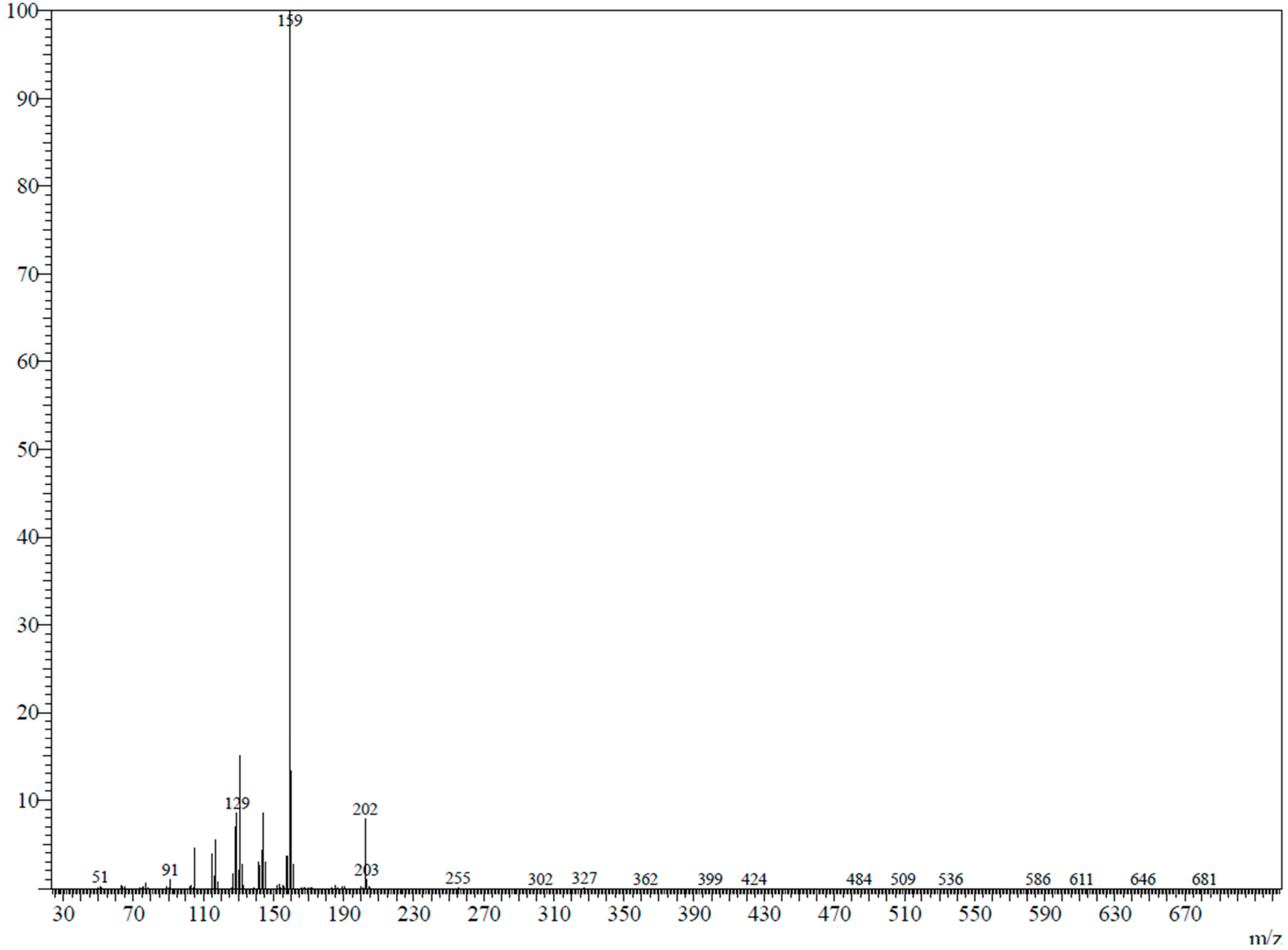

γ-Murolene
Line#:22   R.Time:9.155(Scan#:1032)
MassPeaks:337
RawMode:Averaged 9.150-9.160(1031-1033) BasePeak:161(72398)
BG Mode:Calc. from Peak Group 1 - Event 1 Q3 Scan

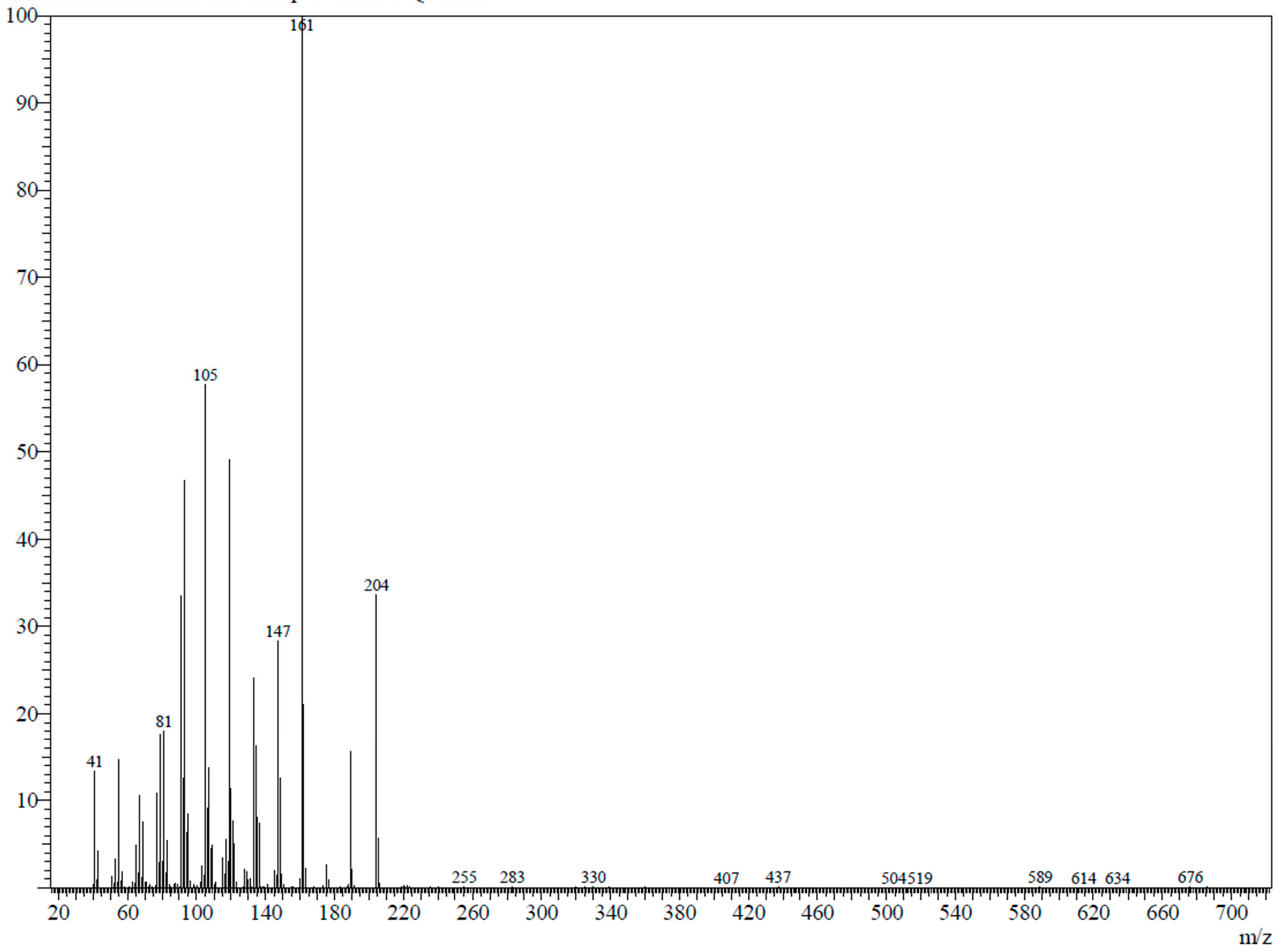

Isoledene

Line#:23 R.Time:9.210(Scan#:1043)
MassPeaks:396
RawMode:Averaged 9.205-9.215(1042-1044) BasePeak:119(169213)
BG Mode:Calc. from Peak Group 1 - Event 1 Q3 Scan

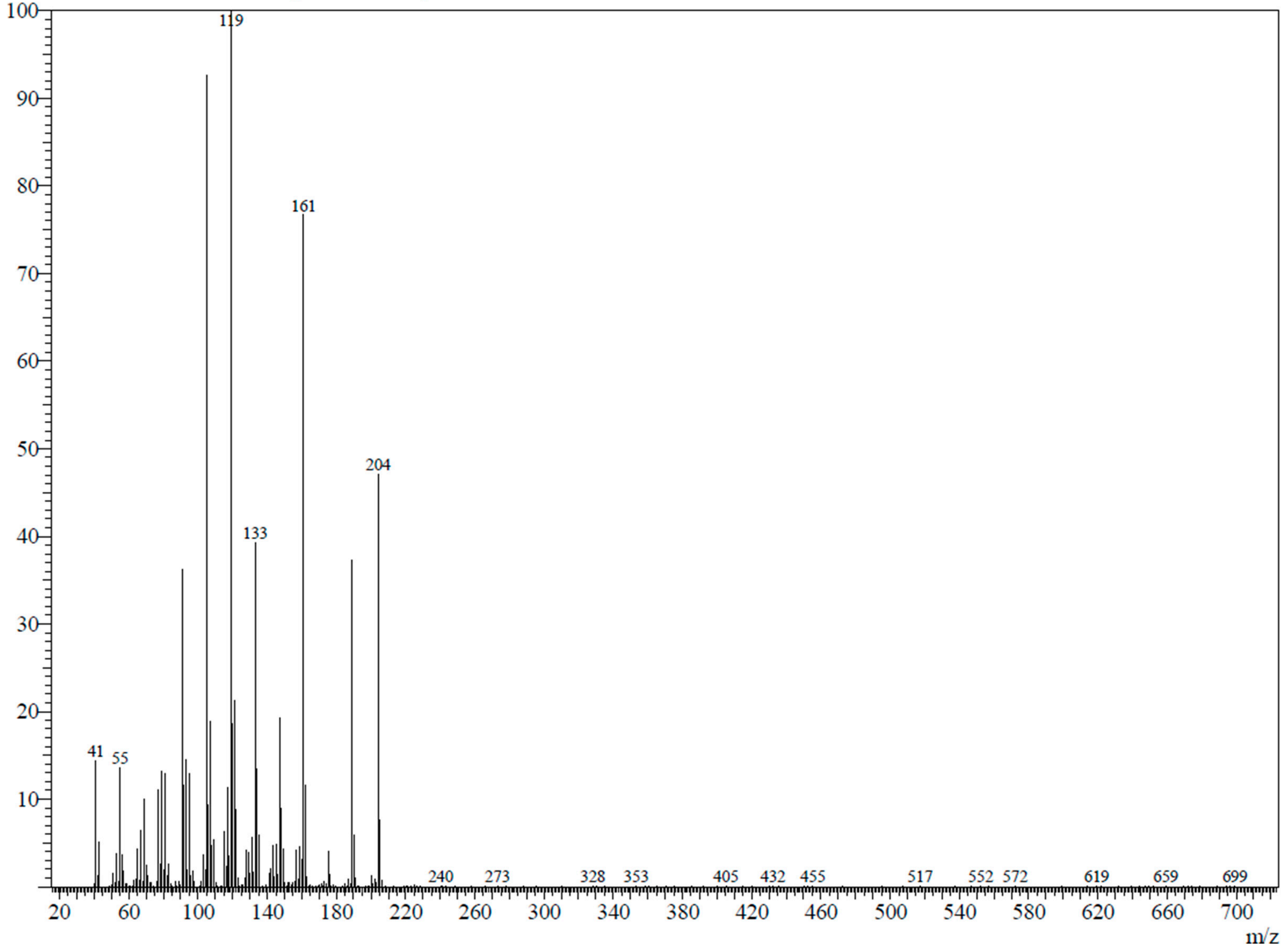

Selina-3,7(11)-diene

Line#:24 R.Time:9.375(Scan#:1076)
MassPeaks:388
RawMode:Averaged 9.370-9.380(1075-1077) BasePeak:161(31146)
BG Mode:Calc. from Peak Group 1 - Event 1 Q3 Scan

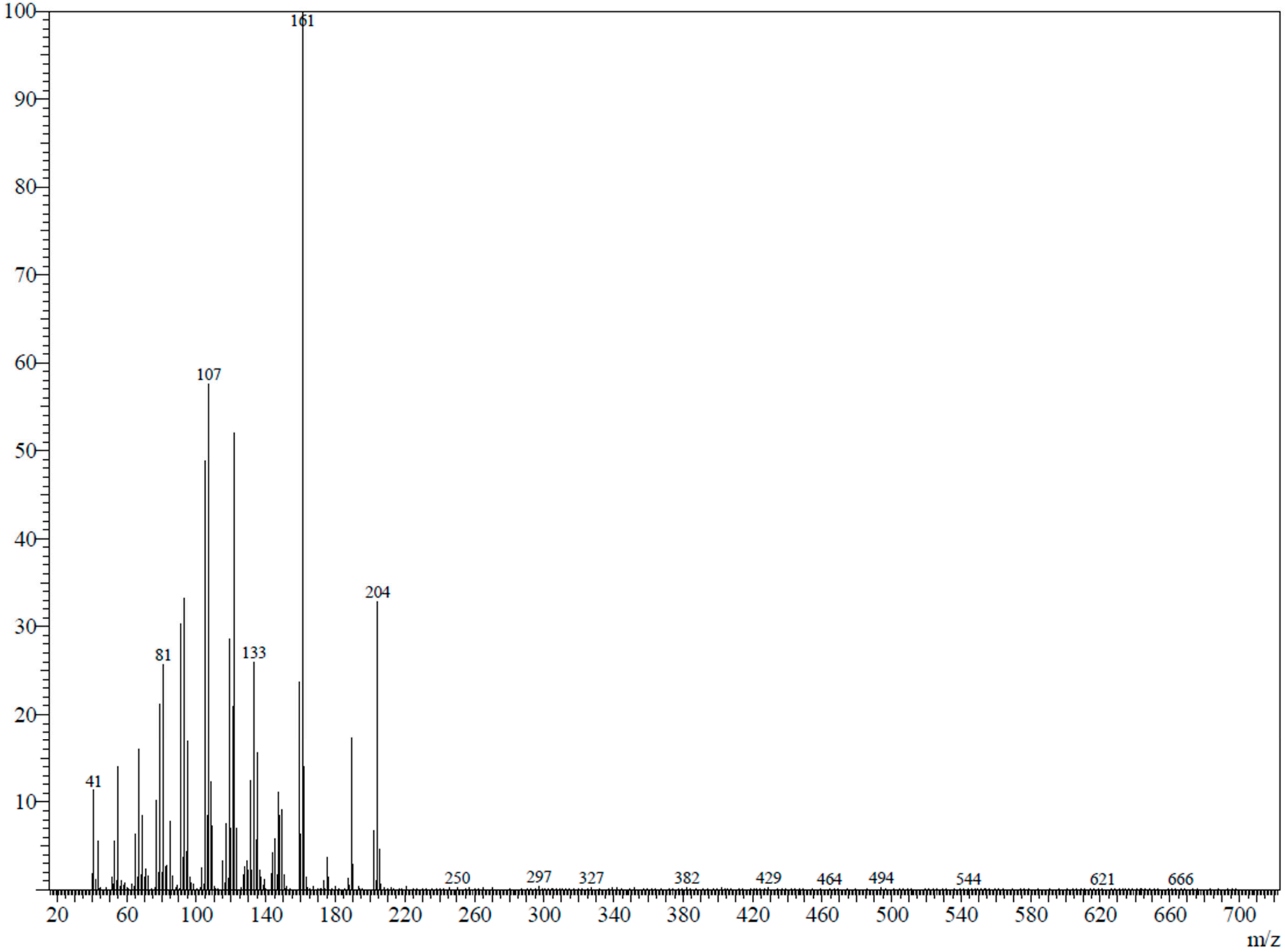

δ-Cadinene

Line#:27 R.Time:10.150(Scan#:1231)
MassPeaks:414
RawMode:Averaged 10.145-10.155(1230-1232) BasePeak:161(247543)
BG Mode:Calc. from Peak Group 1 - Event 1 Q3 Scan

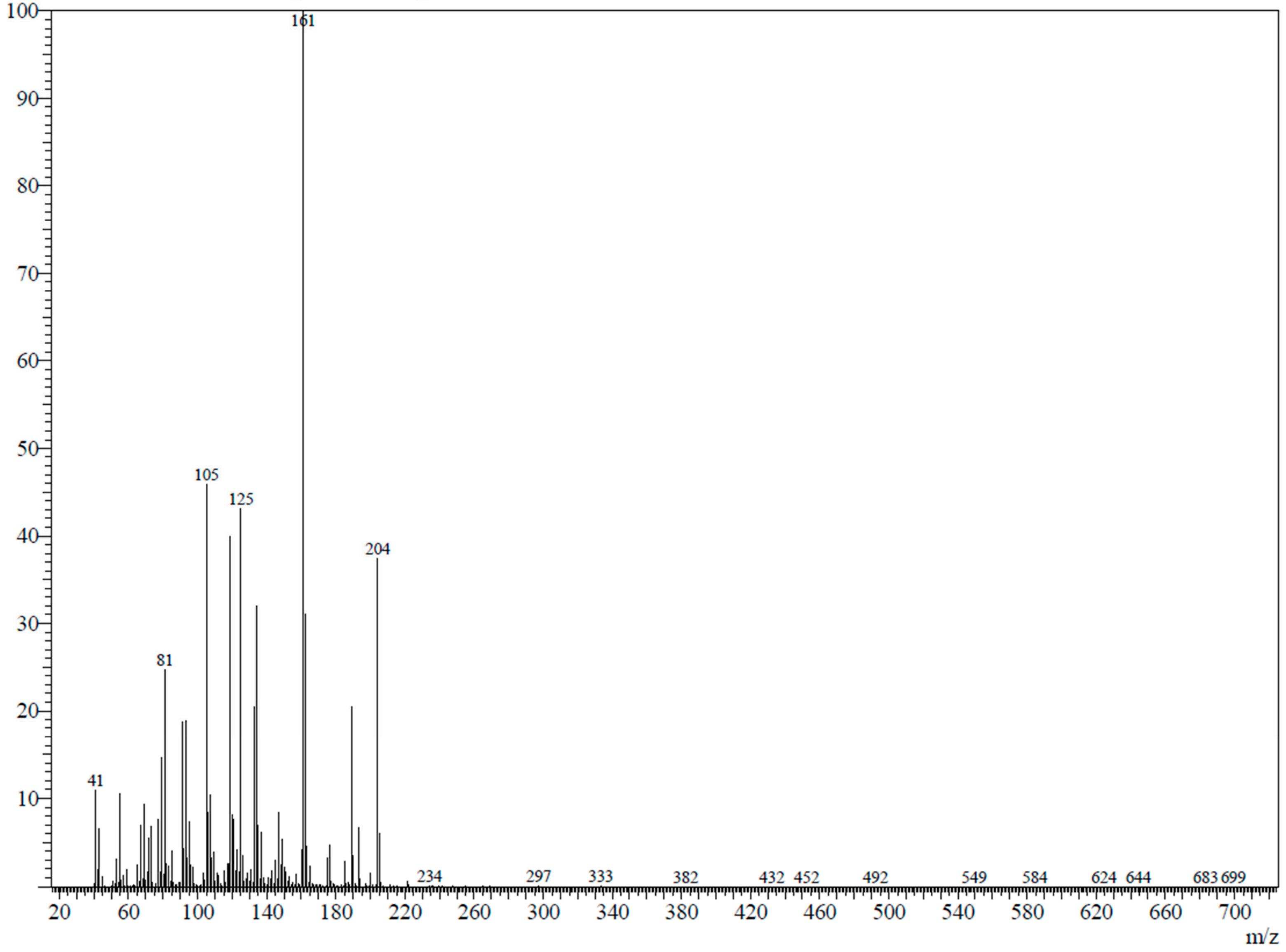

γ-Gurjunene

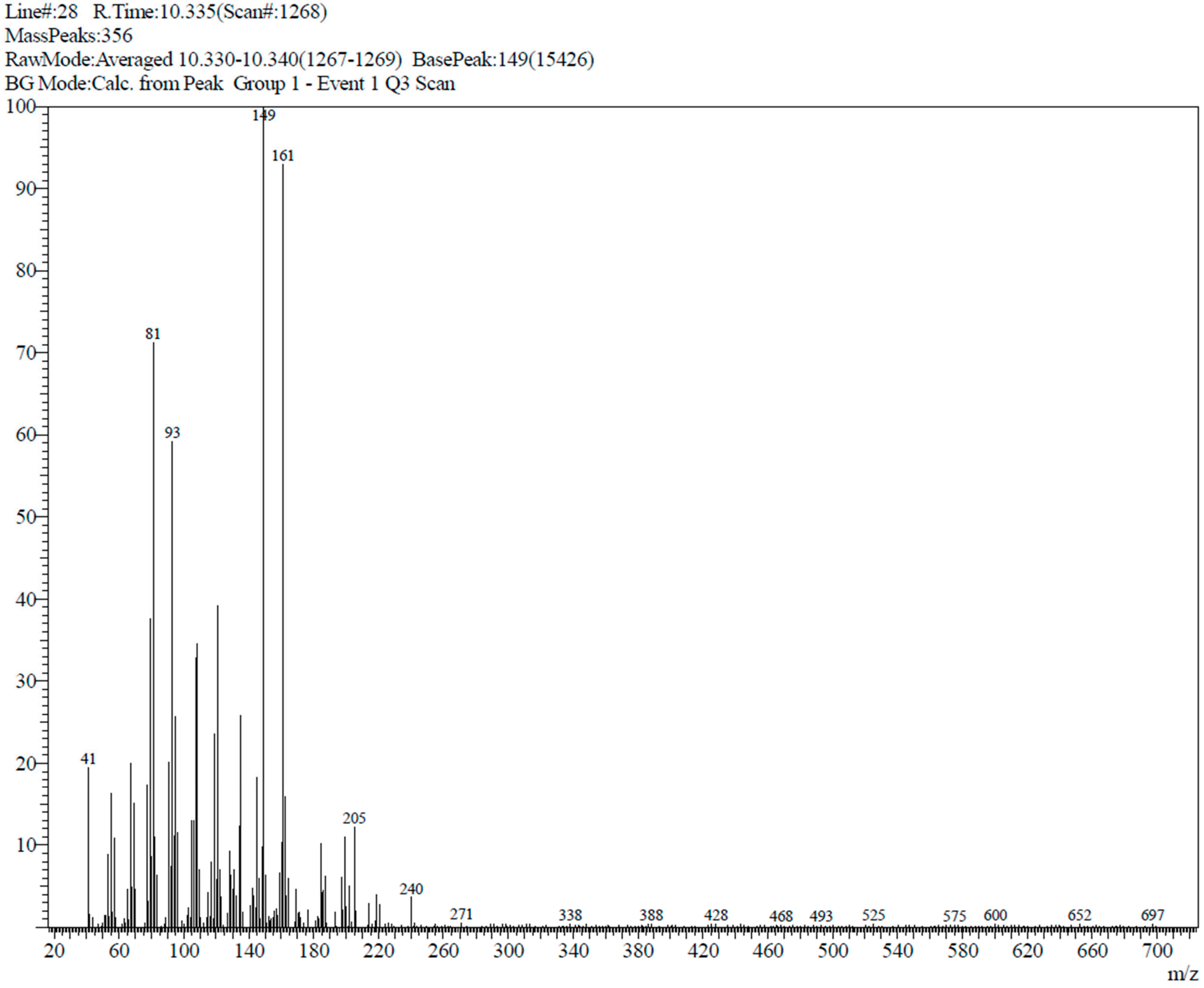

Line#:28 R.Time:10.335(Scan#:1268)
MassPeaks:356
RawMode:Averaged 10.330-10.340(1267-1269) BasePeak:149(15426)
BG Mode:Calc. from Peak Group 1 - Event 1 Q3 Scan
149
161
81
93
41
205
240
271
338
388
428
468
493
525
575
600
652
697
m/z

Ambrox

Line#:30 R.Time:10.485(Scan#:1298)
MassPeaks:377
RawMode:Averaged 10.480-10.490(1297-1299) BasePeak:221(181554)
BG Mode:Calc. from Peak Group 1 - Event 1 Q3 Scan

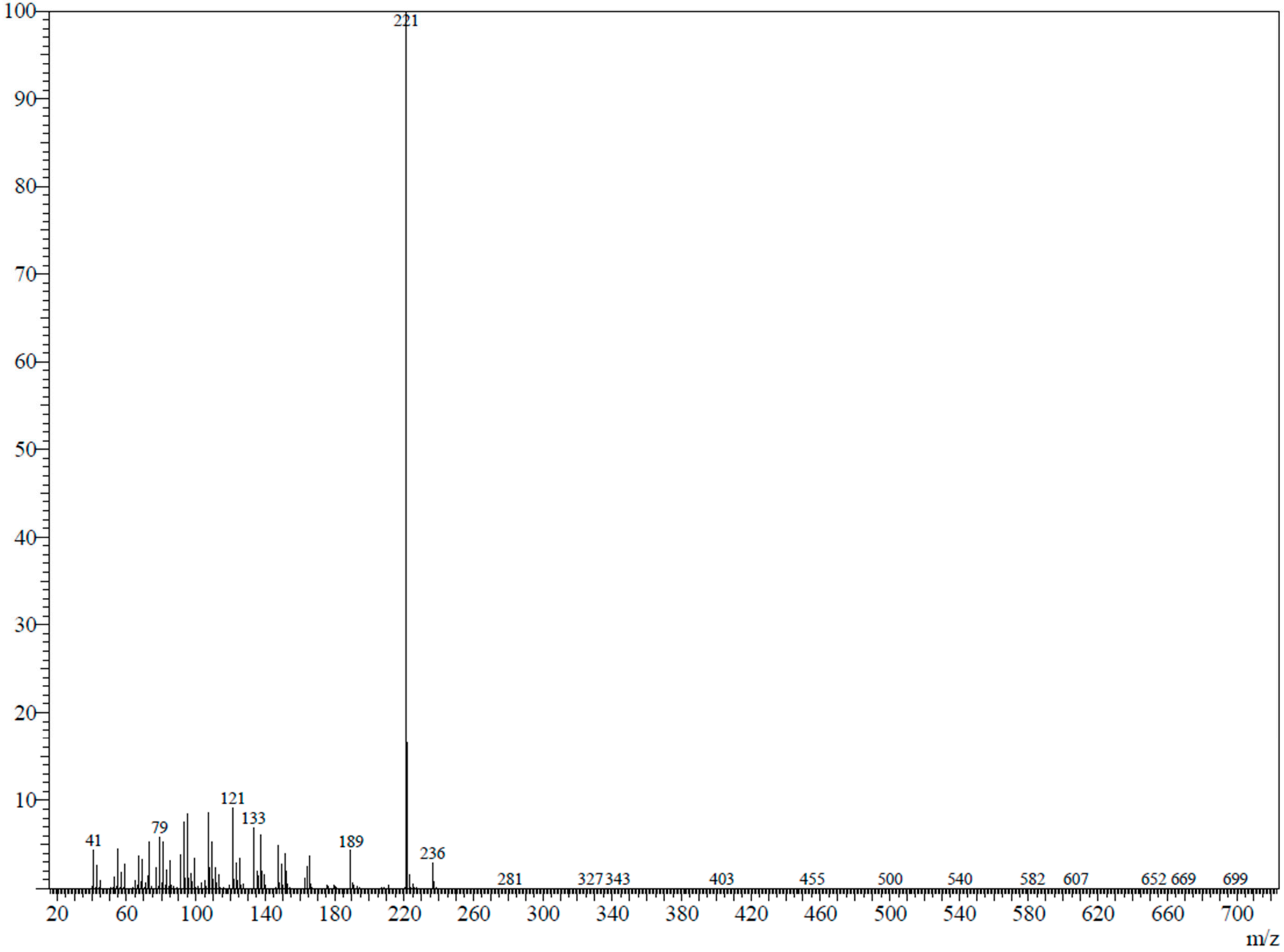

Clovene

Line#:31 R.Time:10.570(Scan#:1315)
MassPeaks:367
RawMode:Averaged 10.565-10.575(1314-1316) BasePeak:73(947683)
BG Mode:Calc. from Peak Group 1 - Event 1 Q3 Scan

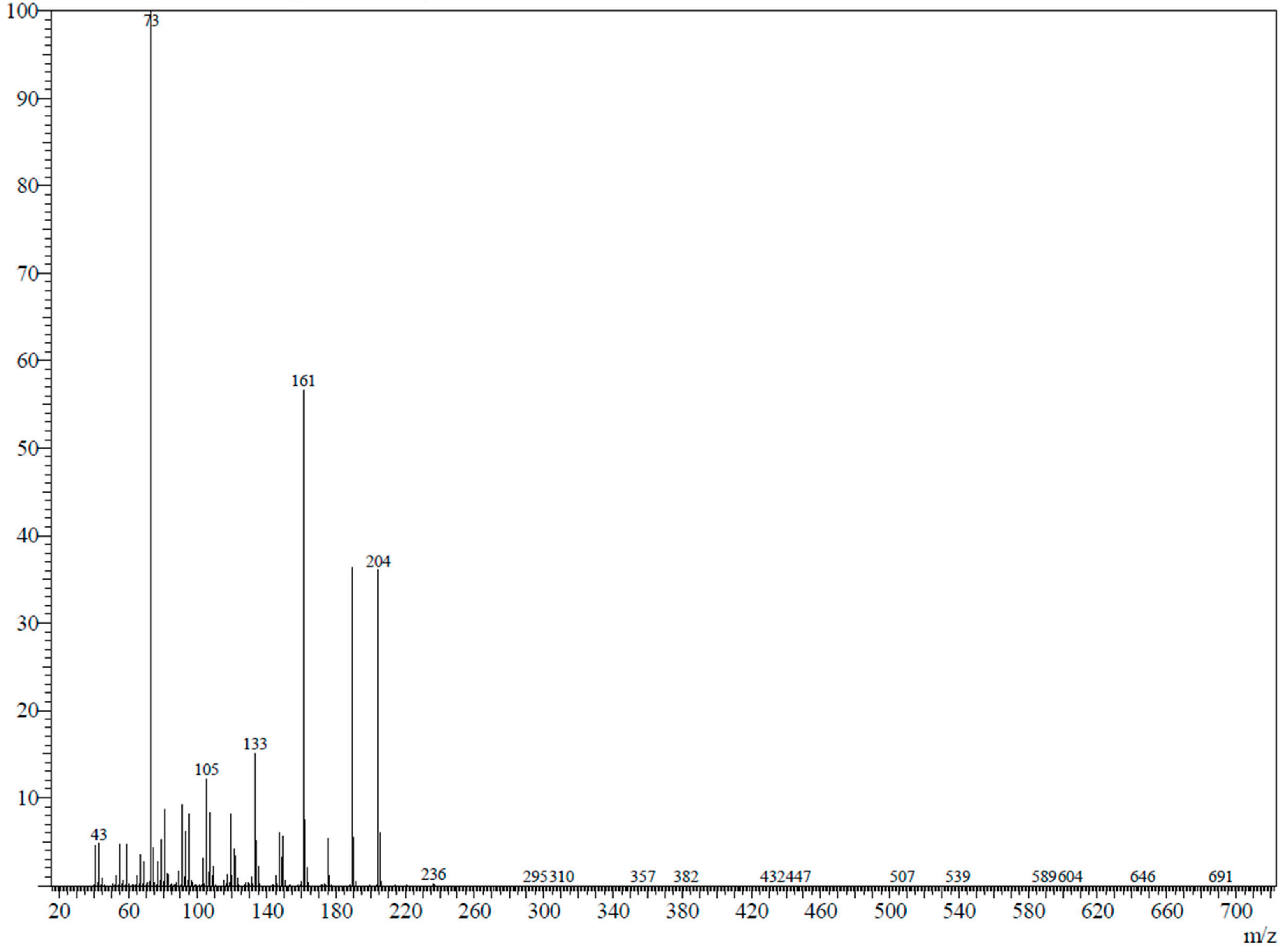

Aristolene

Line#:32 R.Time:10.635(Scan#:1328)
MassPeaks:343
RawMode:Averaged 10.630-10.640(1327-1329) BasePeak:105(30208)
BG Mode:Calc. from Peak Group 1 - Event 1 Q3 Scan

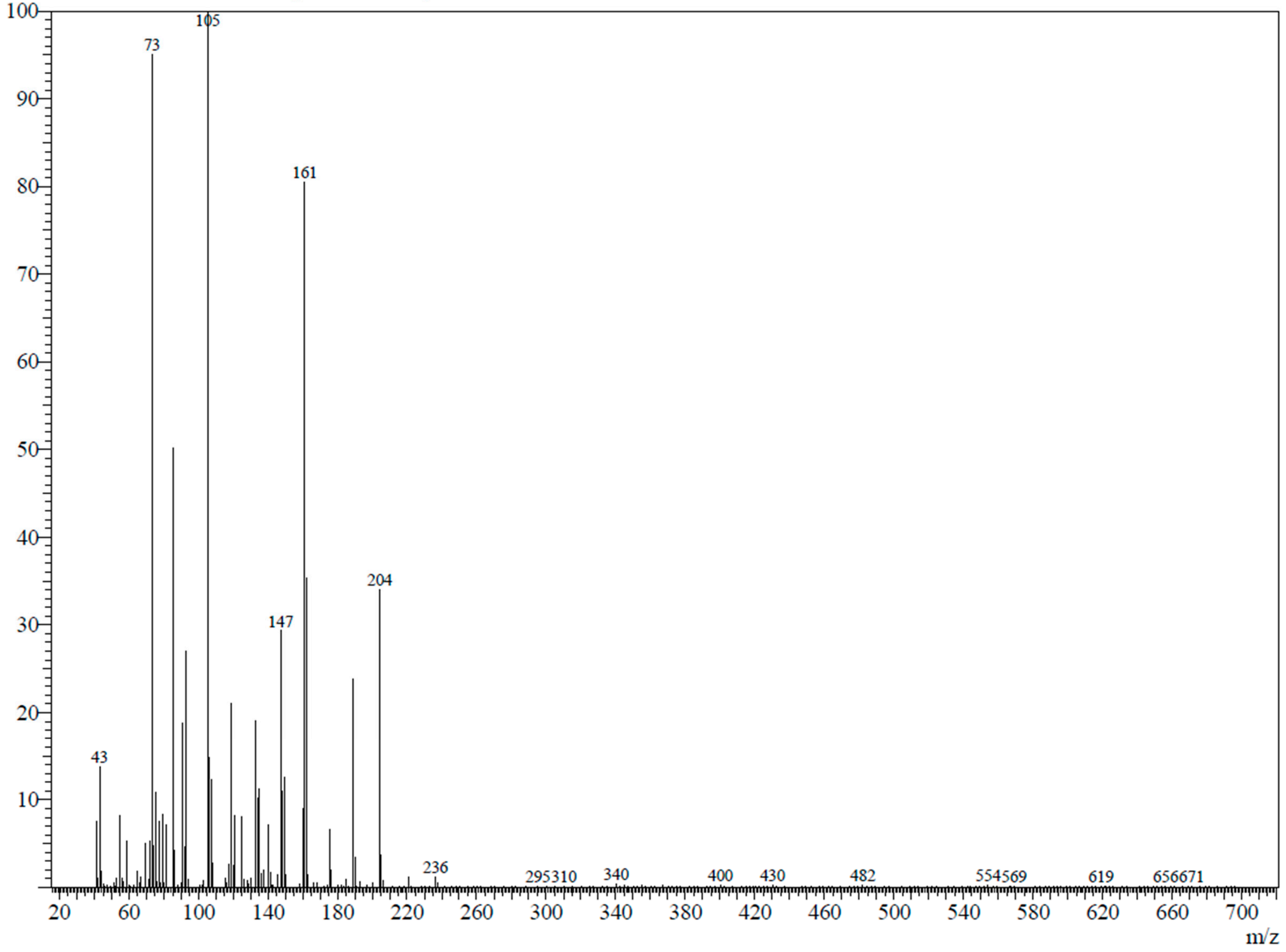

α-Cadinene

Line#:33 R.Time:10.710(Scan#:1343)
MassPeaks:379
RawMode:Averaged 10.705-10.715(1342-1344) BasePeak:73(135394)
BG Mode:Calc. from Peak Group 1 - Event 1 Q3 Scan

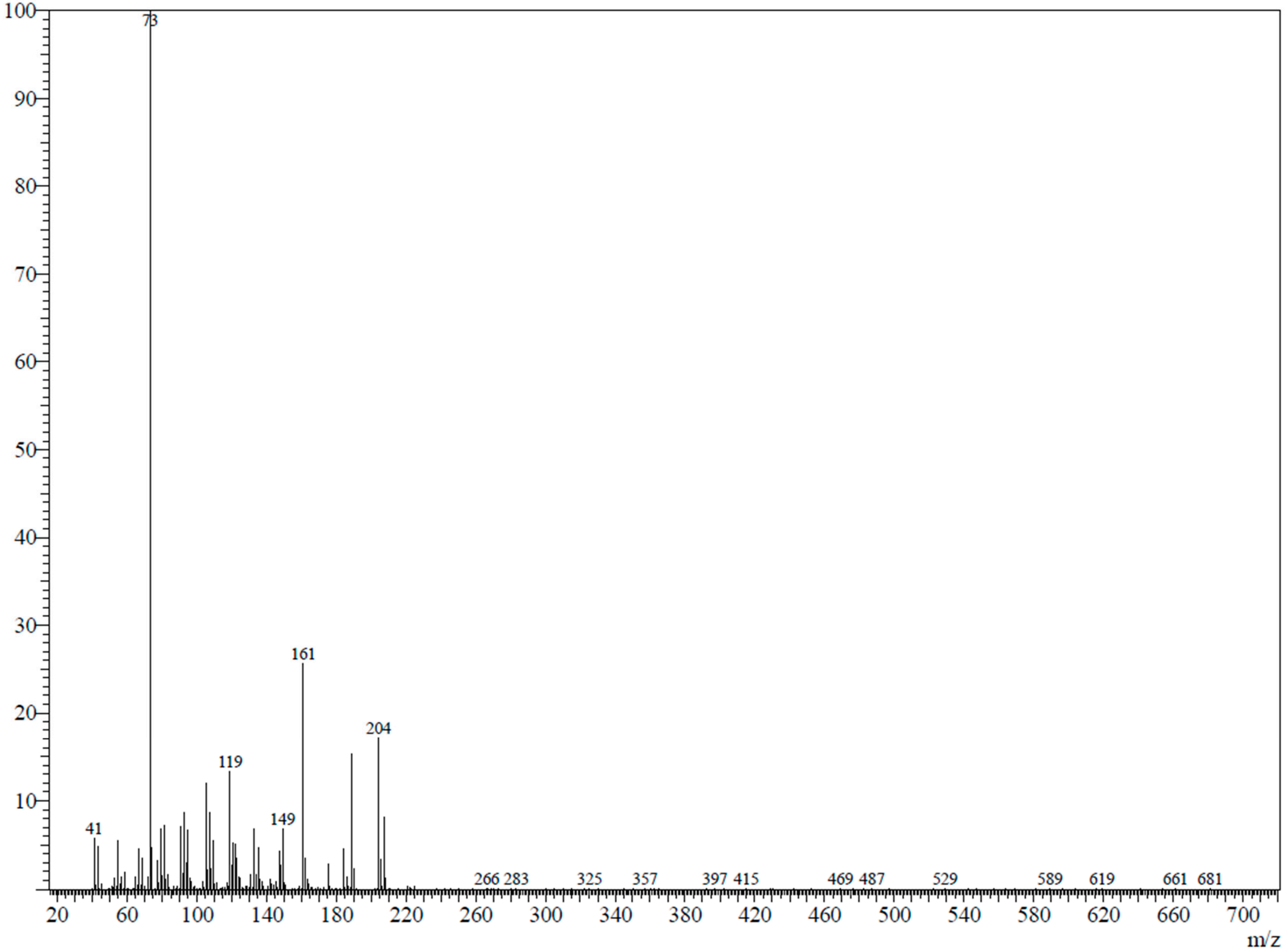

γ-Selinene
Line#:34   R.Time:10.875(Scan#:1376)
MassPeaks:397
RawMode:Averaged 10.870-10.880(1375-1377) BasePeak:189(213488)
BG Mode:Calc. from Peak Group 1 - Event 1 Q3 Scan

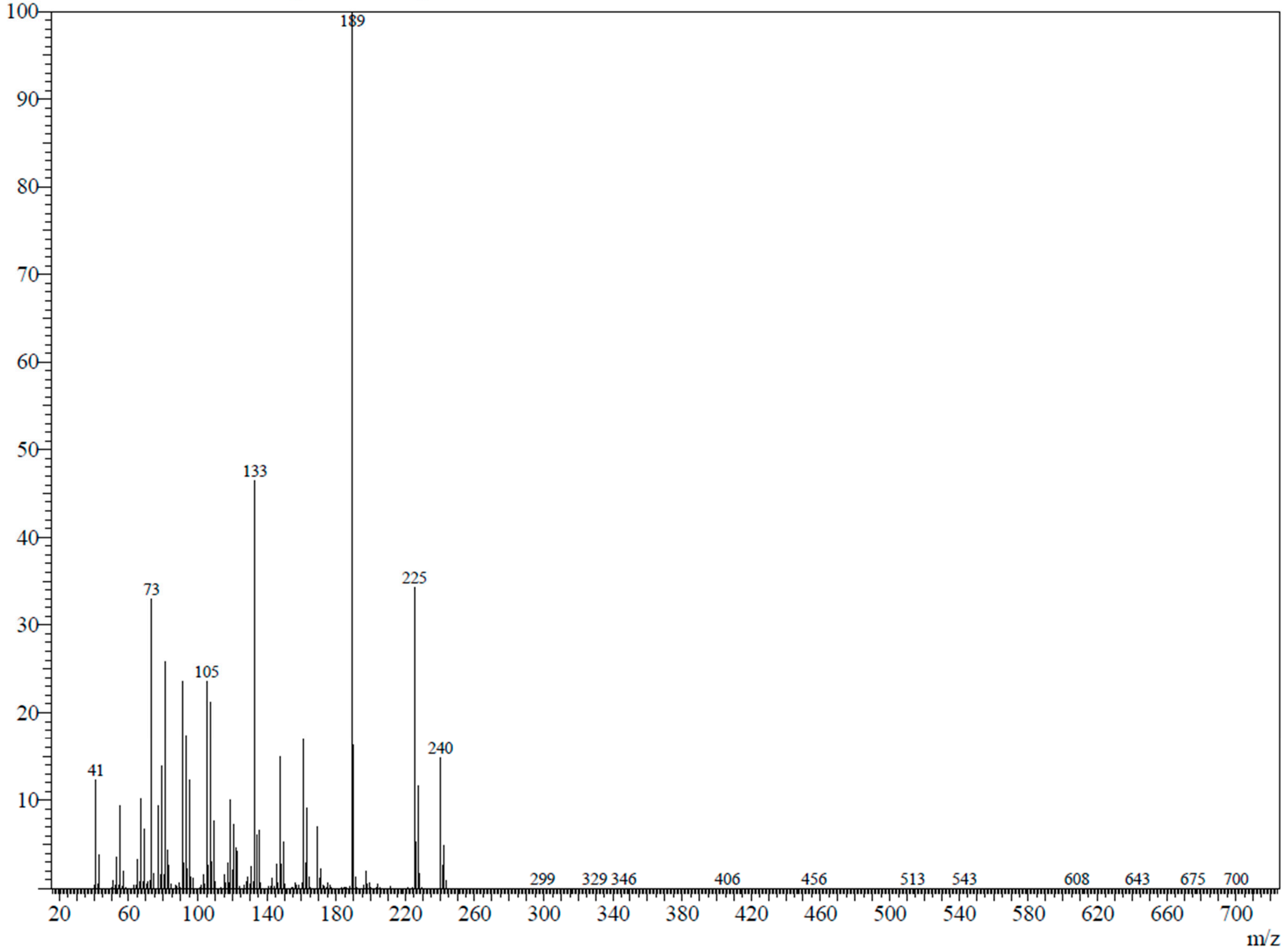

Myristic acid

Line#:37 R.Time:11.210(Scan#:1443)
MassPeaks:361
RawMode:Averaged 11.205-11.215(1442-1444) BasePeak:74(126629)
BG Mode:Calc. from Peak Group 1 - Event 1 Q3 Scan

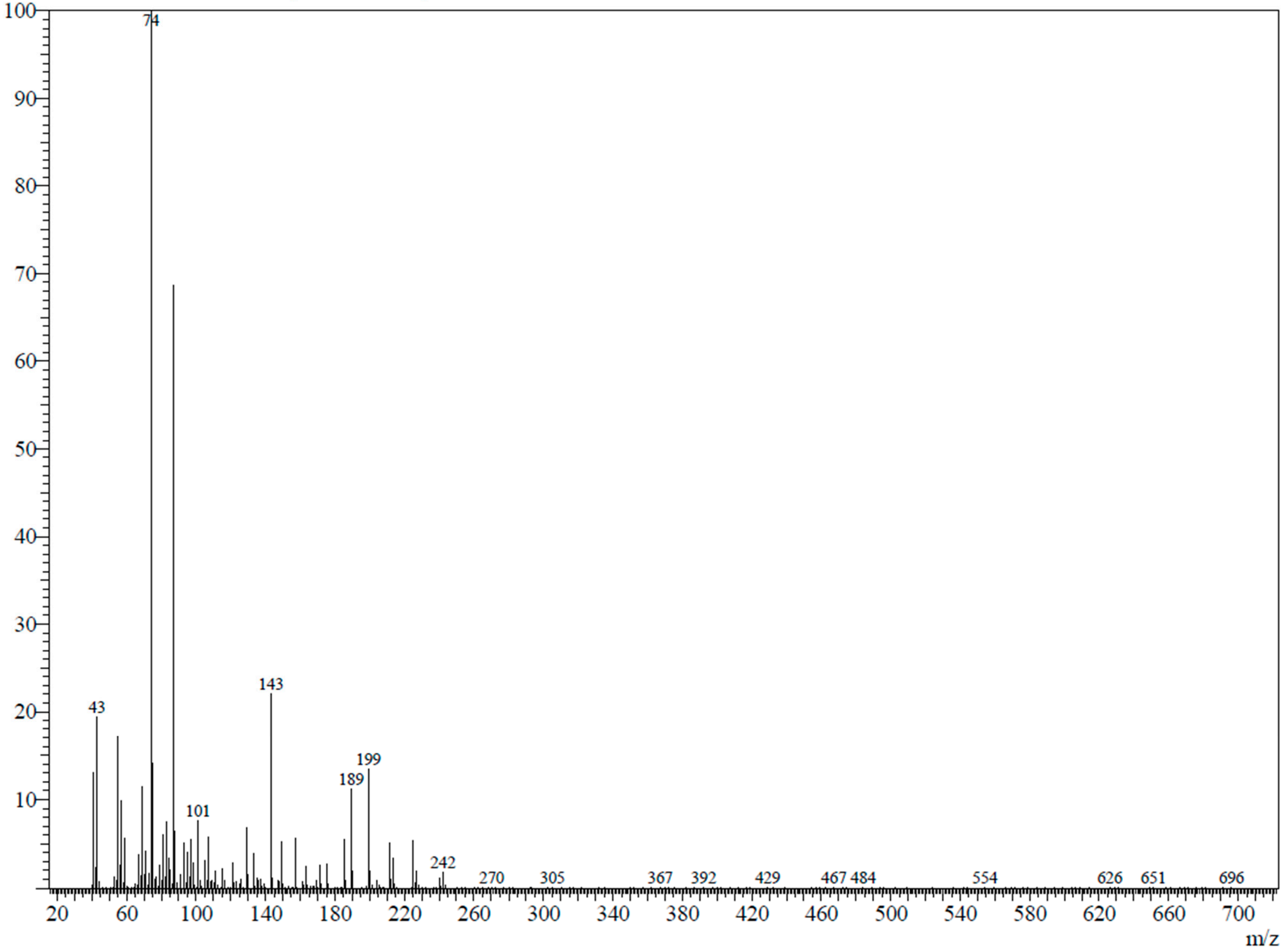

Palmitoleic acid

Line#:43 R.Time:14.070(Scan#:2015)
MassPeaks:437
RawMode:Averaged 14.065-14.075(2014-2016) BasePeak:55(377071)
BG Mode:Calc. from Peak Group 1 - Event 1 Q3 Scan

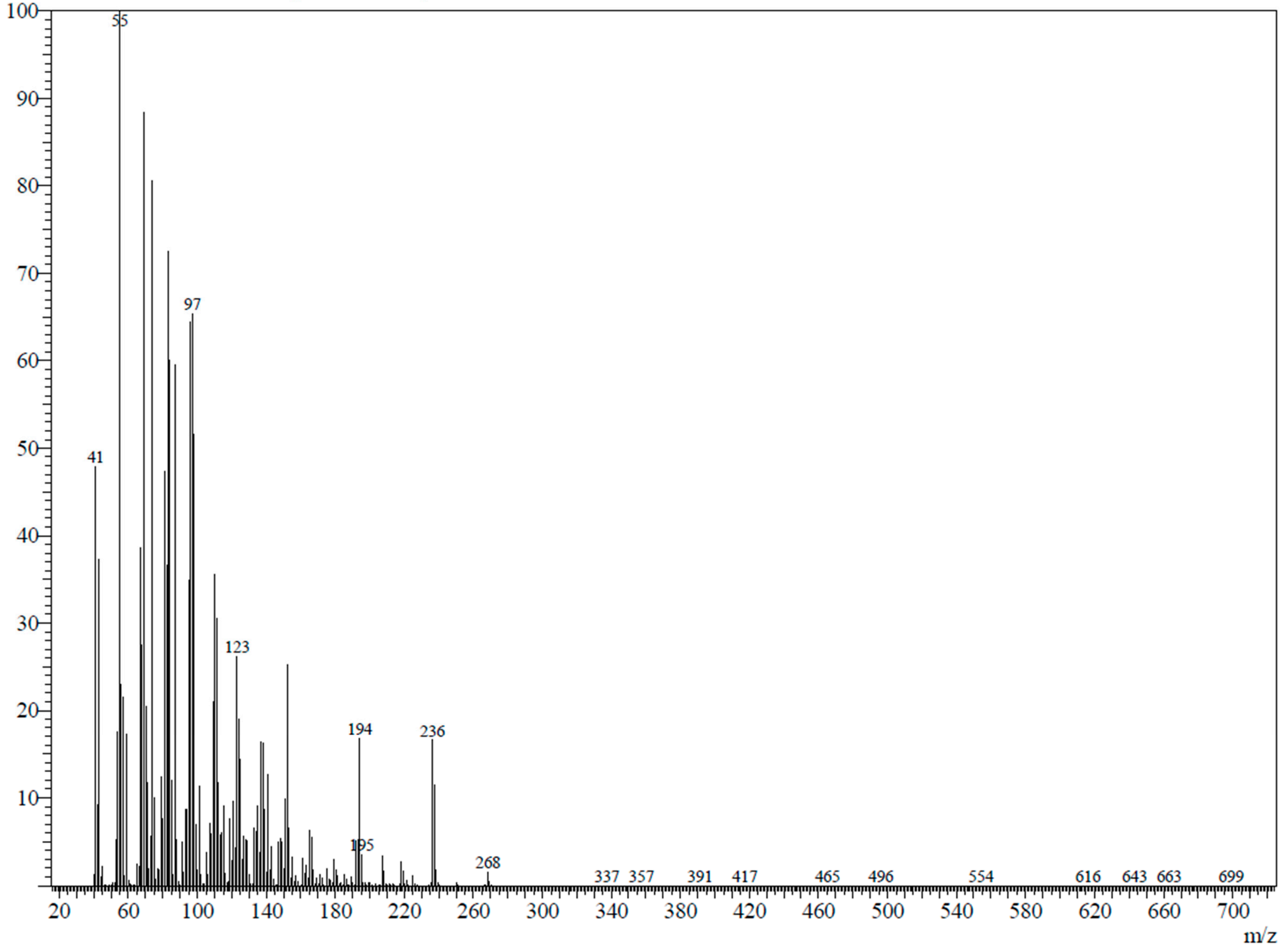

Palmitic acid

Line#:44   R.Time:14.480(Scan#:2097)
MassPeaks:458
RawMode:Averaged 14.475-14.485(2096-2098) BasePeak:74(7350479)
BG Mode:Calc. from Peak Group 1 - Event 1 Q3 Scan

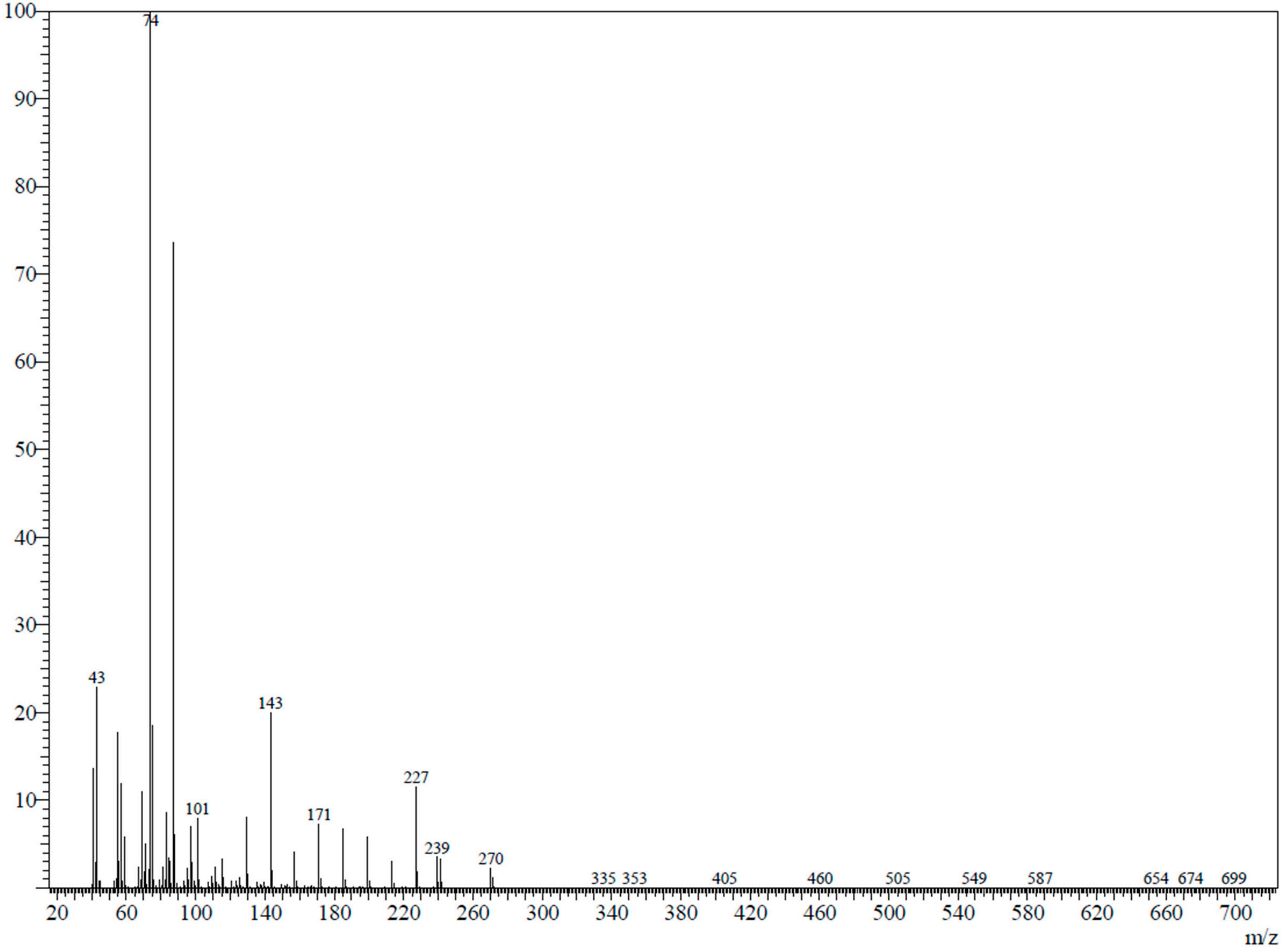

Linoleic acid

Line#:45 R.Time:17.550(Scan#:2711)
MassPeaks:433
RawMode:Averaged 17.545-17.555(2710-2712) BasePeak:81(648140)
BG Mode:Calc. from Peak Group 1 - Event 1 Q3 Scan

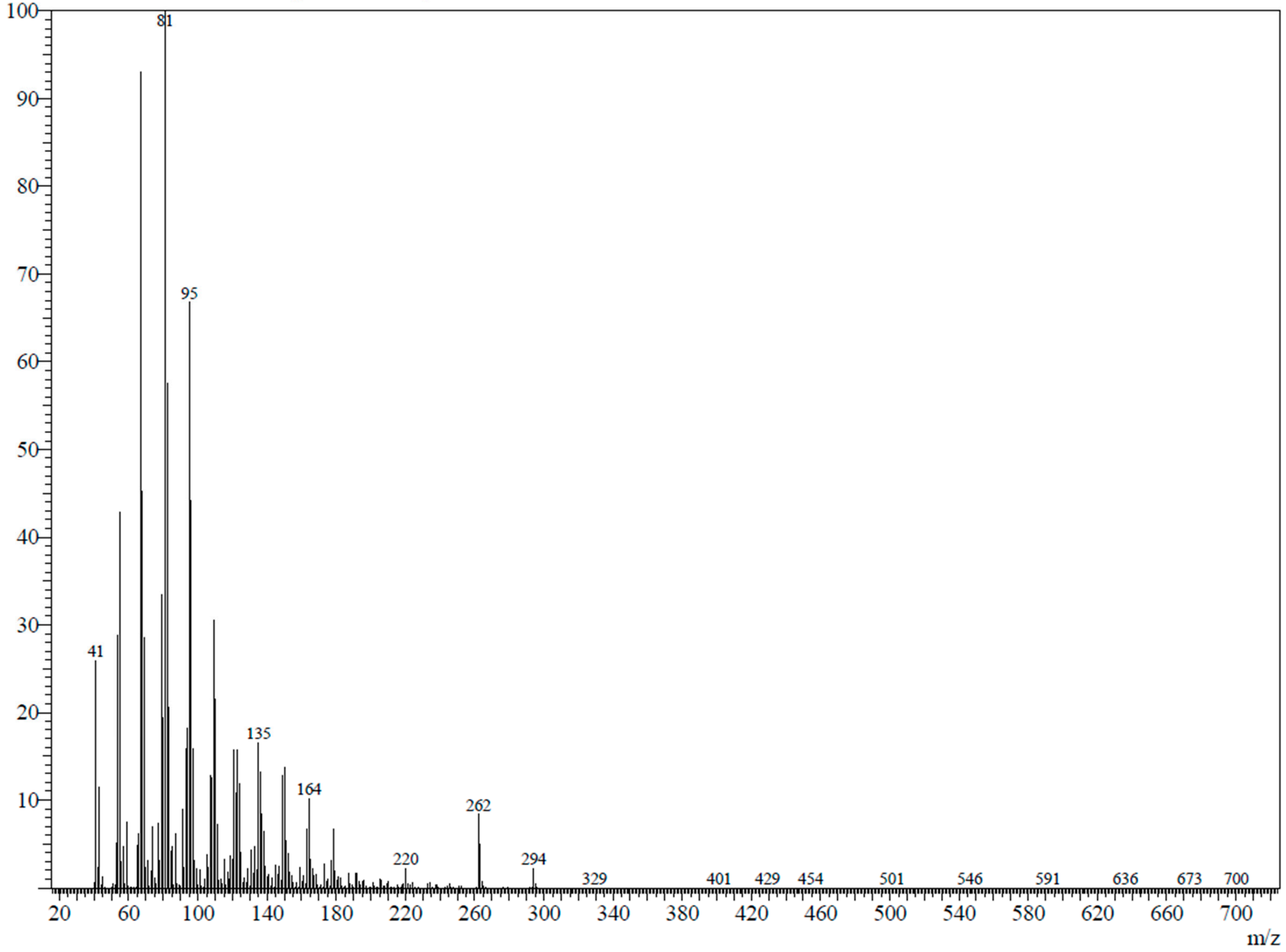

Oleic acid

Line#:46 R.Time:17.720(Scan#:2745)
MassPeaks:444
RawMode:Averaged 17.715-17.725(2744-2746) BasePeak:55(1488110)
BG Mode:Calc. from Peak Group 1 - Event 1 Q3 Scan

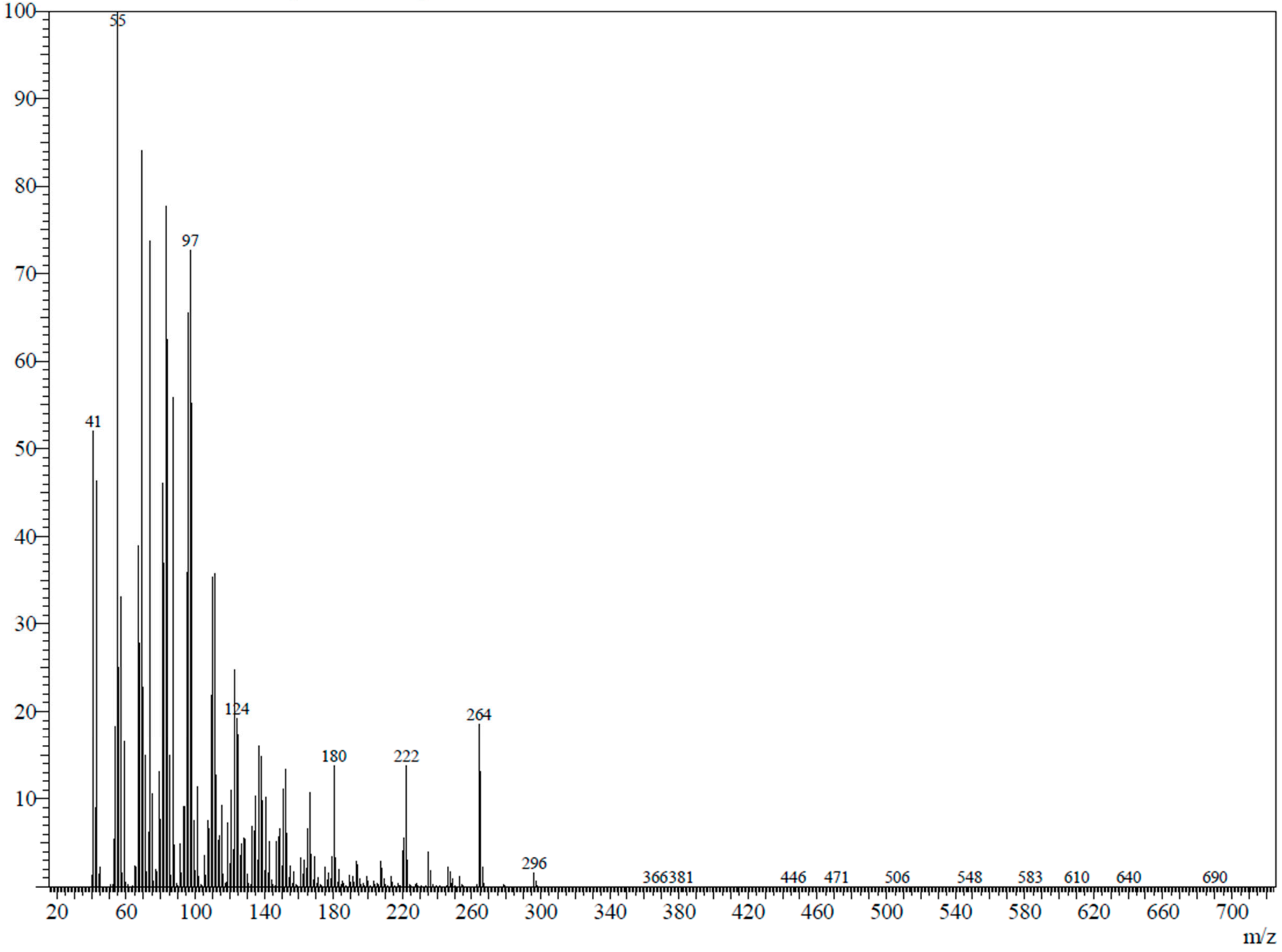

Elaidic acid

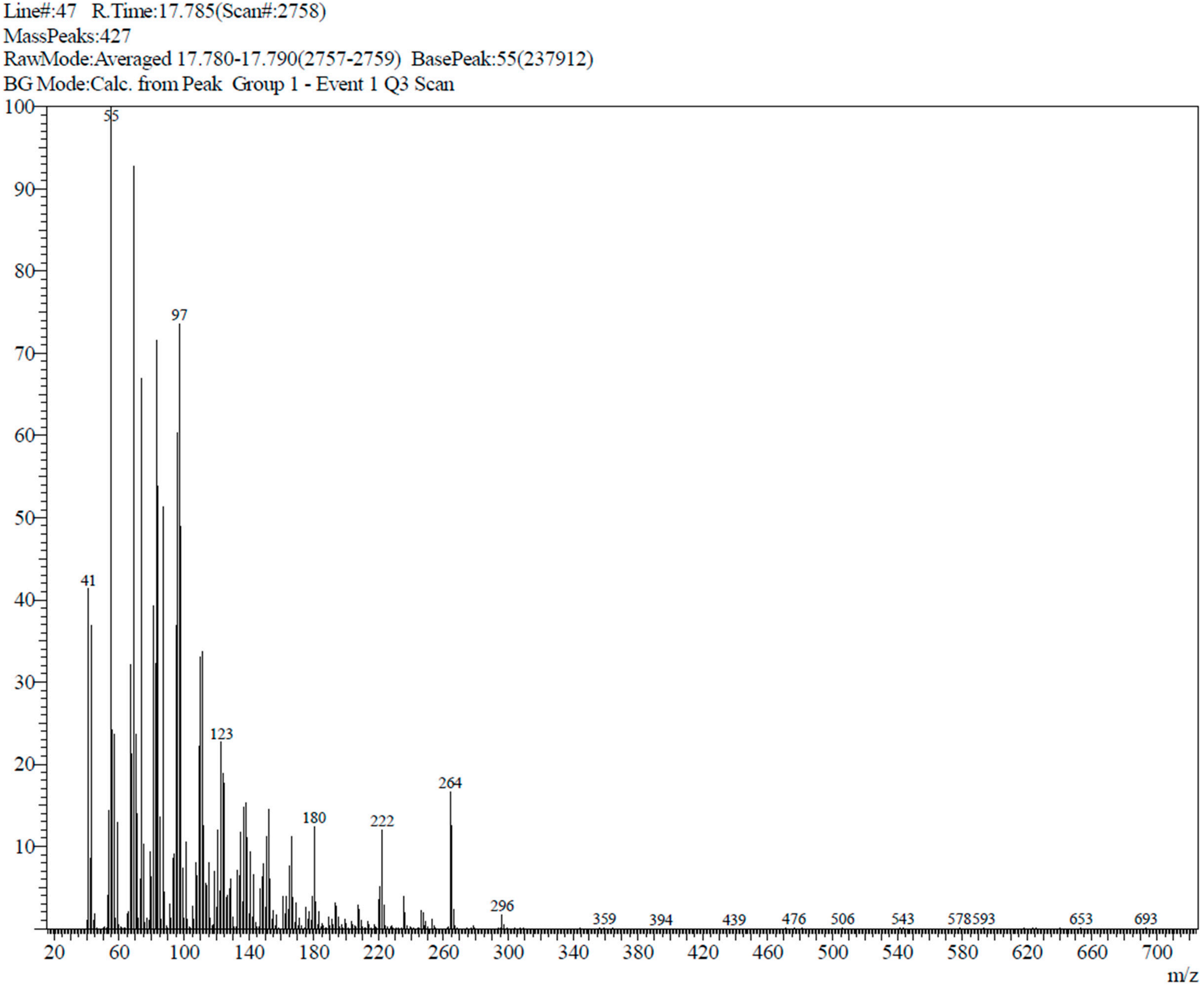

Stearic acid

Line#:48 R.Time:18.150(Scan#:2831)
MassPeaks:412
RawMode:Averaged 18.145-18.155(2830-2832) BasePeak:74(708225)
BG Mode:Calc. from Peak Group 1 - Event 1 Q3 Scan

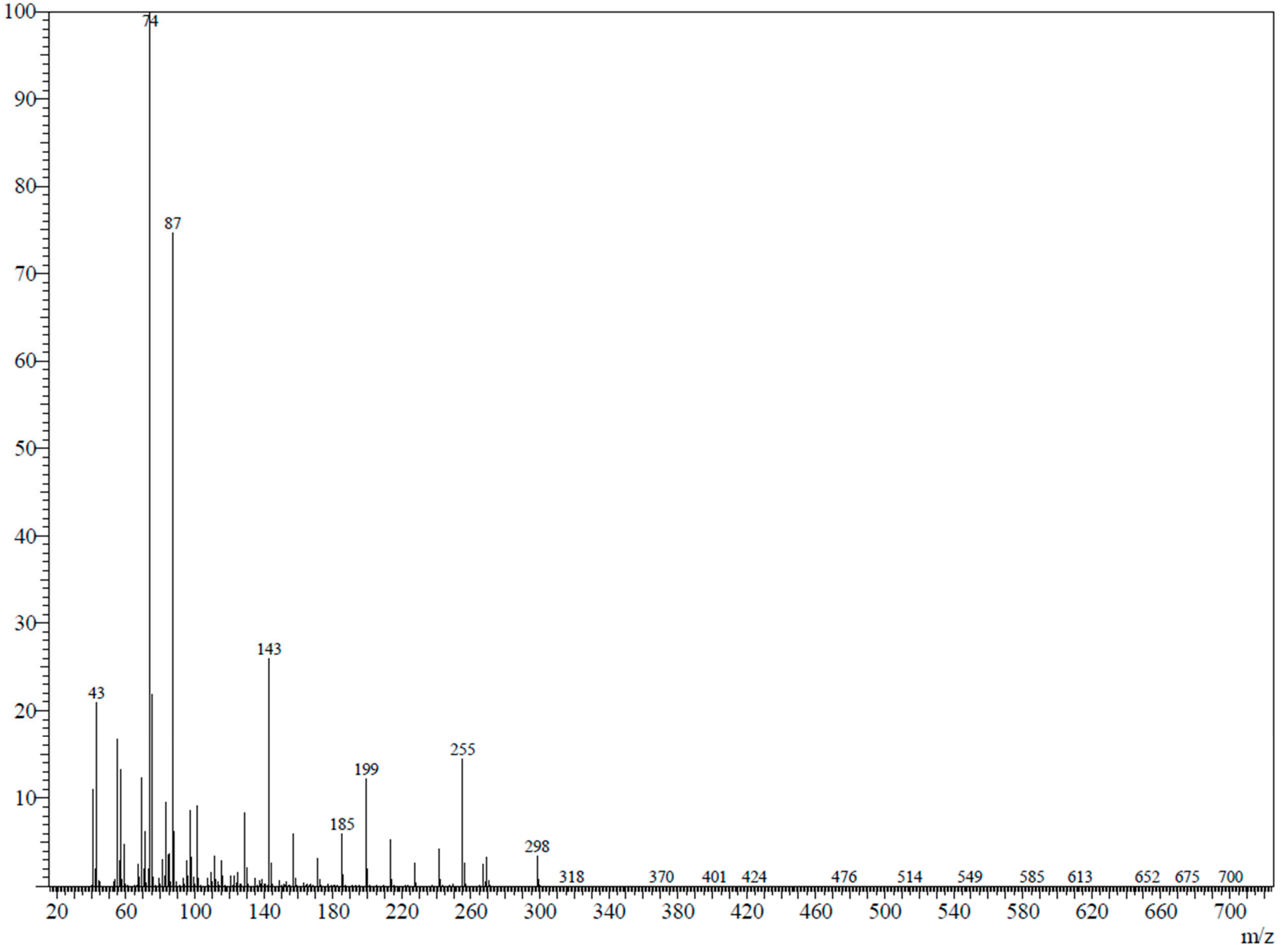

Eicosanoic acid

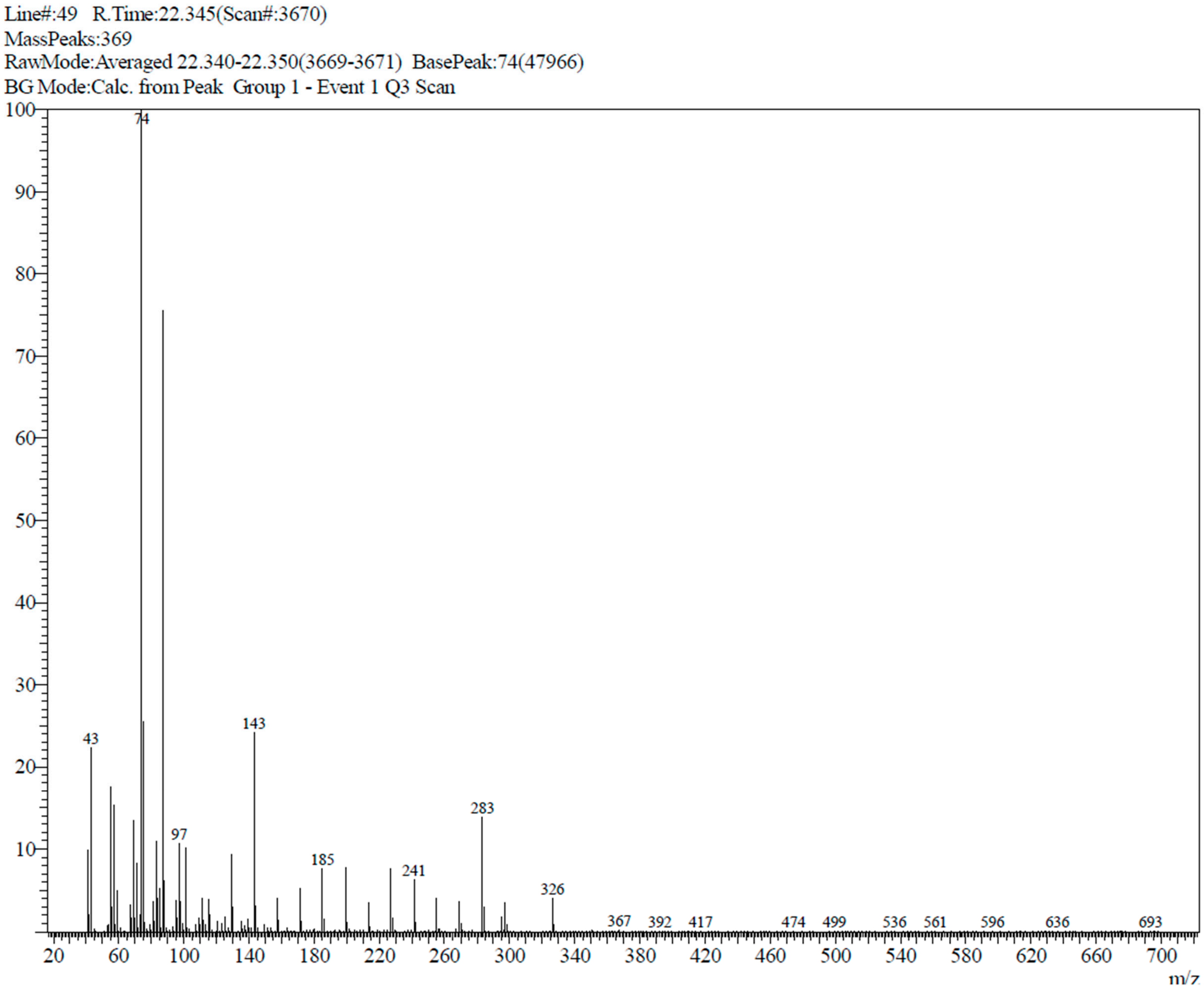

Supplement: Supplementary file 1 [file molecules-31-03705-s001.zip › molecules-4555185-supplementary.pdf]
